# Diversity of root-associated culturable fungi of *Cephalanthera rubra* (Orchidaceae) in relation to soil characteristics

**DOI:** 10.7717/peerj.8695

**Published:** 2020-03-02

**Authors:** Jake Bell, Kazutomo Yokoya, Jonathan P. Kendon, Viswambharan Sarasan

**Affiliations:** 1 Natural Capital and Plant Health, Royal Botanic Gardens, Kew, Richmond, Surrey, UK; 2 Comparative Plant and Fungal Biology, Royal Botanic Gardens, Kew, Richmond, Surrey, UK

**Keywords:** Mycorrhiza, Culture-dependent, Tripartite, Plant-fungus interaction, Tree-orchid relationship, Woodland, Terrestrial habit

## Abstract

*Cephalanthera rubra* (L.) Rich., Red Helleborine, is a widespread orchid in Europe but known only from three very small populations in England. These populations are in decline with no natural seed setting for more than a decade. The species may become extinct in the UK soon unless viable strategies are in place for ex situ conservation, especially the use of symbiotic propagation. Because of the fragile nature of the populations in England mycorrhizal fungal diversity study is not feasible. Therefore, to understand the factors needed for healthy Red Helleborine populations, soil characteristics and diversity of culturable root-derived fungi of the populations from a small area in the Loire Valley in France were studied. The main objectives of the study were: (1) Which culturable mycorrhizal fungi associated with *C. rubra* roots and (2) To what extent is variation in fungal communities related to variation in soil characteristics? Here, we report a significant difference in diversity of culturable mycorrhizal and non-mycorrhizal fungi depending on soil pH and phosphorus content. Mycorrhizal associations were favoured by plants in locations with low soil nutrient availability and comparatively higher pH. Our study shows that mycorrhizal fungi, both ecto and endo, can be cultured from roots of plants at different maturity stages.

## Introduction

With about 28,000 species in 736 genera worldwide, Orchidaceae is one of the largest families of flowering plants (Christenhusz & Byng, 2016). Orchid biodiversity loss has been attributed to factors such as climate change, anthropogenic movement, collecting of wild orchids, land clearance, and issues with pollination linked to climate change (Barman & Devadas, 2013; Willmer, 2014). Additionally, inherent properties of certain orchid species, such as life span, height when in flower, and environmental factors such as pH, have been shown to correlate with species loss (Kull & Hutchings, 2006). *Cephalanthera rubra* (L.) Rich., Red Helleborine, is widespread in Europe but known only from three populations in England (Fay & Taylor, 2015). This means this species is critically endangered in England (Rankou, 2011). The Red Helleborine is normally located in woodlands (particularly those rich in beech and oak), woodland edges, and grasslands (Rankou, 2011; Jakubska-Busse, Pielech & Szczesniak, 2014).

Seed germination in terrestrial orchids is reliant on the interaction with a suitable orchid mycorrhizal fungus (OMF) while in taxa such as *Cephalanthera* further symbiotic association with ectomycorrhizal fungi (EMF) associated with tree roots is equally important (Bidartondo & Read, 2008). In orchids that possess photosynthetic cells, they may either become fully autotrophic, or obtain carbon from both mycoheterotrophy and photo assimilation (Bidartondo et al., 2004; Preiss, Adam & Gebauer, 2010), a nutritional method coined mixotrophy (Julou et al., 2005). *Cephalanthera* is a genus that contains examples of both mixotrophy and full mycoheterotrophy (Merckx et al., 2013). The extent to which mixotrophic orchids use their mycobionts is variable and can depend on factors such as available sunlight (Preiss, Adam & Gebauer, 2010).

In *C. damasonium* (Mill.) Druce and *C. longifolia* (L.) Fritsch, based on culture-independent methods, wide range of fungi were found to be involved in germination (Bidartondo & Read, 2008), while Pecoraro et al. (2017) uncovered a low degree of mycorrhizal specificity in the roots of adult plants. The current study aims to identify fungi that can be cultured and used for future seed germination, by isolating fungi from pelotons in root samples. This approach was preferred over the seed baiting methods as successful seed baiting may take years (Bidartondo & Read, 2008) while collecting root samples from plants at different maturity is faster (Yokoya et al., 2015). This may provide a list of potential symbionts that can be used for time-bound projects.

As populations in England only sprout and flower sporadically, they are not always available for isolating fungi from roots. Therefore, small healthy *C. rubra* populations in the Chaumussay area in the Loire Valley, France were identified for collecting root materials to isolate culturable fungi. Being closest geographically and climatically to the sites in England, these populations were a suitable match to orchid populations in England. This study was undertaken to isolate, culture and identify fungi from plants found in different population in different soil types. As spontaneous seedlings at very early stages of development are almost impossible to locate, this study targets plants at later stages of development, and flowering plants. Using plants at later stages of development, therefore, our aim is to understand the nature of association between the orchid and mycorrhiza, whether generalist or specialist in nature, in relation to soil characteristics.

## Materials and Methods

### Site description

Healthy populations of Red Helleborine used in this study are from a managed private woodland site by the river La Muanne, a tributary of the river Claise which finally flows into the Loire. Cultivated land lies between the woodland site and the river. This land was lying fallow at the time of this study following bean cultivation in the previous year. Wildflower meadow species grow at the field margin and unmanaged woodland spreads from the field boundary up towards a rocky outcrop. Woodland was previously used for grazing (sheep) and very small-scale quarrying for stone (about 100 years ago). Trees present are mainly downy oak (*Quercus pubescens* Willd.), with juniper (*Juniperus communis* L.) at woodland edges. Humus layers of 10–20 cm depth comprising leaf mould and spent fruits of downy oak cover the ground, underneath which the soil is heavily compacted and stony, except at the quarry where humus is absent. The site is at the boundary of late Cretaceous white chalk or Tuffeau blanc de Touraine and yellow limestone or ‘Tuffeau jaune de Touraine’. The colour in the yellow limestone is derived from the presence of sand (InfoTerre, 2013). The site is around 100 m above mean sea level, with a south-facing slope of approximately 20 degrees. Ground vegetation is sparse, with significant percentage of bare ground. Fescue, ivy, oak seedlings and nettles are present.

### Population description

**Figure 1:**
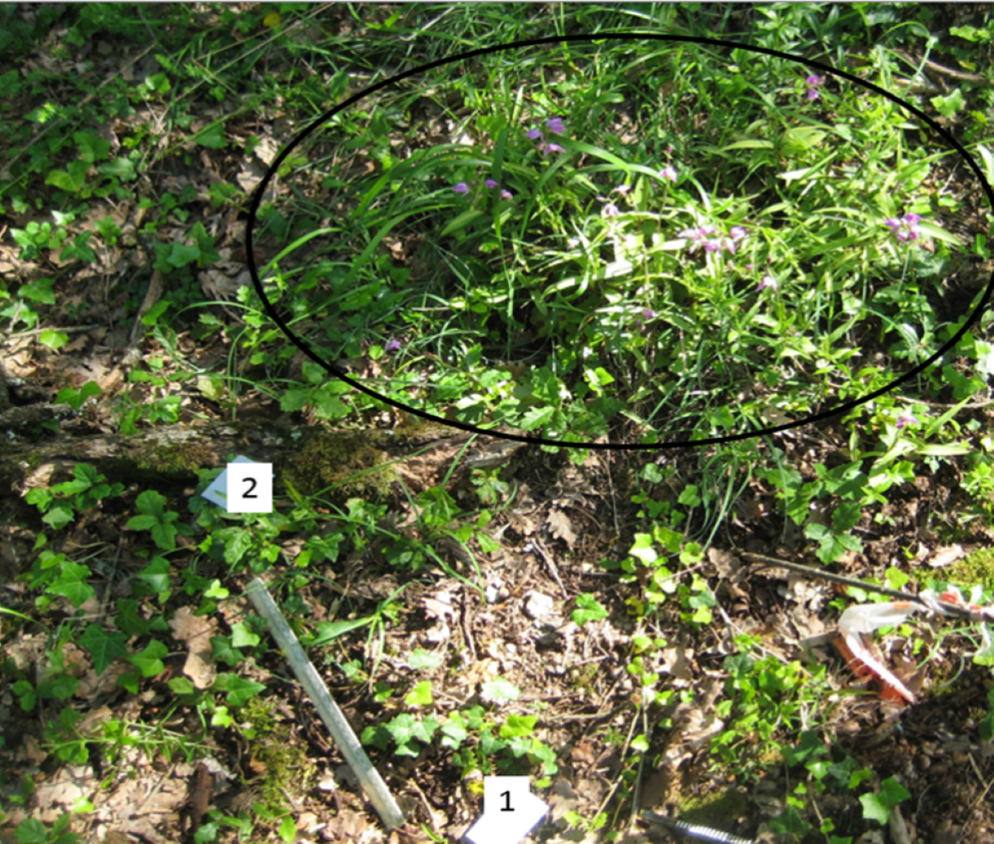
Location A—positions of Plant numbers 1 and 2 relative to the main clump of *Cephalanthera rubra* (circled). Ruler (30 cm) for scale. Figure source credit: Jonathan P Kendon.

Range extent for *C. rubra* in this area is a few hundred square metres. Two main clumps of over 30 plants each were found (Locations A and D) 45 m apart between which were scattered smaller groups of individuals (Locations B and C) (Fig. 1). The clumps consisted of flowering and non-flowering shoots which may have been clonal off-shoots. Near to the clumps were smaller shoots. Roots of these plants were preferentially collected because these seedlings were more likely to have derived from seed germination and therefore harbour mycorrhizal fungi.

### Collection and transport of root material, and culture of fungal isolates

*Cephalanthera rubra* is a protected species in the Centre-Val de Loire region of France. A collecting permit was granted by the Direction Départementale des Territoires in the prefecture of Tours governing the Indre-et-Loire department with support from Unité Forêt et Biodiversité of the Service de l’Eau et des Ressources Naturelles. Mr. and Mrs. Bernard granted permission to collect plant materials on their private property. This allowed individual roots to be excised from plants with minimal soil disturbance. To ensure viable pelotons were acquired, younger roots were collected when available. Once detached from the plant at the root base, each root was placed over pre-moistened sterile cotton wool within a pre-sterilized glass vial. To reduce contamination of cultures by soil fungi and bacteria, a quick surface-sterilisation was performed prior to fungal isolation. Colonised root sections of at least 1 cm in length were dipped for one minute into 0.5% sodium dichloroisocyanurate with a drop of Tween 20 (Sarasan et al., 2006). Root sections were then rinsed three times in sterile de-ionised water. Fungi were then isolated from root sections using the method of Zettler, Sharma & Rasmussen (2003). Pelotons and macerated root pieces were collected from root samples within a week of collecting the root samples. Colonisation of fungi in the cortical region of the roots was recorded (estimated percentage of cortical cells with pelotons based on <1 mm transverse section and comprising 150–300 cells). Transverse sections of <1 mm were made at approximately 10 mm intervals along each root and the sections observed under a stereo microscope. Where a section showed cortical cell colonisation the neighbouring 10 mm slice was selected for fungal isolation. The number of slices selected for culturing varied from one to nine for all the roots. Macerated cortical cells containing pelotons were transferred to fungal isolation medium (FIM; Mitchell, 1989) containing streptomycin sulphate (Clements & Ellyard, 1979) and incubated at 18 °C. After 1–4 days, emerging hyphae observed from cortical cells/pelotons under a dissection microscope were transferred to fresh FIM. All fungi were picked and isolated from this initial culture, and orchid mycorrhizal fungi were tentatively identified by morphology according to Watanabe (1994).

### Soil analysis

One root zone soil sample was collected from each of the eight plant sites (Table 1), transported to the Royal Botanic Gardens, Kew (RBG Kew) and analysed using LaMotte STH14 Combination Soil Testing Outfit (LaMotte, Chestertown, MD, USA). Variables tested were pH, phosphorus (P), nitrate nitrogen (N) and organic matter (OM). A HI 2550 pH metre (Hanna Instruments, Leighton Buzzard, UK) was used once initial pH testing steps had been followed using the kit, to achieve a higher accuracy than possible using the kit’s indicator solutions.

**Table 1:** Description for collected roots of Cephalanthera rubra.

Plant number	Location	Location description
1	A	Plant 48 cm from main clump
2	A	Plant 28 cm from main clump
3	A	Plant 2.8 m from main clump. At base of *Quercus* trunk
4	B	Seedling and 1 flowering plant within 1 m of each other, and 10 m from population A. A total of 20 plants within 10 m along rising pathway
5	C	Below rock outcrop (small disused quarry). In shade
6	D	Ridge above outcrop near *Quercus* saplings. Four other plants nearby, including three flowering
7	D	Below rock outcrop (small disused quarry). Deep shade. One other seedling nearby
8	D	Mature plant near clump of 30 seedlings. Sloping below path

### Molecular identification

DNA was extracted from the mycelium of each cultured fungal isolate. To identify fungal isolates, the internal transcribed region of the rRNA gene (ITS) was sequenced (Yokoya et al., 2015). The DNA was amplified by PCR with primers ITS1 and ITS4 (White et al., 1990). PCR amplicons were then directly sequenced via Sanger sequencing with the forward and reverse primers on an Applied Biosystems 3730xl DNA Analyzer (Thermo Fisher Scientific, Waltham, MA, USA). Sequence analyses were carried out using Geneious^®^ software (Biomatters, Auckland, New Zealand). Sequences were aligned, and accuracy and consensus were checked for both the forward and reverse primers. Sequences with ≥97% similarity were grouped into operational taxonomic units (OTUs), and following this, a BLAST (National Centre for Biotechnology Information, Bethesda, MD, USA) search was carried out to identify isolates based on named database entries in GenBank using a sequence similarity threshold of 97%.

### Statistical analysis

Fungal distribution in roots of different plants at different Locations (Table 1) was assessed using R version 3.3.2 (R Core Team, 2016). Multivariate analysis was carried out for fungal distribution and four fitted environmental gradients (soil pH, phosphorus (P), (N) and (OM)) through non-metric multidimensional scaling (NMDS) via the *metaMDS* and *envfit* functions in the vegan package (Oksanen et al., 2017). A second data set with environmental measurements of soil pH, P (ppm), N (ppm) and OM (1–5 scale) were used to fit environmental vectors onto the ordination plot using the *envfit* function. The significance of the four fitted environmental vectors (*p* value) is determined through permutation of environmental variables. The goodness of fit statistic is squared correlation coefficient (*r*^2^). Additionally, P was also used to fit ellipses onto the ordination plot to show its effect on fungal distribution (Dataset S1). Phylogenetic analysis was undertaken using Geneious^®^ software (Biomatters, Auckland, New Zealand). Neighbour-joining trees were created using the tree building function with OTUs of both endo and EMF which are involved in seed germination and networking with tree roots. The closest five matches on GenBank for each endomycorrhizal isolate and closest 10 matches for ectomycorrhizal isolates (for which host and/or location information were available) were also used, along with representative sequences previously reported from *Cephalanthera*. Bootstrap percentages were used to show support >50% after 1,000 replications. Representative DNA sequences (96 sequences) were deposited in GenBank with accession numbers Seq1 SUB6288079 (MN450567) to Seq96 SUB6288079 (MN450662).

## Results

### Plant morphology

Morphology of the collected plants from four Locations was recorded (Table 2). Location D had the tallest individual plant (Plant number 8, 220 mm), as well as the greatest mean height (150 mm). Root zone depth ranged from 30 to 60 mm in all plants except for Plant number 1 (Location A), which had a far greater depth of 200 mm. Plant numbers 1–6 (Locations A–D) had 3 leaves and 3 roots, whilst Plant number 7 (Location D) had five leaves and five roots, and Plant number 8 (Location D) had four leaves and four roots. Each plant that performed best in each growth parameter, also yielded mycorrhizal fungi (Table 3).

**Table 2:** Plant morphology of *Cephalanthera rubra* from Loire Valley, France used to study root associated fungal diversity.

Plant number	Location	Plant height (mm)	Root zone depth (mm)	No. leaves	No. roots
1	A	60	200	3	3
2	A	110	50	3	3
3	A	60	40	3	3
4	B	100	30	3	3
5	C	100	50	3	3
6	D	100	50	3	3
7	D	130	50	5	5
8*	D	220	60	4	4

**Note:**

* Plant with inflorescence.

**Table 3:** Fungal diversity and environmental factors across four Locations (eight plants) of *Cephalanthera rubra* from Loire Valley, France.

Plant number (Location)	Number of root slices	Root slice colonisation (%)	Endophytic Fungal isolates	Mycorrhizal fungal isolates (%)	Soil pH	Soil P (ppm)	Soil N (ppm)	Soil OM^a^
1 (A)	6	70, 0, 0, 70, 70, <10	56	4 (6.7)	7.8	25	75	5
2 (A)	6	10, 10, 30, 70, 10, 30	92	0 (0)	7.7	38	20	4
3 (A)	5	<10, 0, 70, 20, 50	46	2 (4.2)	7.7	50	50	4
4 (B)	3	20, 0, 0	17	0 (0)	8.1	13	3	2
5 (C)	9	All 100	162	0 (0)	7.7	25	3	3
6 (D)	1	30	5	1 (16.7)	8.42	13	20	1
7 (D)	4	20, 10, 20, 50	17	53 (75.7)	8.2	13	3	1
8 (D)	2	70, <5	11	2 (15.4)	7.9	13	10	3

**Note:**

a OM, organic matter; scaled 1–5—1, very low; 2, low; 3, medium; 4, high; 5, very high.

### Collection and transporting of root materials

Roots were successfully transported back to RBG Kew for isolating fungi. Plant number 5 demonstrated the highest level of root colonisation, with all nine root slices showing 100% colonisation. This translated to the highest number of isolated fungi (162), all of which were non-mycorrhizal endophytes, fungi which live inside tissues asymptomatically. In all other plant roots, colonisation of slices varied from <5% to 70%. The lowest level of colonisation was seen in plant 4, with 20%, 0% and 0% seen for the three slices (Table 3).

### Molecular identification of fungi

In total, 468 full or near-full length ITS sequence results were obtained from fungal isolates, which grouped into 96 OTUs. Isolates were identified to family/genus/species level, dependent on the identity of available matching entries in the GenBank database.

### Soil analysis

Location A had moderately dry, loose soil with the presence of leaf mould (Plant number 1-Location A), grass leaves (Plant number 2-Location A) and sand grains (Plant number 3-Location A). Location B had a mix of damp clay and low levels of coarse OM. Soil from Location C had wet, heavy clay and small chalk stones of <1 cm. Plant numbers 6 and 7 from Location D shared wet, heavy clay soil mixed with small chalk stones <1 cm diameter, whilst Plant number 8, also from Location D, was growing in a mix of damp clay and coarse OM. The average pH for Location D was more alkaline than the other locations, presumably due to the high chalk content. Overall, soil parameters varied both within and between Locations (Table 3).

### Fungal diversity and distribution

Fungal OTUs were subject to NMDS analysis, representing 36 root slices from eight plants, collected from 4 Locations. Of the total five mycorrhizal OTUs (Table 4) three of them were orchid endomycorrhizae (one Ceratobasidiaceae, and two *Ceratobasidium*), and two ectomycorrhizae (one *Cenococcum geophilum*, one *Tomentella*). A high number of non-mycorrhizal endophytic fungi (those fungi live inside plant cells asymptomatically) were also recovered. These included *Cadophora*, *Cladophialophora*, *Exophiala*, *Cyphellophora* and *Phomopsis*.

**Table 4:** Operational taxonomic units (OTUs) of mycorrhizal fungi isolated from roots of *Cephalanthera rubra*, determined through ITS sequencing and BLAST search.

OTU ID	Plant number (location)	Closest named match in GenBank	Identity (%)
Cerae1^a^	1 (A)	DQ182460 uncultured Ceratobasidiaceae isolate	98.1
Cerbium1^a^	3 (A)	KX610453 *Ceratobasidium albasitensis*	99.8
Cerbium2^a^	6, 7 (D)	KJ188567 uncultured *Ceratobasidium* clone	98.6
Ceno2^b^	1 (A)	KC967384 *Cenococcum geophilum*	99.4
Tom1^b^	8 (D)	JX625354 uncultured *Tomentella* clone	99.4

**Notes:**

a Endo-mycorrhizal.

b Ecto-mycorrhizal.

**Figure 2:**
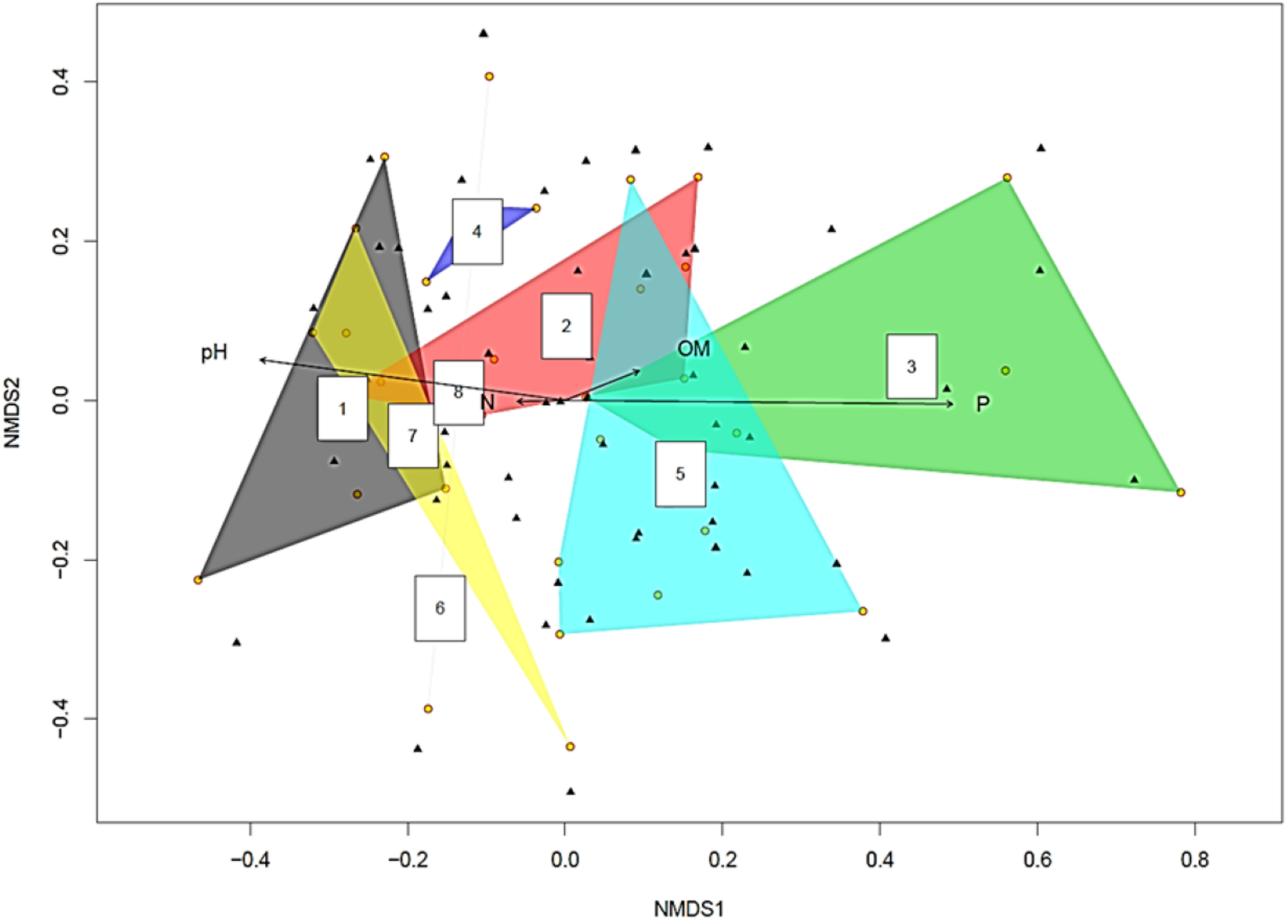
Non-metric multidimensional scaling (NMDS) plot showing root associated endophytic fungal diversity in eight representative samples of *Cephalanthera rubra*. Data from four Locations in Loire Valley, France, along four environmental gradients: pH, P (ppm), N (ppm) and level of organic matter (OM). Yellow filled circles represent individual root slices, whilst filled black triangles represent individual fungal OTUs. Two of the four fitted environmental gradients showed statistical significance on the ordination plot (pH, *p* < 0.05 and P, *p* < 0.001). A polygon with five yellow circles contains data from five root slices while a polygon created by joining three yellow circles contains data from three root slices.

The yellow circles represent root slices. A polygon with five yellow circles contains data from five root slices while a polygon created by joining three yellow circles contains data from three root slices (Fig. 2). The NMDS analysis showed soil pH and P concentration had statistically significant effects on fungal diversity (*p* < 0.05 and *p* < 0.001 respectively). This became clearer when the fungi were split into two groups based on the pH of the soil they were isolated from Supplemental Information on pH > 8 and pH < 8 (Tables S1 and S2). Recovered fungi from plants in soil with pH < 8 (Table S2), the dominant fungi were *Cadophora* sp. (14.7%) followed by *Penicillium* sp. (13.3%), Hypocreales sp. (12.5%) and *Acremonium* sp. (8.5%), whilst the mycorrhizal fungi Ceratobasidiaceae (0.5%), *Cenococcum geophilum* (0.5%), *Ceratobasidium* (0.5%) and *Tomentella* (0.5%) were rare. However, when the pH was >8 (Table S1), this relationship was inverted, with the mycorrhizal fungi *Ceratobasidium* being the dominant fungi (58.1%), surpassing *Cadophora* (20.4%).

**Figure 3:**
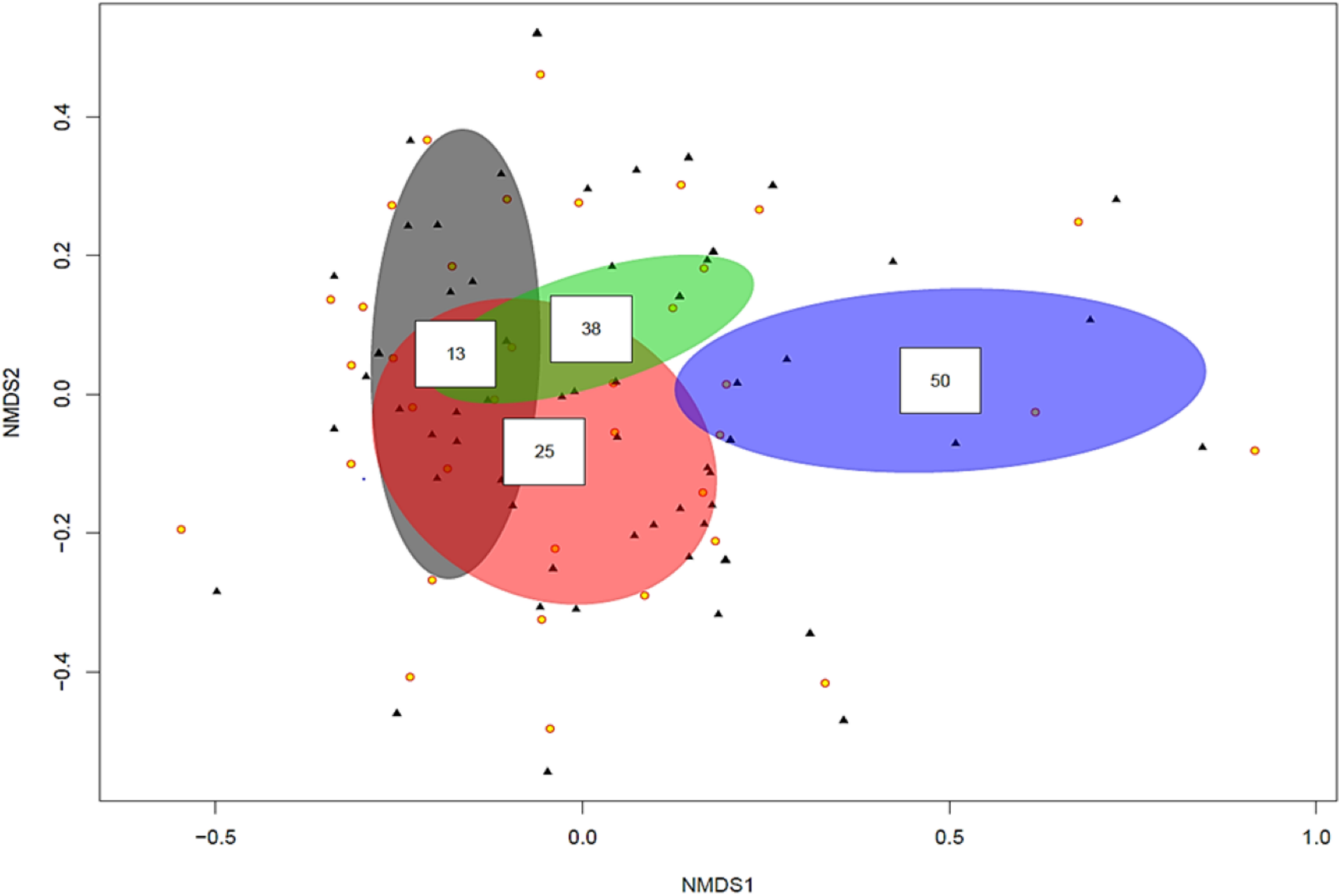
Non-metric multidimensional scaling (NMDS) plot showing root associated endophytic fungal diversity in selected eight representative samples of *Cephalanthera rubra*, from four Locations in LoireValley, France. Yellow filled circles represent individual root slices, whilst filled black triangles represent individual fungal OTUs. Ellipses represent fungal diversity in four P soil concentrations (i.e. 13, 25, 38 and 50 ppm). **** OTUs inside an ellipse are highly correlated with that soil P concentration, and those outside are present at several soil P concentrations.

The ordination plot with overlapping ellipses showing fungal diversity in relation to soil P concentration (Fig. 3), revealed P at 50 ppm (Plant number 3; pH 7.7) yielded the highest amount of ‘unique’ fungi (smallest overlap with other concentrations), whilst fungi found in soils with P concentrations of 13 ppm (Plant numbers 4, 6, 7 and 8-Locations B and D) demonstrated the largest overlap with 25 ppm (Plant numbers 1 and 5-Locations A and C) and 38 ppm (Plant number 2-Location A). OTUs inside an ellipse are highly correlated with that of soil P concentration, and those outside are present at several soil P concentrations. Plant number 7, which was found in soils with a P concentration of 13, also yielded the highest number of mycorrhizal isolates, all of which being *Ceratobasidium*. In general, lower fungal diversity and colonisation was seen when soil pH was high and P was low, such as plants from Location D (average pH of 8.17 and P of 13 ppm) which also had the highest colonisation of mycorrhizal fungi *Ceratobasidium* (Plants 6 and 7-Location D) and *Tomentella* (Plant number 8-Location D), compared to higher diversity and colonisation from Locations with lower pH and high P levels, such as Plant number 3 (P; 50 ppm and pH; 7.7). Neighbour-Joining analysis revealed the mycorrhizal sequences obtained in this study were closely related to those isolated from *Cephalanthera* and other orchid species Supplemental Information: phylogeny trees of *Ceratobasidium* (Fig. S1), *Tomentella* (Fig. S2) and *Cenococcum* (Fig. S3).

## Discussion

Generally, *C. rubra* grows in shade on chalky, humus-rich soils in habitats with deciduous and coniferous forests. The population studied was found on a shaded site in Loire Valley, France. NMDS analysis of the fungal diversity in this population showed statistical significance on the ordination plot for two environmental factors: pH (*p* < 0.05) and P (*p* < 0.001). Hemrová et al. (2019) found that fungal symbionts are a critical driver for the landscape distribution of *C. rubra* and other co-occurring forest orchid species. *Cephalanthera* have previously been shown to grow in a wide range of soil pH conditions, such as *C. damasonium* (pH 5.2–8.4) and *C. longifolia* (pH 3.3–7.8**)** (Azevedo, 2014).

In the current study, P ranged from 13 to 50 ppm for plants below pH 8 (*n* = 5), whilst plants in soil over pH 8 (*n* = 3), all had P contents of 13 ppm. The lower P concentrations and higher abundance of mycorrhizal fungi at Locations with high soil pH may reflect the need of the plant to associate with competent fungi, to increase its intake of P. It has been shown that both pH and P levels can affect the fungal communities in soil both in terms of the mycorrhizal fungi and the overall soil microbes (non-mycorrhizal fungi and bacteria), by altering P scavenging strategies (Carrino-Kyker et al., 2016). Alkaline soils can limit available P, due to interactions with poorly soluble calcium ions (Hoberg, Marschner & Lieberei, 2005; Hopkins & Ellsworth, 2005), unless an abundance of OM is present, which can interact with positively charged cations such as calcium (Ca^2+^) (Da Silva Cerozi & Fitzsimmons, 2016).

Further studies are critical to understand the link between culturable fungi and soil conditions using larger diverse collections from different sites. Only limited information is available about how abiotic factors influence mycorrhizal association in orchids (Phillips et al., 2011), but available evidence suggests that soil conditions affect fungal diversity (McCormick et al., 2012; Bunch et al., 2013). Mycorrhizal fungi are well known to increase P intake, with estimates as high as 100% of plant P being acquired directly from them (Smith, Smith & Jakobsen, 2003). In the present study, plants from soils with higher P content had lower levels or even absence of mycorrhizal fungi. However, Mujica et al. (2016) found that mycorrhizal associations are favoured by higher soil nutrient availability and suggested that orchid species in nutrient-poor soils may exhibit generalist associations. In our study mycorrhizal association increased in comparatively low P which agrees with the above finding. However, there was no significant difference in mycorrhizal association in relation to N and OM levels. Our finding also agrees with generalist behaviour reported in *Cephalanthera* spp. by Bidartondo & Read (2008) on seed germination and with Pecoraro et al. (2017) in roots of adult plants.

The endomycorrhizal fungi Ceratobasidiaceae and *Ceratobasidium* identified in this study have ITS sequences similar to other isolates obtained from both *Cephalanthera* sp. and other members of the Orchidaceae. Studies on French, German and Hungarian *C. damasonium* plants have revealed that they associate with a variety of basidiomycetes and ascomycetes (Bidartondo et al., 2004; Julou et al., 2005; Illyes et al., 2010). Italian *C. longifolia* have been shown to associate with *Hebeloma*, *Russula* and *Tomentella*, and Estonian *C. longifolia* with Thelephoraceae and *Sebacina* (Abadie et al., 2006; Liebel et al., 2010). These studies do, however, have small sample sizes and differences in collection sites and environments, making meaningful conclusions on whether the mycorrhizal specialisation of these plants is due to preference or a product of the environment hard to draw. Low level of specificity in both *C. longifolia* and *C. damasonium* has been reported before (Pecoraro et al., 2017). The current study shows that *C. rubra* also has little mycorrhizal specificity, with a mixture of both endomycorrhizal and EMF. The endomycorrhizal fungi recovered in this study were closely matched to the sequences of Ceratobasidiaceae, and *Ceratobasidium* sp. and members of these taxa are known to be typical orchid mycorrhizal fungi (Roy et al., 2009) with an ability to aid the germination of orchid dust seeds (Matsuda, Amiya & Ito, 2009).

Only five OTUs of mycorrhizal fungi were identified and some fungi could have been missed by using a culture-based method. This system was used as the aim was to isolate the compatible mycorrhizal fungi for seed germination and further establishment of seedlings in the wild. In the current study, all EMF were isolated from plants in the soil pH < 8 group and were identified as *Cenococcum geophilum* and *Tomentella. C. geophilum* is a common EMF important in soil biogeochemical cycles (McCormack et al., 2017) previously reported in *C. damasonium* (Pecoraro et al., 2017), whilst *Tomentella* have been found in closely related *C. falcata* (Thunb.) Blume and *C. erecta* (Thunb.) Blume (Matsuda, Amiya & Ito, 2009; Yagame & Yamato, 2013), whilst *C. austinae* (A. Gray) Heller have also been shown to associate with *Tomentella* (Taylor & Bruns, 1997). As *C. rubra* associates with beech and oak trees, as well as EMF, this suggests a tripartite relationship of the orchid, fungus and tree, as previously demonstrated in *C. falcata* (Yagame & Yamato, 2013). Indeed, some *Cephalanthera* species show dependency on EMF (Taylor & Bruns, 1997) with seedlings having higher mycorrhizal specificity than mature plants (Bidartondo & Read, 2008). This is an important consideration for conservation and forest management practices. Furthermore, Ceratobasidiaceae fungi have also been implicated in simultaneous associations with orchids as OMF and tree species as EMF (Yagame et al., 2012) suggesting the possibility of the indirect relationship of *C. rubra* with neighbouring tree species.

Ninety-six mycorrhizal and a range of non-mycorrhizal endophytes were isolated as part of this study underpinned by soil characteristics. Of these, the most interesting were *Cadophora*, *Cladophialophora*, *Exophiala*, *Cyphellophora* and *Phomopsis* sp. Some of these fungi identified in this study have previously been isolated from roots of *C. damasonium* (*Exophiala* and *Cadophora*) (Julou et al., 2005; Pecoraro et al., 2017). *Phomopsis* has been shown to increase N uptake (Yang et al., 2015), and the single *Phomopsis* isolate from this study was isolated from a root found in soil with very low N concentration (3 ppm). Moreover, an in vitro study has shown that *P. liquidambari* promote release of NH4^+^ from plant litter to soil, thereby increasing soil inorganic N (Chen et al., 2013). The presence of these fungi seen in plants growing in soil with a pH of <8, may be showing the ability of *C. rubra* plants to recruit non-mycorrhizal fungi when recruitment of mycorrhizal fungi is poor.

Neighbour-joining analysis suggests that the isolates in this study may be established associates of *Cephalanthera* species, and that *C. rubra* is not significantly different to other *Cephalanthera* species in its partnership with the fungal symbionts. This could also be helpful to identify the appropriate symbiont/s for seed germination of *C. rubra*.

*Cephalanthera* spp. are considered as generalists with respect to their relationship with mycorrhizal fungi, like many photosynthetic mycorrhizal plants, and obtain nutrients from their fungal partners to meet gaps in nutritional requirements when needed (Gebauer & Meyer, 2003; Bidartondo et al., 2004; Abadie et al., 2006; Bidartondo & Read, 2008; Smith & Read, 2008). Location D had the most vigorous plants, including Plant number 7, with the lowest soil P, N and OM levels. The root of Plant number 7 yielded a high number of *Ceratobasidium* isolates of a single OTU. Additional mycorrhizal fungi were also recovered amongst *C. rubra* in this study, which supports the above finding that *Cephalanthera* spp. recruit diverse mycorrhizal fungal partners.

Generalist orchids should be able to survive in a broader range of environmental conditions by switching between partner organisms, giving them a higher adaptivity to change (Swarts & Dixon, 2009). Mycorrhizal fungal specificity of *Cephalanthera* depended on developmental stage which shows that the fungal diversity of seedlings of *C. longifolia* and *C. damasonium* were narrower than that of mature plants that are associated with *Tomentella*, *Sebacina*, *Leptodontidium*, *Inocybe* and *Ceratobasidium* (Bidartondo & Read, 2008). Their study also found that, throughout the life cycle, the orchids formed associations with EMF from neighbouring tree roots, with seedlings showing a greater specificity for Thelephoraceae and Cortinariaceae.

The limited availability of study material that can be collected from extremely small populations of orchids always remains as an impediment to conduct research (Pecoraro et al., 2017). The present study was not an exception to this, with collection of only a small number of roots permitted from seedlings and mature plants. However, the available evidence from this study has shown that the oak woodland characteristics in France support successful and viable populations. Better woodland management at *C. rubra* sites in England to help boost environmental heterogeneity might help successful natural recruitment of seedlings and their maturation supported by a tripartite mycorrhizal system involving the orchid and tree. In vitro-raised symbiotic seedlings of *C. falcata* have already been shown to establish in a tripartite system ex situ (Yagame & Yamato, 2013). This could be an option worth pursuing in future for the conservation of the English Red Helleborine. As five mycorrhizal OTUs are available following this study, further investigations are underway to determine whether they can help to germinate *C. rubra* in vitro to support the long-term objective of reintroducing symbiotic planting stock back to the wild in England.

## Conclusions

Understanding the distribution and abundance of mycorrhizal and other root-associated fungi from plants of high conservation value with extremely small populations is challenging. Use of root samples from plants at different maturity offers potential to identify diverse fungi for time-bound research projects although the full suite of fungi may not be available. This study highlights the need to understand the culturable fungi of a threatened orchid which depends both on endo and EMF for successful establishment of populations. A total of 468 fungal isolates were obtained from roots of *C. rubra*, representing 96 operational taxonomic units. Of these, five were mycorrhizal, representing three endomycorrhizal and two EMF. Both soil pH and P were shown to have statistically significant effects on fungal diversity, with mycorrhizal fungi being present in greater numbers at comparatively low P and higher pH levels. At relatively lower pH levels, a greater number of non-mycorrhizal fungi were found. Further collecting from other European countries during different seasons collecting protocorms, seedlings and mature plants at different growth stages is essential to identify a wider selection of culturable mycorrhizal fungi. Work is currently underway to collect, identify and test available mycorrhizal fungi to develop methods to germinate seeds of *C. rubra* available from different European sources. This might eventually help identify compatible fungi for English populations of *C. rubra* that are on the brink of extinction.

## Supplemental Information

10.7717/peerj.8695/supp-1 Supplemental Information 1 Recovered fungi from plants in soil with pH of >8.

10.7717/peerj.8695/supp-2 Supplemental Information 2 Recovered fungi from plants in soil with pH of <8.

10.7717/peerj.8695/supp-3 Supplemental Information 3 *Ceratobasidium* phylogeny tree. Neighbour-Joining phylogeny tree of aligned sequences of the three endomycorrhizal fungi isolated from *Cephalanthera rubra*, collected from Loire Valley, France. Also included are close BLAST matches in GenBank for each OTU for which host and location information were available, as well as representative sequences of published orchid endomycorrhizal symbionts. The tree was rooted using a *Ceratobasidium ramicola* sequence and bootstrap percentages >50% are shown after 1,000 replications.

10.7717/peerj.8695/supp-4 Supplemental Information 4 *Tomentella* phylogeny tree.

10.7717/peerj.8695/supp-5 Supplemental Information 5 *Cenococcum* phylogeny tree.

10.7717/peerj.8695/supp-6 Supplemental Information 6 Raw data.

10.7717/peerj.8695/supp-7 Supplemental Information 7 DNA sequences and accession details.

## Additional Information and Declarations

### Field Study Permissions

The following information was supplied relating to field study approvals (i.e. approving body and any reference numbers): The Direction Departmentale des Territoires, D’lndre-et-Loire approved field collections. Mr. and Mrs. Bernard, the landowners, permitted us to collect the materials.

### DNA Deposition

The following information was supplied regarding the deposition of DNA sequences: DNA sequences are available at GenBank: MN450567–MN450662.

### Data Availability

The following information was supplied regarding data availability: Raw data is available in the Supplemental Files.
