# Supplementary material for: Diversity of root-associated culturable fungi of Cephalanthera rubra (Orchidaceae) in relation to soil characteristics
Source: PeerJ. 2020 Mar 2;8:e8695. doi: 10.7717/peerj.8695 (PMC7058101; doi:10.7717/peerj.8695)
Supplement: Supplemental Information 1 [file peerj-08-8695-s001.docx]

**Supplemental information 1:** Recovered fungi from plants in soil with pH of >8

| Fungi | GenBank match ID (≥97%) | Plant number (Location) | Count | Total count for genus/species | Percentage of total |
| --- | --- | --- | --- | --- | --- |
| *Cadophora* sp. | KX610422 | 7 (D) | 3 | 19 | 20.4 |
| *Cadophora* sp. | KX610420 | 4, 7 (B, D) | 16 |  |  |
| *Hypocreales* sp. | KC007311 | 7 (D) | 1 | 3 | 3.2 |
| *Hypocreales* sp. | KC007264 | 7 (D) | 2 |  |  |
| *Virgaria nigra* | AB670714 | 6,7 (D) | 4 | 4 | 4.3 |
| *Penicillium cineroatrum* | NR144837 | 7 (D) | 1 | 1 | 1.1 |
| *Dactylonectria alcacerensis* | NR121498 | 4 (B) | 1 | 1 | 1.1 |
| *Tetracladium* sp. | GU327473 | 4 (B) | 1 | 2 | 2.2 |
| *Tetracladium* sp. | EU516790 | 4 (B) | 1 |  |  |
| Pleosporales ap. | KC180719 | 4, 6 (B, D) | 4 | 4 | 4.3 |
| *Paecilomyces* *carneus* | FN394726 | 7 (D) | 1 | 1 | 1.1 |
| *Ceratobasidium* sp. | KJ188567 | 6, 7 (D) | 54 | 54 | 58.1 |
| *Clonostachys rosea* | KJ619987 | 4 (B) | 1 | 1 | 1.1 |
| Unidentified root fungus | KX791021 | 4, 6 (B, D) | 2 | 2 | 2.2 |
| *Eladia saccula* | KJ028785 | 7 (D) | 1 | 1 | 1.1 |
