# Supplementary material for: Diversity of root-associated culturable fungi of Cephalanthera rubra (Orchidaceae) in relation to soil characteristics
Source: PeerJ. 2020 Mar 2;8:e8695. doi: 10.7717/peerj.8695 (PMC7058101; doi:10.7717/peerj.8695)
Supplement: Supplemental Information 2 [file peerj-08-8695-s002.docx]

**Supplemental information 2:** Recovered fungi from plants in soil with pH of <8

| Fungi | GenBank match ID (≥97%) | Plant number | Count | Total count for genus/species | Percentage of total |
| --- | --- | --- | --- | --- | --- |
| *Cadophora* sp. | KX610422 | 1, 2, 3 (A) | 14 | 55 | 14.7 |
| *Cadophora* sp. | KX610420 | 1, 2, 3, 5, 8 (A, C, D) | 40 |  |  |
| *Cadophora malorum* | GU067760 | 2 (A) | 1 |  |  |
| *Hypocreales* sp. | KC007311 | 1, 2, 5 (A, C) | 46 | 47 | 12.5 |
| *Hypocreales* sp. | FR865004 | 5 (C) | 1 |  |  |
| *Acremonium* sp. | KJ170312 | 1, 2, 5 (A, C) | 22 | 32 | 8.5 |
| *Acremonium* sp. | EU139249 | 1, 5 (A, C) | 10 |  |  |
| *Talaromyces amestolkiae* | NR_120179 | 1 (A) | 1 | 1 | 0.3 |
| *Saccharicola bicolor* | KT3675256 | 1 (A) | 1 | 1 | 0.3 |
| Ceratobasidiaceae | DQ182460 | 1 (A) | 2 | 2 | 0.5 |
| *Nemania serpens* | KU141386 | 1 (A) | 1 | 1 | 0.3 |
| *Virgaria nigra* | AB670714 | 1, 2, 3, 5, 8 (A, C, D) | 17 | 17 | 4.5 |
| *Cenococcum geophilum* | KC967384 | 1 (A) | 2 | 2 | 0.5 |
| *Ilyonectria* sp. | KX640659 | 1, 2, 3, 5 (A, C) | 13 | 26 | 6.9 |
| *Ilyonectria* sp. | KT269540 | 2, 5 (A, C) | 4 |  |  |
| *Ilyonectria robusta* | KJ541686 | 2 (A) | 1 |  |  |
| *Ilyonectria* sp. | KX610383 | 5 (C) | 4 |  |  |
| *Ilyonectria* sp. | KT264484 | 5 (C) | 3 |  |  |
| *Ilyonectria* sp. | KT268752 | 5 (C) | 1 |  |  |
| *Penicillium murcianum* | NR_138358 | 1, 5 (A, C) | 4 | 50 | 13.3 |
| *Penicillium cineroatrum* | NR144837 | 2, 5, 8 (A, C, D) | 7 |  |  |
| *Penicillium janthinellum* | DQ888733 | 2, 3 (A) | 21 |  |  |
| *Penicillium pancocmium* | JN617678 | 2, 5 A, C) | 3 |  |  |
| *Penicillium* sp. | LC109284 | 2, 3 (A) | 3 |  |  |
| *Penicillium swiecickii* | NR121254 | 3 (A) | 1 |  |  |
| *Penicillium bilaiae* | NR_111679 | 3 (A) | 1 |  |  |
| *Penicillium carminoviolaceum* | KC411728 | 3, 5 (A, C) | 3 |  |  |
| *Penicillium atramentosum* | GU188272 | 5 (C) | 2 |  |  |
| *Penicillium* sp. | KX148639 | 5 (C) | 1 |  |  |
| *Penicillium fellutanum* | EU816399 | 5 (C) | 1 |  |  |
| *Penicillium* sp. | AM261634 | 8 (D) | 1 |  |  |
| *Penicillium restrictum* | JQ346224 | 8 (D) | 1 |  |  |
| *Penicillium* sp. | KP403964 | 8 (D) | 1 |  |  |
| *Ascotricha erinacea* | KT224876 | 1 (A) | 1 | 1 | 0.3 |
| *Cladophialophora* sp. | AB986416 | 1, 8 (A, D) | 2 | 2 | 0.5 |
| *Helotiales* sp. | KX610429 | 2 (A) | 2 | 2 | 0.5 |
| *Ochroconis globalis* | KF961085 | 2 (A) | 4 | 4 | 1.1 |
| *Thielaviopsis basicola* | KJ956786 | 2 (A) | 2 | 2 | 0.5 |
| *Cordana pauciseptata* | HE672148 | 2 (A) | 1 | 1 | 0.3 |
| *Helotiaceae* sp. | KX610432 | 2 (A) | 1 | 1 | 0.3 |
| *Cordyceps* sp. | EU139248 | 2 (A) | 1 | 1 | 0.3 |
| *Mortierella alpina* | KP714627 | 2, 5 (A, C) | 3 | 6 | 1.6 |
| *Mortierella* sp. | GU327527 | 2 (A) | 1 |  |  |
| *Mortierella* sp. | KC018255 | 1 (A) | 1 |  |  |
| *Dactylonectria alcacerensis* | NR121498 | 2, 3, 5 (A, C) | 6 | 6 | 1.6 |
| *Exophiala* sp. | KX610455 | 3 (A) | 1 | 7 | 1.9 |
| *Exophiala equina* | KU705835 | 5 (C) | 4 |  |  |
| *Exophiala* sp. | JX625264 | 5 (C) | 2 |  |  |
| *Tetracladium* sp. | GU327473 | 5 (C) | 1 | 5 | 1.3 |
| *Tetracladium* sp. | EU516790 | 3, 5 (A, C) | 3 |  |  |
| *Tetracladium* sp. | GU395525 | 5 (C) | 1 |  |  |
| *Pleosporales* sp. | KC180719 | 3 (A) | 2 | 5 | 1.3 |
| *Pleosporales* sp. | KF428668 | 3 (A) | 2 |  |  |
| *Pleosporales* sp. | KC871044 | 5 (C) | 1 |  |  |
| *Melanoconium hedericola* | NR137940 | 3 (A) | 14 | 14 | 3.7 |
| *Paecilomyces carneus* | FN394726 | 3 (A) | 1 | 9 | 2.4 |
| *Paecilmyces* sp. | KF428241 | 5 (C) | 8 |  |  |
| *Ascovitgaria occulta* | AB740957 | 2 (A) | 1 | 1 | 0.3 |
| *Pyrenochaeta* sp. | KX610438 | 3 (A) | 2 | 2 | 0.5 |
| *Cyphellophora* sp. | KT269650 | 5 (A) | 4 | 7 | 1.9 |
| *Chyphellophora* sp. | KT268871 | 3 (A) | 3 |  |  |
| *Dothideomycetes* sp. | KX440180 | 3 (A) | 1 | 1 | 0.3 |
| *Ceratobasidium* *albasitensis* | KX610453 | 3 (A) | 2 | 2 | 0.5 |
| *Hypocrea koningii* | X93984 | 3, 5 (A, C) | 2 | 3 | 0.8 |
| *Hypocrea lixii* | FJ442267 | 5 (C) | 1 |  |  |
| *Fusarium merismoides* | KU214553 | 5 (C) | 1 | 1 | 0.3 |
| Unidentified root fungus | EU917113 | 5 (C) | 1 | 6 | 1.6 |
| Unidentified root fungus | JX381454 | 5 (C) | 4 |  |  |
| Unidentified root fungus | EF434063 | 5 (C) | 1 |  |  |
| *Phomopsis* sp. | KX610406 | 5 (C) | 3 | 3 | 0.8 |
| *Trichoderma* sp. | KM520358 | 5 (C) | 3 | 5 | 1.3 |
| *Trichoderma* sp. | LC229679 | 5 (C) | 2 |  |  |
| Mycohaetophora | HG936157 | 5 (C) | 1 | 1 | 0.3 |
| *Collarina aurantiaca* | KJ807178 | 5 (C) | 8 | 8 | 2.1 |
| Ascomycota sp. | KP714640 | 5 (C) | 1 | 3 | 0.8 |
| Ascomycota sp. | LC229662 | 2 (A) | 2 |  |  |
| *Neonectria radicola* | GQ922913 | 5 (C) | 1 | 1 | 0.3 |
| *Trichosporon* sp. | JX675205 | 5 (C) | 2 | 2 | 0.5 |
| *Oideiodendron flavum* | KJ921607 | 5 (C) | 6 | 6 | 1.6 |
| *Chrysosporium keratinophilum* | AB361657 | 5 (C) | 3 | 3 | 0.8 |
| *Humicola* sp. | KX610414 | 5 (C) | 1 | 1 | 0.3 |
| *Eladia saccula* | KJ028785 | 5, 8 (C, D) | 3 | 3 | 0.8 |
| *Apiotrichum dulcitum* | NR073248 | 5 (C) | 2 | 2 | 0.5 |
| *Wardomyces dimerus* | LN850991 | 5 (C) | 1 | 1 | 0.3 |
| Trichomaceae sp. | KX148652 | 5 (C) | 1 | 1 | 0.3 |
| *Doramtomyces* sp. | LT623991 | 5 (C) | 1 | 1 | 0.3 |
| *Cosmospora* sp. | JX145394 | 5 (C) | 3 | 3 | 0.8 |
| *Mucor hiemalis* | LN714573 | 5 (C) | 1 | 1 | 0.3 |
| *Hypholoma fasciculare* | LN901110 | 8 (D) | 1 | 1 | 0.3 |
| *Hypoxylon serpens* | AY805565 | 8 (D) | 1 | 1 | 0.3 |
| *Tomentella* sp. | JX625354 | 8 (D) | 2 | 2 | 0.5 |
| *Sordariomycetes* sp. | KP991552 | 2 (A) | 3 | 3 | 0.8 |
