# Supplementary figures and images for: Diversity of root-associated culturable fungi of Cephalanthera rubra (Orchidaceae) in relation to soil characteristics
Source: PeerJ. 2020 Mar 2;8:e8695. doi: 10.7717/peerj.8695 (PMC7058101; doi:10.7717/peerj.8695)

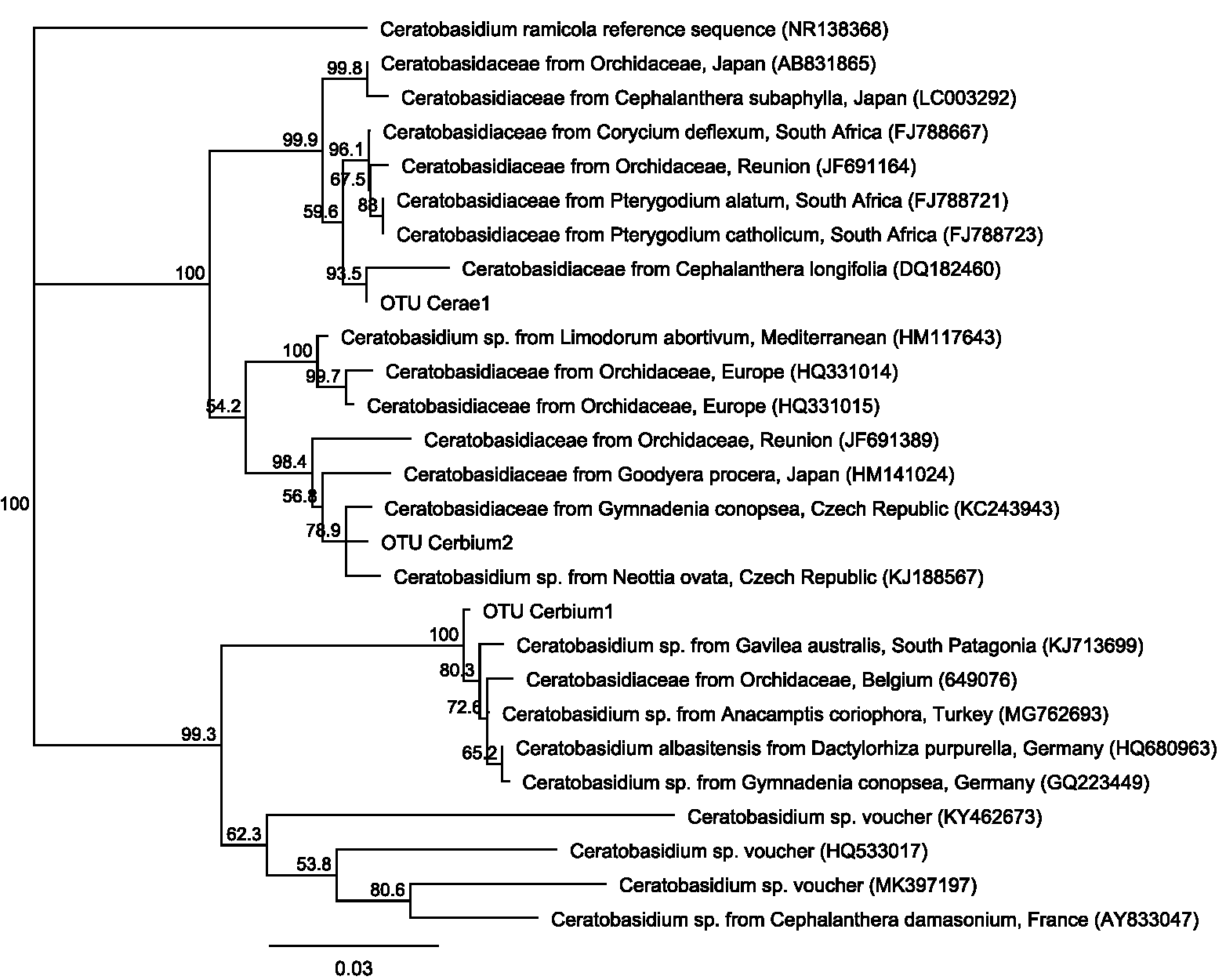

Supplement: Supplemental Information 3 — Neighbour-Joining phylogeny tree of aligned sequences of the three endomycorrhizal fungi isolated from Cephalanthera rubra, collected from Loire Valley, France. Also included are close BLAST matches in GenBank for each OTU for which host and location information were available, as well as representative sequences of published orchid endomycorrhizal symbionts. The tree was rooted using a Ceratobasidium ramicola sequence and bootstrap percentages >50% are shown after 1,000 replications. [file peerj-08-8695-s003.png]

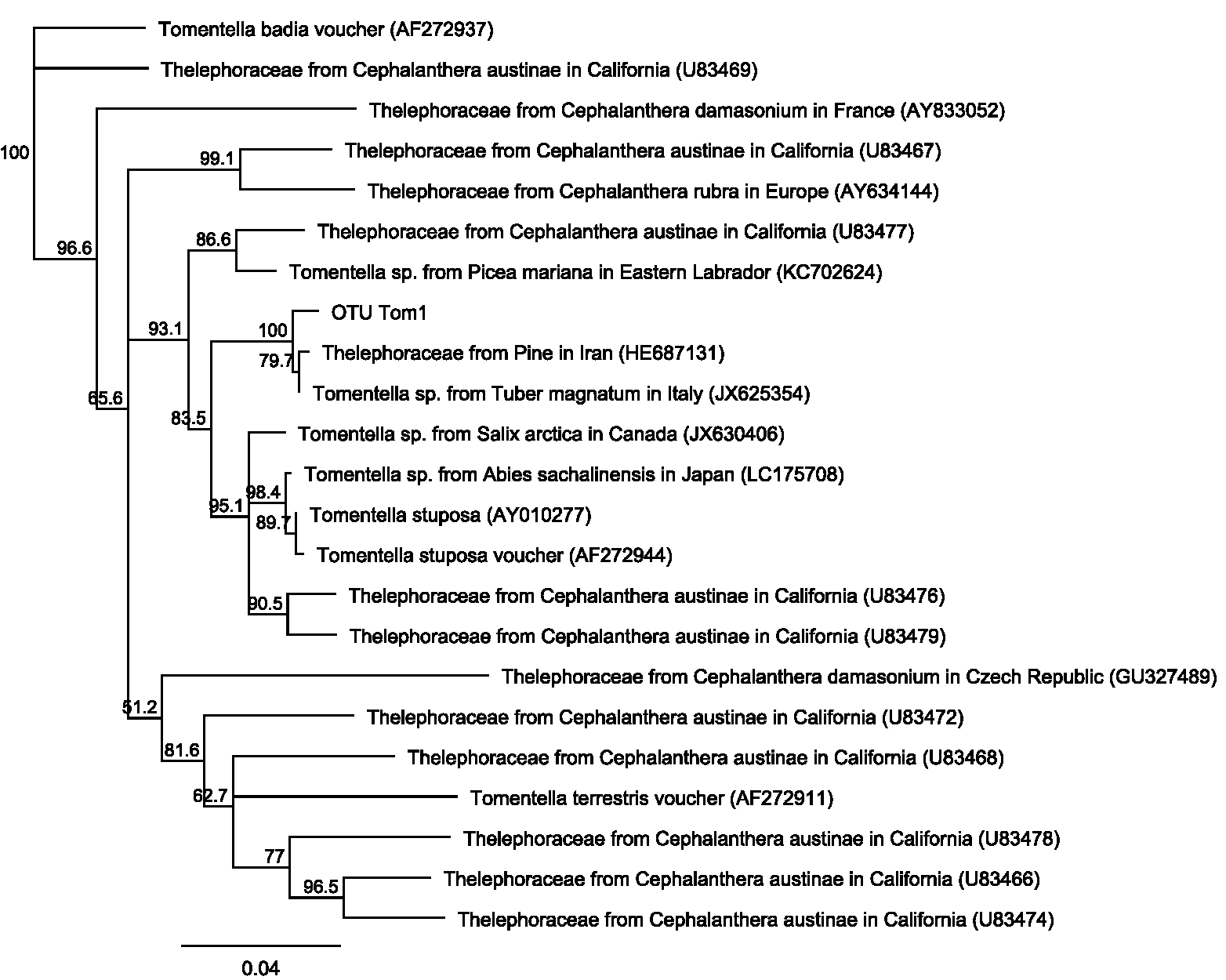

Supplement: Supplemental Information 4 [file peerj-08-8695-s004.png]

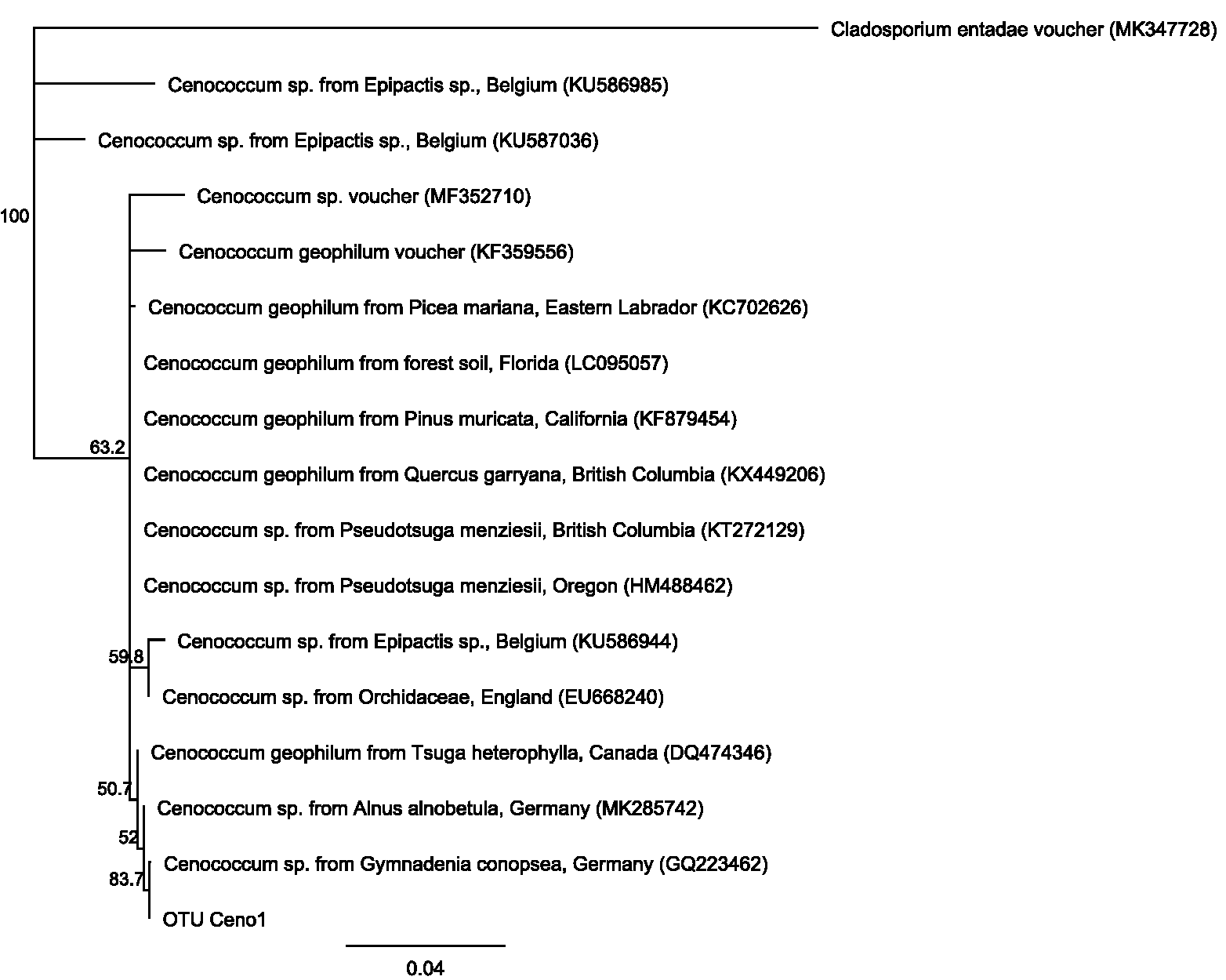

Supplement: Supplemental Information 5 [file peerj-08-8695-s005.png]
