# Supplementary material for: Diversity of root-associated culturable fungi of Cephalanthera rubra (Orchidaceae) in relation to soil characteristics
Source: PeerJ. 2020 Mar 2;8:e8695. doi: 10.7717/peerj.8695 (PMC7058101; doi:10.7717/peerj.8695)
Supplement: Supplemental Information 7 [file peerj-08-8695-s007.docx]

**Sequence data to GenBank, accession numbers of the nucleotide sequences and sequences (Sequence details in bold are that of mycorrhizal fungi)**

SUB6288079 Seq1         MN450567

>Seq1 [organism=Cadophora sp.] [Cad1] 18S ribosomal RNA gene, partial sequence; internal transcribed spacer 1, 5.8S ribosomal RNA gene, and internal transcribed spacer 2, complete sequence; and 28S ribosomal RNA gene, partial sequence

AGACCTACCGGACTCAATCGCGAGGAGTATTACTACGCGTAGAGCFCGACAGGCACCGCCACTGATTTTAGGGGCCGCGGAACCGCGAGCCCCAATACCAAGCGAGAGCTTGAGTGGTTATAATGACGCTCGAACAGGCATGCCCCCCGGAATACCAGAGGGCGCAATGTGCGTTCAAAGATTCGATGATTCACTGAATTCTGCAATTCACATTACTTATCGCATTTCGCTGCGTTCTTCATCGATGCCAGAACCAAGAGATCCGTTGTTGAAAGTTTTAACTATTATATAGTACTCAGACATCACAAAAAACAAGAGTTGTGGTCCTCTGGCGGGCACTCAACAGCCGAAGCCGCTGGCACGAGGCGGCCCGCCAAAGCAACAAAGGTAATTTATTCAAGGGTGGAGTTCAGGACCGAACTTCTCCGAGAGGTTCGACGACTCTAAACCCTACCGAAGTAGGGTAGCCCGCCACGAAGCAAGCTTCTGTGGGGCGCTGCCTATCCTTTGCTCTAGTAATGATCCTTCCGCAGGTTCACCCTACGGAAGA

SUB6288079 Seq2         MN450568

>Seq2 [organsim=Cadophora sp.] [Cad2] 18S ribosomal RNA gene, partial sequence; internal transcribed spacer 1, 5.8S ribosomal RNA gene, and internal transcribed spacer 2, complete sequence; and 28S ribosomal RNA gene, partial sequence

AAGGATCATTACTAGAGCAAAGGATAGGCAGCGCCCCACAGAAGCTTGCTTCGTGGCGGGCTACCCTACTTCGGTAGGGTTTAGAGTCGTCGAACCTCTCGGAGAAGTTCGGTCCTGAACTCCACCCTTGAATAAATTACCTTTGTTGCTTTGGCGGGCCGCCTCGTGCCAGCGGCTTCGGCTGTTGAGTGCCCGCCAGAGGACCACAACTCTTGTTTTTTGTGATGTCTGAGTACTATATAATAGTTAAAACTTTCAACAACGGATCTCTTGGTTCTGGCATCGATGAAGAACGCAGCGAAATGCGATAAGTAATGTGAATTGCAGAATTCAGTGAATCATCGAATCTTTGAACGCACATTGCGCCCTCTGGTATTCCGGGGGGCATGCCTGTTCGAGCGTCATTATAACCACTCAAGCTCTCGCTTGGTATTGGGGCTCGCGGTTCCGCGGCCCCTAAAATCAGTGGCGGTGCCTGTCGGCTCTACGCGTAGTAATACTCCTCGCGATTGAGTCCGGTAGGTCTACTTGCCAGCAACCCCCAATTTTACAGGTTGACCTCGATCAGGTAGGGATACCCGCTGAACTTAA

SUB6288079 Seq3         MN450569

>Seq3 [organism=Cadophora malorum] [Cad3] 18S ribosomal RNA gene, partial sequence; internal transcribed spacer 1, 5.8S ribosomal RNA gene, and internal transcribed spacer 2, complete sequence; and 28S ribosomal RNA gene, partial sequence

AAGGATCATTAATAAACCTAGTCGTTCGCGTACATAGGGGCAAACCCTCGCGTGATGTCAGCAATACCTAGGTAACCCTTGAATAAACTACCCTTGTTGCTTTGGCGGGTCGCCTTTCAGGCCAGCGGCTTCGGCTGCTGCGTGCCCGCCAGAGGACCACAACTCTTGTTTTTAGTGATGTCTGAGTACTATATAATAGTTAAAACTTTCAACAACGGATCTCTTGGTTCTGGCATCGATGAAGAACGCAGCGAAATGCGATAAGTAATGTGAATTGCAGAATTCAGTGAATCATCGAATCTTTGAACGCACATTGCGCCCTTTGGTATTCCGGAAGGGCATGCCTGTTCAGCGTCATTATAACCACTAAGCCTTCGCTTGGTATTGGGGTTCGCGGTTCCGCGGCCTCTAAAATCAGTGGCGGTGCCTGTCGGCTCTCCGTAGTAATACTCCTCCGTCTGGGTCCGGTAGTTGCTTGCCAACAACCCCAAATTTTTTACAGGTGACCTCGCATCAGTAGGGATACCCCTGACTTAA

SUB6288079 Seq4         MN450570

>Seq4 [organism=Hypocreales sp.] [Hypo1] 18S ribosomal RNA gene, partial sequence; internal transcribed spacer 1, 5.8S ribosomal RNA gene, and internal transcribed spacer 2, complete sequence; and 28S ribosomal RNA gene, partial sequence

AAAAGTCGTAACAAGGTCTCCGTTGGTGAACCAGCGGAGGGATCATTACAGAGTCTCCGAACTCCAAACCTTTGTGAACATACCACCGTTGCTTCGGCGGGACCCCCCCGGGGCGCGCAGTGCGCTGCACGCCCCGGGCGCCCGCCGAGGGCCCCAAACCATTTCTCCATAGTGGACTCTTCAGAGTTTGATACACAATCAATCAAAACTTTCAGCAACGGATCTCTTGGCTCTGGCATCGATGAAGAACGCAGCAAAATGCGATAAGTAATGTGAATTGCAGAATTCAGTGAATCATCGAATCTTTGAACGCACATTGCGCCCGCCAGCATCCTGGCGGGCATGCCTGTCCGAGCGTCATTTCAACCCTCAGGGCCCGTCCGCGGGACCTGGCGTTGGGGACCGGCCGCGCCCACGAGGCCGGCCGGCCCTGAAATACAGTGGCGGTCACGTCGCGACCTCCCCCGCGTAGTAGAACTCACCTCGCGGCGGGAAGGCGGCGCGGCCACGCCGTGAAACCCCCTACTTCTCAAGGTTGACCTCGGATCAGGTAGGAATACCCGCTGAACTTAA

SUB6288079 Seq5         MN450571

>Seq5 [organism=Hypocreales sp.] [Hypo2] 18S ribosomal RNA gene, partial sequence; internal transcribed spacer 1, 5.8S ribosomal RNA gene, and internal transcribed spacer 2, complete sequence; and 28S ribosomal RNA gene, partial sequence

CGTAGGATATTACTGAGTTTACACTCCCAACCCTTTGTGAACCTTACCTATCGTTGCTTCGGCGGACCGTCCCGGGTGCTGCGTGCCCCGGACCCAGGCGCCCGCCCGGGGACCACTCAAACCCTGTTTTTGTTTTTTTCAAAACATGTGTATCTTCTGAGCGAGCCGAAAGGCGTCAAAAAACAATCAAAACTTTCAACAACGGATCTCTTGGTTCTGGCATCGATGAAGAACGCAGCGAAATGCGATAAGTAATGTGAATTGCAGAATTCAGTGAATCATCGAATCTTTGAACGCACATTGCGCCCGCCAGTATTCTGGCGGGCATGCCTGTCCGAGCGTCATTTCAACCCTCGGGCCCCCCTCTGCGGGCGGCCCGGCGTTGGGGCTCAGGCGCCGTCTCTGCTCGGCGCCTGTCTCCTAAATGCAGTGGCGGCCTCGCCGCTGCCTCCTCCGCGTAGTAGCACAAACCTCGCGGCTGGAAAGCGGCGCGGCCACGC

SUB6288079 Seq6         MN450572

>Seq6 [organism=Hypocreales sp.] [Hypo3] 18S ribosomal RNA gene, partial sequence; internal transcribed spacer 1, 5.8S ribosomal RNA gene, and internal transcribed spacer 2, complete sequence; and 28S ribosomal RNA gene, partial sequence

AAACCCTTTGTGAACACTACCTTCGTTGCTTCGGCGGGACCGCCCCGGCGCCTCTGCGCGCCGGAACCAGGCGCCCGCCGGGGGCCCGAAACTCTTGTTTTGTCAGTGGTATTTTCTCTGAGTGGCATAAGCAAATAAATTAAAACTTTCAACAACGGATCTCTTGGTTCTGGCATCGATGAAGAACGCAGCGAAATGCGATAAGTAATGTGAATTGCAGAATTCAGTGAATCATCGAATCTTTGAACGCACATTGCGCCCGCCAGTACTCTGGCGGGCATGCCTGTTCGAGCGTCATTCAACCCTCGGGCCCCGCATTCGTGCGGGCCCCGGCGTTGGGGACCGGCCTCCGCATCGGACGGCCGCCCCCGAAACCCAGTGGCGGTCCCGCCGCGGCCTTCTCTGCGTAGTAGCATACACCTCGCACTGGAGAGCGGCGCGGCCACGCCGTAAAAGAAACGAACTTTCTGAAGGTTGACCTCGGATCAGGTAGGAATACCCGCTGAACTTAAGCATATCAATAAGCGGAGGAA

SUB6288079 Seq7         MN450573

>Seq7 [organism=Acremonium sp.] [Acrem1] 18S ribosomal RNA gene, partial sequence; internal transcribed spacer 1, 5.8S ribosomal RNA gene, and internal transcribed spacer 2, complete sequence; and 28S ribosomal RNA gene, partial sequence

AGGGATCATTACTGAGTGTAAAAACTCCCAAACCCCTGTGAACATACCATCCGTTGCTTCGGCGGGATCGCCCCGGGCGCCTTTGCGTGCCCCGGATCCAGGCGCCCGCCGGAGGACTCCAAACTCTTGTTTTATATGTGGCATTATCTGAGTGGCTTATAGCAAAATAAATCAAAACTTTCAACAATGGATCTCTTGGCTCTGGCATCGATGAAGAACGCAGCGAAATGCGATAAGTAATGCGAATTGCAGAATTCAGTGAATCATCGAATCTTTGAACGCACATTGCGCCCGCCAGTATTCTGGCGGGCATGCCTGTCTGAGCGTCAGTTCAACCCTCGCAACCGGCTTTGCTGGATGTGGTGTTGGGGATCGGCTGCCTTCTAGGTGGCCGGCCCCGAAATAGAGTGGCGGTCTCGTCGTGGCCTCCTCTGCGTAGTAGCAATATCTCGCAGGCGGAGAGCGGCGTGGCTATGCCGTAAAACACCCAACTCTTCTAAGGTTGACCTCAGATCAGGTAGGAATACCCGCTGAACTTAA

SUB6288079 Seq8         MN450574

>Seq8 [organism=Acremonium sp.] [Acrem2] 18S ribosomal RNA gene, partial sequence; internal transcribed spacer 1, 5.8S ribosomal RNA gene, and internal transcribed spacer 2, complete sequence; and 28S ribosomal RNA gene, partial sequence

AGGGATCATTACTGAGTGTAAAAACTCCCAAACCCCTGTGAACATACCATCTGTTGCTTCGGCGGGATCGCCCCGGGCGCCTTTGCGTGCCCCGGATCCAGGCGCCCGCCGGAGGACTCCAAACTCTTGTTTTTATATGTGGCATTATCTGAGTGGCTTATAGCAAAATAAATCAAAACTTTCAACAATGGATCTCTTGGCTCTGGCATCGATGAAGAACGCAGCGAAATGCGATAAGTAATGCGAATTGCAGAATTCAGTGAATCATCGAATCTTTGAACGCACATTGCGCCCGCCAGTATTCTGGCGGGCATGCCTGTCTGAGCGTCAGTTCAACCCTCACAACCGGCTTTCTTGCTGGATGTGGTGTTGGGGATCGGCTGCCTTTCTTCTAGGTGGCCGGCCCCGAAATAGAGTGGCGGTCTCGTCGTGGCCTCCTCTGCGTAGTAGCAATATCTCGCAGGCGGAGAGCGGCGTGGCTATGCCGTAAAACACCCAACTCTTCTAAGGTTGACCTCAGATCAGGTAGGAATACCCGCTGAACTTAA

SUB6288079 Seq9         MN450575

>Seq9 [organism=Talaromyces amestolkiae] [Tala1] 18S ribosomal RNA gene, partial sequence; internal transcribed spacer 1, 5.8S ribosomal RNA gene, and internal transcribed spacer 2, complete sequence; and 28S ribosomal RNA gene, partial sequence

GGGTAACTCCTACCTGATCCGAGGTCAACCGTGGTAAATTTTGGTGGTGACCAACCCCCGCCAGTCCTTCCCGAGCGAGTGACAAAGCCCCATACGCTCGAGGACCAGACGGACGTCGCCGCTGCCTTTCGGGCAGGTCCCCGGGGGGACCGCACCCAACACACAAGCCGTGCTTGAGGGCAGAAATGACGCTCGGACAGGCATGCCCCCCGGAATGCCAGGGGGCGCAATGTGCGTTCAAAGATTCGATGATTCACGGAATTCTGCAATTCACATTACTTATCGCATTTCGCTGCGTTCTTCATCGATGCCGGAACCAAGAGATCCATTGTTGAAAGTTTTGACAATTTTCATAGTACTCAGACAGCCCATCTTCATCAGGGTTCACAGAGCGCTTCGGCGGGCGCGGGCCCGGGGACAGATGTCCCCCGGCGACCAGGTGGCCCCGGTGGGCCCGCCAAAGCAACAGGTGTATAGAGACAAGGGTGGGAGGTTGGGCCACGAGG

SUB6288079 Seq10        MN450576

>Seq10 [organism=Saccharicolabicolor] [Sacc1] 18S ribosomal RNA gene, partial sequence; internal transcribed spacer 1, 5.8S ribosomal RNA gene, and internal transcribed spacer 2, complete sequence; and 28S ribosomal RNA gene, partial sequence

CAGCGGGTATCCCTACCTGATCCGAGGTCAAGTCATAAAAGAGCTTACTGGACGTTGATCCACCAAACTTCAAGAAACGCAAATGTGCTGCGCGAGAAGCTGGCACGACCGCTGCCAATGACTTTGGGGCGAGTCCACGCATAAAGCGGGACAGACGCCCAACACCAAGCAGAGCTTGAGGGTGTAAATGACGCTCGAACAGGCATGCCCCACGGAATACCGAGGGGCGCAATGTGCGTTCAAAGATTCGATGATTCACGGAATTCTGCAATTCACACTACTTTTCGCATTTCGCTGCGTTCTTCATCGATGCCAGAACCAAGAGATCCATTGTTGAAAGTTGTAATAATTTAAATAGTTATCAGAGATTTTAATACTGTAAATACAAGAGTTTAAGGGGTTCCTAGCGGCAGGCAAGCCTGCCGAGGAAACAACAAGAGGTACACGTAGACAAAGGGTGGATGCAATAGCCCCCGAAGAGCTATAGCTTTCGTAATTAATGATCCTTCC

**SUB6288079 Seq11        MN450577**

**>Seq11 [organism=Ceratobasidiaceae] [Cerae1] 18S ribosomal RNA gene, partial sequence; internal transcribed spacer 1, 5.8S ribosomal RNA gene, and internal transcribed spacer 2, complete sequence; and 28S ribosomal RNA gene, partial sequence**

**TTTGGTTATAGAGAGTGTCCTCAGCGATTAAATTAACTTATCACGCCAAGTGGAACCAAGCATAACACTGAGAGAATCCAGCTAATGCACAAAGAGGAGCAGGTGTGAAGCTGCAATAAACCTCCAATACCAAAGTAGAACCAATTGAGTGAACAAAAGATTTACTTTGAAGATTTCACGATACTCAAACAGGCATGCTCCAAGGAATACCGAGGAGCGCAAGGTGCGTTCAAAGATTCGATGATTCACTGAATTTGCAATTCACATTACTTATCGCATTTCGCTGCGTTCTTCATCGATGCGAGAGCCAAGAGATCCGTTGTTGAAACTTAGTATTAGATGTGTTACATCCATTACATTCATTTTAAAATAAATTGAGTTTATATAAAATTAAATAGACAGCAGGGTCCCCCATCAAAAAAAAAATGAAAGTTCCCCTAAATCTGTCTCACAGGTGCACAGGTGTGTATGGATAAAAGAGAAAGGCGTGCACATGCCTGAATTAAAATTTAATTCAAAAAATCATTA**

SUB6288079 Seq12        MN450578
>Seq12 [organism=Nemania serpens] [Nerm1] 18S ribosomal RNA gene, partial sequence; internal transcribed spacer 1, 5.8S ribosomal RNA gene, and internal transcribed spacer 2, complete sequence; and 28S ribosomal RNA gene, partial sequence

TATTGATATGCTTAAGTTCAGCGGGTATTCCTACCTGATCCGAGGTCAACCTATAAAAATTGGGGTGTTTTACGGCAGGGGACCGGCCCGCGCTACAGGCGACGTGTAAAAGCTACTACGTCTGGAGTGCGAACCGGCTCCGCCACTGACTTTGGGGAGCTACCAGAGAGTTCCGGTAGGCTCCTAACGCTAAGCAACAGGGGCTTAAGGGTTGAAATGACGCTCGAACAGGCATGCCCACCAGAATACTAGTGGGCGCAATGTGCGTTCAAAGATTCGATGATTCACTGAATTCTGCAATTCACATTACTTATCGCATTTCGCTGCGTTCTTCATCGATGCCAGAACCAAGAGATCCGTTGTTGAAAGTTTTAACTTATTTAGTTATATTTTCAGAAGTTTAATGCTATAAAACAGAGTTTCGGGGGCCGTCGGCAGGGTCGTCTACCGGGTAAGGTCCTACAGGGTAGGTTCTTACGGGGCGAGACGCCACCTGCCGAGGCAACGCGAGGTATGTTC

SUB6288079 Seq13        MN450579
>Seq13 [organism=Vigaria nigra] [Vini1] 18S ribosomal RNA gene, partial sequence; internal transcribed spacer 1, 5.8S ribosomal RNA gene, and internal transcribed spacer 2, complete sequence; and 28S ribosomal RNA gene, partial sequence

CAAAACTCCAAACCCATGTGAACATACCCTATGTTGCCTCGGCGGCGAGCGAAGTGCTCGGGAGGTGCCTACCCGGTACCTACCCTAGAGCTACCCTGTAGCTACCTACCCGGTAGTTGCGAGGTACCTACCCTGTAGCTACCCGGTAGCTACCCTGTAGGACTGGCGCATCCGCCGAAGGACCACTTAAACTCTTGTTTCTATGTGGCACGTCTGAGTAATTATATTTAATAAGTCAAAACTTTCAACAACGGATCTCTTGGTTCTGGCATCGATGAAGAACGCAGCGAAATGCGATAAGTAATGTGAATTGCAGAATTCAGTGAATCATCGAATCTTTGAACGCACATTGCGCCCATTAGTATTCTAGTGGGCATGCCTGTTCGAGCGTCATTTAAGTCCATTAAGCCTAGTTGCTTAGCGTTGGGAGCATTACCGTACGGTAACTCCTCAAAGTCAGTGGCAGGGTTGCAGGTGCACTCTTAGCGTAGTAGATTTATTACCGCTTACGATTGTCCCGTAGCCGTCGCCATAAGCCTATATTTTTTATTGGTTGACCTCGGATCAGGTAGGAATACCCGCTGAACTTAA

**SUB6288079 Seq14        MN450580**

**>Seq14 [organism=Cenococcum geophilum] [Ceno1] 18S ribosomal RNA gene, partial sequence; internal transcribed spacer 1, 5.8S ribosomal RNA gene, and internal transcribed spacer 2, complete sequence; and 28S ribosomal RNA gene, partial sequence**

ATATGCTTAAGTTCAGCGGGTATCCCTGCCTGATCCGAGGTCAACCTTGCGAGTAGGTCTTCCGGCAGACGGGGCCCCGACTTTTGAAGCGAAAGATTCTGCTACGCTTAAAGCCGGTGCCACGCCGCCGAGCGTTTCGAGGCGCGTCCCCAAAGGGGACGTCGCCCAACACCAAGCCAGGCTTGAGTGGTGAAATGACGCTCGAACAGGCATGCCCCTCGGGATACCAAGGGGCGCAATGTGCGTTCAAAGATTCGATGATTCACTGAATTCTGCAATTCACACTACTTATCGCATTTCGCTGCGTTCTTCATCGATGCCAGAACCAAGAGATCCGTTGTTGAAAGTTTTGATTCGTTGCTTGTAGCTCAGACAACACCGTTAAAACAGGAGTTTTGACGTATCCTCTGGCGGGAGAACCCGCCGAGGCAACATGAGTCAATCGTCAAAGGTTAGAAGTTCGCGGTTTGATCCGCGTTTACTTTCTGTAATGATCCTTCC

SUB6288079 Seq15        MN450581

>Seq15 [organism=Ilyonectria sp.] [Ily1] 18S ribosomal RNA gene, partial sequence; internal transcribed spacer 1, 5.8S ribosomal RNA gene, and internal transcribed spacer 2, complete sequence; and 28S ribosomal RNA gene, partial sequence

AAAGTCGTAACAAGGTCTCCGTTGGTGAACCAGCGGAGGGATCATTACCGAGTTTACAACTCCCAAACCCCTGTGAACATACCATATTGTTGCCTCGGCGGTGCCTGTTTCGGCAGCCCGCCAGAGGACCCAAACCCTAGATTACATTAAAGCATTTTCTGAGTCAATGATTAAATCAATCAAAACTTTCAACAACGGATCTCTTGGTTCTGGCATCGATGAAGAACGCAGCGAAATGCGATAAGTAATGTGAATTGCAGAATTCAGTGAATCATCGAATCTTTGAACGCACATTGCGCCCGCCAGTATTCTGGCGGGCATGCCTGTCCGAGCGTCATTTCAACCCTCAAGCCCCCGGGCTTGGTGTTGGAGATCGGCGAGCCCTCCGGGGCGCGCCGTCTCCCAAATATAGTGGCGGTCCCGCTGTAGCTTCCTCTGCGTAGTAGCACACCTCGCACTGGGAAACAGCGTGGCCACGCCGTAAAACCCCCCACTTCTGAAAGGTTGACCTCGGATCAGGTAGGAATACCCGCTGAACTTAA

SUB6288079 Seq16        MN450582

>Seq16 [organism=Ilyonectria sp.] [Ily2] 18S ribosomal RNA gene, partial sequence; internal transcribed spacer 1, 5.8S ribosomal RNA gene, and internal transcribed spacer 2, complete sequence; and 28S ribosomal RNA gene, partial sequence

ATATGCTTAAGTTCAGCGGGTATTCCTACCTGATCCGAGGTCAACCTTTCAGAAGTGGGGGGTTTCACGGCGTGGCCACGCTGTTTCCCAGTGCGAGGTGTGCTACTACGCAGAGGAAGCTACAGCGGGACCGCCACTATATTTGGGAGACGGCGCGCCCCGGAGGGCTCGCCGATCTCCAACACCAAGCCCGGGGGCTTGAGGGTTGAAATGACGCTCGGACAGGCATGCCCGCCAGAATACTGGCGGGCGCAATGTGCGTTCAAAGATTCGATGATTCACTGAATTCTGCAATTCACATTACTTATCGCATTTCGCTGCGTTCTTCATCGATGCCAGAACCAAGAGATCCGTTGTTGAAAGTTTTGATTGATTTAATCATTGACTCAGAAGATACTTTAATGTAATCTAGGGTTTGGGTCCTCTGGCGGGCTGCCGAAACAGGCACCGCCGAGGCAACAATATGGTATGTTCACAGGGGTTTGGGAGTTGTAAACTCGGTAATGATCCCTCCGCTGGTTCACCAACGGAGACCTTGTTACGACTTTTACTTCCTCTAAATGACCA

SUB6288079 Seq17        MN450583

>Seq17 [organism=Ilyonectria robusta] [Ily3] 18S ribosomal RNA gene, partial sequence; internal transcribed spacer 1, 5.8S ribosomal RNA gene, and internal transcribed spacer 2, complete sequence; and 28S ribosomal RNA gene, partial sequence

AAGGATCATTACTAGAGCAAAGGATAGGCAGCGCCCCACAGAAGCTTGCTTCGTGGCGGGCTACCCTTCTTAGGTTGAACTGGGGGATCATCTGAGTTAACAACTCCAACCCTCTGTGAACATACCATATTGTTGCCTCGGCGGTGCCTGTTTCGGCAGCCCGCCAGAGGACCCAAACCCTAGATTACATTAAAGCATTTTCTGAGTCAATGATTAAATCAATCAAAACTTTCAACAACGGATCTCTTGGTTCTGGCATCGATGAAGAACGCAGCGAAATGCGATAAGTAATGTGAATTGCAGAATTCAGTGAATCATCGAATCTTTGAACGCACATTGCGCCCGCCAGTATTCTGGCGGGCATGCCTGTCCGAGCGTCATTTCAACCCTCAAGCCCCCGGGCTTGGTGTTGGAGATCGGCGAGCCCTCCGGGGCGCGCCGTCTCCCAAATATAGTGGCGGTCCCGCTGTAGCTTCCTCTGCGTAGTAGCACACCTCGCACTGGGAAACAGCGTGGCCACGCCGTAAAACCCCCCACTTCTGAAAGGTTGACCTCGGATCAGTAGGAATACCCGCTGAACTTAA

SUB6288079 Seq18        MN450584

>Seq18 [organism=Ilyonectria sp.] [Ily4] 18S ribosomal RNA gene, partial sequence; internal transcribed spacer 1, 5.8S ribosomal RNA gene, and internal transcribed spacer 2, complete sequence; and 28S ribosomal RNA gene, partial sequence

AAGTCGTAACAAGGTCTCCGTTGGTGAACCAGCGGAGGGATCATTACCGAGTTTACAACTCCCAAACCCCTGTGAACATACCATTTGTTGCCTCGGCGGTGCCTGCTTCGGCAGCCCGCCAGAGGACCCAAACCCTTGATTTTATACAGTATCTTCTGAGTAAATGATTAAATAAATCAAAACTTTCAACAACGGATCTCTTGGTTCTGGCATCGATGAAGAACGCAGCGAAATGCGATAAGTAATGTGAATTGCAGAATTCAGTGAATCATCGAATCTTTGAACGCACATTGCGCCCGCCAGTATTCTGGCGGGCATGCCTGTTCGAGCGTCATTTCAACCCTCAAGCCCCCGGGCTTGGTGTTGGAGATCGGCGTGCCCCCCGGGGCGCGCCGGCTCCCAAATATAGTGGCGGTCTCGCTGTAGCTTCCTCTGCGTAGTAGCACACCTCGCACTGGAAAACAGCGTGGCCACGCCGTTAAACCCCCCACTTCTGAAAGGTTGACCTCGGATCAGGTAGGAATACCCGCTGAACTTAA

SUB6288079 Seq19        MN450585

>Seq19 [organism=Ilyonectria sp.] [Ily5] 18S ribosomal RNA gene, partial sequence; internal transcribed spacer 1, 5.8S ribosomal RNA gene, and internal transcribed spacer 2, complete sequence; and 28S ribosomal RNA gene, partial sequence

AAGTAAAAGTCGTAACAAGCTCCGTTGGTGAACCAGCGGAGGGATCATTACCGAGTTTACAACTCCCAAACCCCTTGAACATACGTATTTGTTGCCTCGGCGGTGCCTGTTCCGACAGCCCGCCAGAGGACCCCAAACCTGGATTACATTTAAGAAGTCTTTGAGTAAACCGATTAAATAAATCAAAACTTTCAACAACGGATCTCTTGGTTCTGGCATCGATGAAGAACGCAGCGAAATGCGATAAGTAATGTGAATTGCAGAATTCAGTGAATCATCGAATCTTTGAACGCACATTGCGCCCGCTAGTATTCTGGCGGGCATGCCTGTCCGAGCGTCATTTCAACCCTCAAGCCCCCGGGCTTGGTGTTGGGGATCGGCGAGCCTCCGCGCCCGCCGATCCCCTAAATCTAGTGGCGGTCTCGCTGTAGCTTCCTCTGCGTATTAGCACACCTCGCACTGGGAAACATCGCGGCCACGCCGTAACCCCCAACTTC

SUB6288079 Seq20        MN450586

>Seq20 [organism=Ilyonectria sp.] [Ily6] 18S ribosomal RNA gene, partial sequence; internal transcribed spacer 1, 5.8S ribosomal RNA gene, and internal transcribed spacer 2, complete sequence; and 28S ribosomal RNA gene, partial sequence

CGGAGGATATTACCGAGTTACAACTCCCAAACCCCCTGTGAACATACCTATTTGTTGCCTCGGGCGGTGCCTGTTCCGACAGCCCGCCAGAGGACCCCAAACCCTGATTACATTTAAGAAGTCTTCTGAGTAAACCGATTAAATAAATCAAAACTTTCAACAACGGATCTCTTGGTTCTGGCATCGATGAAGAACGCAGCGAAATGCGATAAGTAATGTGAATTGCAGAATTCAGTGAATCATCGAATCTTTGAACGCACATTGCGCCCGCTAGTATTCTGGCGGGCATGCCTGTCCGAGCGTCATTTCAACCCTCAAGCCCCCGGGCTTGGTGTTGGGGATCGGCGAGCCTCCGCGCCCGCCGTCCCCTAAATCTAGTGGCGGTCTCGCTGTAGCTTCCTCTGCGTAGTAGCACACCTCGCACTGGGAAACAGCGCGGCCACGCCGTTAAACCCCCAACTTCTGAACGTTTGACCTCGGATCAGGTAGGAATACCCGCTGAACTTAAGCATATCAATAGCGGAGGAA

SUB6288079 Seq21        MN450587

>Seq21 [organism=Penicillium murcianum] [Pen1] 18S ribosomal RNA gene, partial sequence; internal transcribed spacer 1, 5.8S ribosomal RNA gene, and internal transcribed spacer 2, complete sequence; and 28S ribosomal RNA gene, partial sequence

AAAAGTTTTGGTTGATCGGCAAGCGCCGGCCGGGCCTACAGAGCGGGTGACAAAGCCCCATACGCTCGAGGACCGGACGCGGTGCCGCCGCTGCCTTTCGGGCCCGTCCCCCCGGGAAGGGGGACGAGACCCAACACACAAGCCGGGCTTGAGGGCAGCAATGACGCTCGGACAGGCATGCCCCCCGGAATACCAGGGGGCGCAATGTGCGTTCAAAGACTCGATGATTCACTGAATTCTGCAATTCACATTACGTATCGCATTTCGCTGCGTTCTTCATCGATGCCGGAACCAAGAGATCCGTTGTTGAAAGTTTTAAATAATTTATATTTAGACTCAGACTGCAATTTTCATACAGAGTTCAAGGTGTCTTCGGCGGGCGCGGGCCCGGGGGCAGATGCCCCCCGGCGGCCGTGAGGCGGGCCCGCCGAAGCAACAAGGTACAATAAACACGGGTGGAGGTTGAATTCAGAGAATTCTCGCTCGGTAATGATCCTTCC

SUB6288079 Seq22        MN450588

>Seq22 [organism=Penicillium cinereoatrum] [Pen2] 18S ribosomal RNA gene, partial sequence; internal transcribed spacer 1, 5.8S ribosomal RNA gene, and internal transcribed spacer 2, complete sequence; and 28S ribosomal RNA gene, partial sequence

TGCGGAGGATATTACCGAGTGAGGGCCCTCTGGGTCACCTCCCACCCGTTTTTATCGTACCTTGTTGCTTCGGCGGGCCCGCCTCACGGCCGCCGGGGGGCTTCTGCCCCCTGGCCCGCGCCCGCCGAAGACACCATTGAACGCTGTCTGAAGATTGCAGTCTGAGCAATTAGCTAAATAAGTTAAAACTTTCAACAACGGATCTCTTGGTTCCGGCATCGATGAAGAACGCAGCGAAATGCGATACGTAATGTGAATTGCAGAATTCAGTGAATCATCGAGTCTTTGAACGCACATTGCGCCCCCTGGTATTCCGGGGGGCATGCCTGTCCGAGCGTCATTGCCGCCCTCAAGCACGGCTTGTGTGTTGGGCCTCCGTCCTCCTCCCGGGGGACGGGCCCGGGCAGCGGCGGCACCGCGTCCGGTCCTCGAGCGTATGGGGCTTCGTCACCCGCTCTTGTAGGCCCGGCCGGCGCTGCCGACAACATCAATCTTTT

SUB6288079 Seq23        MN450589

>Seq23 [organism=Penicillium janthinellum] [Pen3] 18S ribosomal RNA gene, partial sequence; internal transcribed spacer 1, 5.8S ribosomal RNA gene, and internal transcribed spacer 2, complete sequence; and 28S ribosomal RNA gene, partial sequence

AAGGATCATTACCGAGTGAGGGCCCTCTGGGTCCAACCTCCCACCCGTGTTTATCGTACCTTGTTGCTTCGGTGGGCCCGCCTCACGGCCGCCGGGGGGCATCTGCCCCCGGGCCCGCGCCCGCCGAAGACACCATTGAACTCTGTCTGAAGATTGCAGTCTGAGCGATTAACTAAATCAGTTAAAACTTTCAACAACGGATCTCTTGGTTCCGGCATCGATGAAGAACGCAGCGAAATGCGATAAGTAATGTGAATTGCAGAATTCAGTGAATCATCGAGTCTTTGAACGCACATTGCGCCCCCTGGTATTCCGGGGGGCATGCCTGTCCGAGCGTCATTGCTGCCCTCAAGCACGGCTTGTGTGTTGGGCTCCGCCCCCCTCCCGGGGGGCGGGCCCGAAAGGCAGCGGCGGCACCGCGTCCGGTCCTCGAGCGTATGGGGCTTTGTCACCCGCTCTGTAGGCCCGGCCGGCGCCCGCCGGCGACCCCAATCAATCTTTCCAGGTTGACCTCGGATCAGGTAGGGATACCCGCTGAACTTAA

SUB6288079 Seq24        MN450590

>Seq24 [organism=Penicillium pancosmium] [Pen4] 18S ribosomal RNA gene, partial sequence; internal transcribed spacer 1, 5.8S ribosomal RNA gene, and internal transcribed spacer 2, complete sequence; and 28S ribosomal RNA gene, partial sequence

GGAAGGATCATTACTGAGTGAGGGCCCCTCGGGGTCCAACCTCCCACCCGTGTTTAACGTACCTTGTTGCTTCGGCGGGCCCGCCTCACGGCCGCCGGGGGGCATCTGCCCCCGGGCCCGCGCCCGCCGAAGCCACCTGTGAACGCTGTCTGAAGTATGCAGTCTGAGACAATTATTAAATTAATTAAAACTTTCAACAACGGATCTCTTGGTTCCGGCATCGATGAAGAACGCAGCGAAATGCGATAACTAATGTGAATTGCAGAATTCAGTGAATCATCGAGTCTTTGAACGCACATTGCGCCCTCTGGTATTCCGGAGGGCATGCCTGTCCGAGCGTCATTGCTGCCCTCCAGCCCGGCTGGTGTGTTGGGCCCCGCCCCCCTTCCCGGGGGGGCGGGCCCGAAAGGCAGCGGCGGCACCGCGTCCGGTCCTCGAGCGTATGGGGCTTTGTCACCCGCTCTTGCAGGCCCGGCCGGCGCCAGCCGACCCCCTCAATCTATTTTTTCAGGTTGACCTCGGATCAGGTAGGGATACCCGCTGAACTTAA

SUB6288079 Seq25        MN450591
>Seq25 [organism=Penicillium sp.] [Pen5] 18S ribosomal RNA gene, partial sequence; internal transcribed spacer 1, 5.8S ribosomal RNA gene, and internal transcribed spacer 2, complete sequence; and 28S ribosomal RNA gene, partial sequence

CGGGTGACAAAGCCCCATACGCTCGAGGACCGGACGCGGTGCCGCCGCTGCCTTTCGGGCCCGCCCCCCGGGAGGGGGGCGGAGCCCAACACACAAGCCGTGCTTGAGGGCAGCAATGACGCTCGGACAGGCATGCCCCCCGGAATACCAGGGGGCGCAATGTGCGTTCAAAGACTCGATGATTCACTGAATTCTGCAATTCACATTACTTATCGCATTTCGCTGCGTTCTTCATCGATGCCGGAACCAAGAGATCCGTTGTTGAAAGTTTTAACTGATTTAGTTAATCGCTCANACTGCAATCTTCAGACAGAGTTCAATGGTGTCTTCGGCGGGCGCGGGCCCGGGGGCAGATGCCCCCCGGCGGCCGTGAGGCGGGCCCACCGAAGCAACAAGGTACGATAAACACGGGTGGGAGGTTGGACCCAGAGGGCCCTCACTCGGTAATGATCCTTCCGCAGGTTCACCTACGG

SUB6288079 Seq26        MN450592

>Seq26 [organism=Penicillium swiecickii] [Pen6] 18S ribosomal RNA gene, partial sequence; internal transcribed spacer 1, 5.8S ribosomal RNA gene, and internal transcribed spacer 2, complete sequence; and 28S ribosomal RNA gene, partial sequence

AAGGATCATTACCGAGTGAGGGCCCTCTGGGTCCAACCTCCCACCCGTGTTTATTATACCTTGTTGCTTCGGCGGGCCCGCCGTATGGCCGCCGGGGGGCTTCTGCCCCCGGGCCCGCGCCCGCCGAAGACATCTCGAACTCTGTCTGAAGATTGTAGTCTGAGTAAAAATATAAATTATTTAAAACTTTCAACAACGGATCTCTTGGTTCCGGCATCGATGAAGAACGCAGCGAAATGCGATACGTAATGTGAATTGCAGAATTCAGTGAATCATCGAGTCTTTGAACGCACATTGCGCCCTCTGGTATTCCGGAGGGCATGCCTGTCCGAGCGTCATTGCTGCCCTCAAGCACGGCTTGTGTGTTGGGCTCCGTCCCCCGATCCCGGGGGACGGGCCCGAAAGGCAGCGGCGGCACCGCGTCCGGTCCTCGAGCGTATGGGGCTTTGTCACCCGCTCTGTAGGCCCGGCCGGCGCTTGCCGATCAACCAAATTTTATCCAGGTAC

SUB6288079 Seq27        MN450593

>Seq27 [organism=Penicillium bilaiae] [Pen7] 18S ribosomal RNA gene, partial sequence; internal transcribed spacer 1, 5.8S ribosomal RNA gene, and internal transcribed spacer 2, complete sequence; and 28S ribosomal RNA gene, partial sequence

AAGGATCATTACTGAGTGAGGGCCCTCTGGGTCCAACCTCCCACCCGTGTCTCTTGTACCATGTTGCTTCGGCGAGCCCGCCTCACGGCCGCCGGGGGGCATCTGCCCCCGGGCCCGCGCCCGCCGAAGCCCCCTCTGAACGCTGTCTGAAGATTGCAGTCTGAGCGATAAGCAAAAATTATTTAAAACTTTCAACAACGGATCTCTTGGTTCCGGCATCGATGAAGAACGCAGCGAAATGCGATAACTAATGTGAATTGCAGAATTCAGTGAATCATCGAGTCTTTGAACGCACATTGCGCCCCCTGGTATTCCGGGGGGCATGCCTGTCCGAGCGTCATTGCTGCCCTCAAGCACGGCTTGTGTGTTGGGCCTCCGTCCTCCCCCCGGGGGGACGGGCCCGAAAGGCAGCGGCGGCACCGTGTCCGGTCCTCGAGCGTATGGGGCTTTGTCACCCGCTCTGTAGGCCCGGCCGGCGCTGGCCGACCCTCCAACCCCATTTTCAGGTACTCGA

SUB6288079 Seq28        MN450594

>Seq28 [organism=Penicillium Carminoviolaceum] [Pen8] 18S ribosomal RNA gene, partial sequence; internal transcribed spacer 1, 5.8S ribosomal RNA gene, and internal transcribed spacer 2, complete sequence; and 28S ribosomal RNA gene, partial sequence

AAGTCGTAACAAGGTTTCCGTAGGTGAACCTGCGGAAGGATCATTACCGAGTGAGGGCCCTCTGGGTCCAACCTCCCACCCGTGTTTAACGAACCTTGTTGCTTCGGCGGGCCCGCCTCACGGCCGCCGGGGGGCATCCGCCCCCGGGCCCGCGCCCGCCGAAGACACCTGTGAACACTGTCTGAAGTTGCAGTCTGAGAAACTAGCTAAATTAGTTAAAACTTTCAACAACGGATCTCTTGGTTCCGGCATCGATGAAGAACGCAGCGAAATGCGATAAATAATGTGAATTGCAGAATTCAGTGAATCATCGAGTCTTTGAACGCACATTGCGCCCTCTGGTATTCCGGAGGGCATGCCTGTCCGAGCGTCATTGCTGCCCTCAAGCACGGCTTGTGTGTTGGGCCCCCGTCCCCCCCACCGGGGGGACGGGCCCGAAAGGCAGCGGCGGCACCGCGTCCGGTCCTCGAGCGTATGGGGCTCTGTCACCCGCTCTTGTAGGCCCGGCCGGCGCCAGCCGACCCCCCTCAATCTATTTTTCAGGTGACCTCGATCAGTAGGGATACCCGCTGAAACTTAA

SUB6288079 Seq29        MN450595

>Seq29 [organism=Penicillium sp.] [Pen9] 18S ribosomal RNA gene, partial sequence; internal transcribed spacer 1, 5.8S ribosomal RNA gene, and internal transcribed spacer 2, complete sequence; and 28S ribosomal RNA gene, partial sequence

AAGTCGTAACAAGGTTTCCGTAGGTGAACCTGCGGAAGGATCATTACTGAGTGAGGGCCCTCTGGGTCCAACCTCCCACCCGTGTTTATTGTACCTTGTTGCTTCGGCGGGCCCGCCTTTATGGCCGCCGGGGGGCTTACGCTCCCGGGCCCGCGCCCGCCGAAGACACCCTCGAACTCTGTCTGAAGATTGTAGTCTGAGTGAAAATATAAATTATTTAAAACTTTCAACAACGGATCTCTTGGTTCCGGCATCGATGAAGAACGCAGCGAAATGCGATACGTAATGTGAATTGCAGAATTCAGTGAATCATCGAGTCTTTGAACGCACATTGCGCCCCCTGGTATTCCGGGGGGCATGCCTGTCCGAGCGTCATTGCTGCCCTCAAGCACGGCTTGTGTGTTGGGCCCCGTCCTCCGATCCCGGGGGACGGGCCCGAAAGGCAGCGGCGGCACCGCGTCCGGTCCTCGAGCGTATGGGGCTTTGTCACCCGCTCTGTAGGCCCGGCCGGCGCTTGCCGATCAACCAAAATTTTTTCCAGGTTGACCTCGGATCAGTCTAGGGATACCCGCTGAACTTAA

SUB6288079 Seq30        MN450596

>Seq30 [organism=Penicillium sp.] [Pen10] 18S ribosomal RNA gene, partial sequence; internal transcribed spacer 1, 5.8S ribosomal RNA gene, and internal transcribed spacer 2, complete sequence; and 28S ribosomal RNA gene, partial sequence

AAGTCGTAACAAGGTTTCCGTAGGTGAACCTGCGGAAGGATCATTACCGAGCGAGAATTCTCTGAATTCAACCTCCCACCCGTGTTTATTGTACCTTGTTGCTTCGGCGGGCCCGCCTCACGGCCGCCGGGGGGCATCTGCCCCCGGGCCCGCGCCCGCCGAAGACACCTTGAACTCTGTATGAAAATTGCAGTCTGAGTCTAAATATAAATTATTTAAAACTTTCAACAACGGATCTCTTGGTTCCGGCATCGATGAAGAACGCAGCGAAATGCGATACGTAATGTGAATTGCAGAATTCAGTGAATCATCGAGTCTTTGAACGCACATTGCGCCCCCTGGTATTCCGGGGGGCATGCCTGTCCGAGCGTCATTGCTGCCCTCAAGCCCGGCTTGTGTGTTGGGTCTCGTCCCCCTTCCCGGGGGGACGGGCCCGAAAGGCAGCGGCGGCACCGCGTCCGGTCCTCGAGCGTATGGGGCTTTGTCACCCGCTCTGTAGGCCCGGCCGGCGCTTGCCGATCAACCAAAACTTTTTCCAGGTTGACCTCGATCAGGTAGGGATACCCGCTGAACTTAA

SUB6288079 Seq31        MN450597

>Seq31 [organism=Penicillium fellutanum] [Pen11] 18S ribosomal RNA gene, partial sequence; internal transcribed spacer 1, 5.8S ribosomal RNA gene, and internal transcribed spacer 2, complete sequence; and 28S ribosomal RNA gene, partial sequence

AAGGATCATTATGAGTGGGGCCCTTTGGGTCCAACCTCCCACCCGTGTATACTTACCGTGTTGCTTCGGCGGGCCCGCCTGTCAGGCCGCCGGGGGGCAACCGCCCCCGGGCCCGCGCCCGCCGAAGACCCCAACGAATCTTGTACCTTGCAGTGTGAGCGATAAGCATAAATTATTAAAACTTTCAACAACGGATTCTTGGTTCCGGCATCGATGAAGAACGCAGCGAAATGCGATAAGTAATGTGAATTGCAGAATTCAGTGAATCATCGAGTCTTTGAACGCACATTGCGCCCCCTGGTATTCCGGGGGGCATGCCTGTCCGAGCGTCATTACTGCCCTCAAGCCCGGCTTGTGTGTTGGGCGCCGCCCCCCCGGGGGCGGGCCCGAAAGGCAGCGGCGGCACCGCGTCCGGTCCTCG

SUB6288079 Seq32        MN450598

>Seq32 [organism=Penicillium sp.] [Pen12] 18S ribosomal RNA gene, partial sequence; internal transcribed spacer 1, 5.8S ribosomal RNA gene, and internal transcribed spacer 2, complete sequence; and 28S ribosomal RNA gene, partial sequence

GGCCCTCTGGGTCCAACCTCCCACCCGTGTTTATCGTACCTTGATTGCTTCGGCGGGCCCGCCTAGGCCGCCGGGGGGCTCTGCCCTCGGGCCCGCGCCCGCCGAAGACACCATTGAACGCTGTCTGAAGATTGCAGTCTGAGCAATTAGCTAAATAAGTTAAAACTTTCAACAACGGATCTCTTGGTTCCGGCATCGATGAAGAACGCAGCGAAATGCGATACGTAATGTGAATTGCAGAATTCAGTGAATCATCGAGTCTTTGAACGCACATTGCGCCCCCTGGTATTCCGGGGGGCATGCCTGTCCGAGCGTCATTGCTGCCCTCAAGCACGGCTTGTGTGTTGGGCCTCCGTCCTCTCCCGGGGGACGGGCCCGAAAGGCACGGCGGCACCGCGTCCGGTCCTCACGTATGGGGCTTCGTCACCCGCTCTTGTAGGCCCGGCCGGCGCTTGCCGGACACATCAATCTTTTTTCCAGGTTGACCTCGGATCAGTAGGGATA

SUB6288079 Seq33        MN450599

>Seq33 [organism=Penicillium restrictum] [Pen13] 18S ribosomal RNA gene, partial sequence; internal transcribed spacer 1, 5.8S ribosomal RNA gene, and internal transcribed spacer 2, complete sequence; and 28S ribosomal RNA gene, partial sequence

TCCGTAAGTGAACCTGCGGAAGGATCATTACCGAGTGAGGGCCCTATGGGTCCAACCTCCCACCCGTGTTTATCGTACCTTGTTGCTTCGGCGGGCCCCCTCACGGCCGCCGGGGGGCTTCTGCCCTCGGGCCCGCGCCCGCCGAAGACACCATTGAACGCTGTCTGAAGATTGCAGTCTGAGCAATTAGCTAAATAAGTTAAAACTTTCAACAACGGATCTCTTGGTTCCGGCATCGATGAAGAACGCAGCGAAATGCGATACGTAATGTGAATTGCAGAATTCAGTGAATCATCGAGTCTTTGAACGCACATTGCGCCCCCTGGTATTCCGGGGGGCATGCCTGTCCGAGCGTCATTGCTGCCCTCAAGCACGGCTTGTGTGTTGGGCCTCCGTCCTCCTCCCGGGGGACGGGCCCGAAAGGCAGCGGCGGCACCGCGTCCGGTCCTCGAGCGTATGGGGCTTCGTCACCCCTCTTGTAGGCCCGGCCGGCGCTTGCCCGACACATCAATCTTTTTTCCAGTTGCTCGGATCAGGT

SUB6288079 Seq34        MN450600

>Seq34 [organism=Penicillium sp.] [Pen14] 18S ribosomal RNA gene, partial sequence; internal transcribed spacer 1, 5.8S ribosomal RNA gene, and internal transcribed spacer 2, complete sequence; and 28S ribosomal RNA gene, partial sequence

CCCTCTGGGTCCAACCTCCCACCCCMAMTATATCGTACCTTGTTGCTTCGGCGGGCCCGCCATCACGGCCGCCGGGGGGCATCCGCCCCCGGGCCCGCGCCCGCCGAAGACACCATTGAACGCTGTCTGAAGATTGCAGTCTGAGCGAAAAGCAAAATTAGTTAAAACTTTCAACAACGGATCTCTTGGTTCCGGCATCGATGAAGAACGCAGCGAAATGCGATAAGTAATGTGAATTGCAGAATTCAGTGAATCATCGAGTCTTTGAACGCACATTGCGCCCCCTGGTATTCCGGGGGGCATGCCTGTCCGAGCGTCATTGCTGCCCTCAAGCACGGCTTGTGTGTTGGGCCCCCGTCCCCCGACTCCCGGGGGACGGGCCCGAAAGGCAGCGGCGGCACCGCGTCCGGTCCTCGAGCGTATGGGGCTTCGTCACCCGCTCTGTAGGCCCGGCCGGCGCCTGCCGACCATCAACCCTTTTTTTTTCAGGTTGACCTCGGATCAGGTAGGGATACCCGCTGAACTTAA

SUB6288079 Seq35        MN450601

>Seq35 [organism=Ascotricha erinacea] [Asc1] 18S ribosomal RNA gene, partial sequence; internal transcribed spacer 1, 5.8S ribosomal RNA gene, and internal transcribed spacer 2, complete sequence; and 28S ribosomal RNA gene, partial sequence

TTGGGGCTTTTATGGCGAGAAGGCCAGGACAGCTTCAGAAGCGAGACTTGTAAAGTTTACTACGCTTAGAGCATATCCTAACTCTGCCAGCTAGCTTTGGGGAGTTGCGTTTCACGGCAAGCTCCCAACACCAAGCAACTAGGGCTTGAGGGCTGAAATGACGCTCGAACAGGCATGCCTACTAGAATACTAATAGGCGCAATGTGCGTTCAAAGATTCGATGATTCACTGAATTCTGCAATTCACATTACTTATCGCATTTCGCTGCGTTCTTCATCGATGCCAGAACCAAGAGATCCGTTGTTGAAAGTTTTAACTTATTAAGTTTATGCAACTCAGAGTTCCACAATAATAACAAGAGTTTGGTAGTCCACCGGCAGGCCACCACCGAGGTCGCGATGACCTCAACCAGGCCCTGCCGAGGCAACAAAAGGTAAGTTCACATGGGTTTGGGAGTTTT

SUB6288079 Seq36        MN450602

>Seq36 [organism=Cladophialophora sp.] [Cladphor1] 18S ribosomal RNA gene, partial sequence; internal transcribed spacer 1, 5.8S ribosomal RNA gene, and internal transcribed spacer 2, complete sequence; and 28S ribosomal RNA gene, partial sequence

AGTTAGGGTCTTCCAGGCCCGACCTCCAACCCTTTGCCTACCTTACCTCTTGTTGCTTCGGCCGGCCCGTCCCCCCTAGAAATAGGGGTCGACCGCCGGAGGGCTCACACCCTCTGGCCCGCGCCCGCCGATGGCCCTCAACCAAAACTCTTGTTCAATCGTGAATTGTCTGAGTATACAAAACAAAATAAACCAAAAACTTTCAACAACGGATCTCTTGGTTCTGGCATCGATGAAGAACGCAGCGAAATGCGATAAGTAATGCGAATTGCAGAATCCGTGAGTCATCGAATCTTTGAACGCACATTGCGCCCCTTGGTTATTCCGAAGGGCATGCCTGTTCGAGCGTCATTATCACCCCTCAAGCCCTGGCTTGGTGTTGGACTTTAGTTATAGCTTTGCCTATCAACTGGTCTCAAAGATAGTGACGGCGCCCCAGAGGGAACCGGGTACGAGGAGCTATTTACTAAGCATGTATCTGGGACGACTCTGATGGGCGACGGTCTTGTTTACTTTTACCTAAGTGT

SUB6288079 Seq37        MN450603

>Seq37 [organism=Helotiales sp.] [Helo1] 18S ribosomal RNA gene, partial sequence; internal transcribed spacer 1, 5.8S ribosomal RNA gene, and internal transcribed spacer 2, complete sequence; and 28S ribosomal RNA gene, partial sequence

AAGGATCATTACAGAGTTCATGCCCTCACGGGTAGATCTCCCACCCTTGAATATTTTATACCTTTGTTGCTTTGGCGGGCCGCTTCGGCTACCGGCTTTGGCTGGTGAGTGCCCGCCAGAGGACCCCAAATTCTGAATTATAGTGTCGTCTGAGTACTATAAAATAGTTAAAACTTTCAACAACGGATCTCTTGGTTCTGGCATCGATGAAGAACGCAGCGAAATGCGATAAGTAATGTGAATTGCAGAATTCAGTGAATCATCGAATCTTTGAACGCACATTGCGCCCCTTGGTATTCCGAGGGGCATGCCTGTTCGAGCGTCATTATAACCCCTCAAGCTCAGCTTGGTGTTGGGGCCTGCCGCACGGGCAGCCCTTAAAATCAGTGGCGGTGCCATCTGGCTCTAAGCGTAGTAATTCTTCTCGCTATAGAGTCACGGTGGATGCTTGCCATTAACCCCCAATTTTCAATGGTTGACCTCGGATCAGGTAGGGATACCCGCTGAACTTAA

SUB6288079 Seq38        MN450604>Seq38 [organism=Ochronconis globalis] [Och1] 18S ribosomal RNA gene, partial sequence; internal transcribed spacer 1, 5.8S ribosomal RNA gene, and internal transcribed spacer 2, complete sequence; and 28S ribosomal RNA gene, partial sequence

TTTCTGTGGAGACGGTTACCCGACCTTTTGTGTGATTTGAGGGTCCGTTTCGGTGTTGAGCGCCGACGGGCGAAGGTATTTCGGCGGAAGCGGTAGGCGTTTTCGAATCTTTCCCCTCTACGTTCTTCTTCTCGAGAGAGTCAGAAGGGGAAGGGGAAGAGGGCAGGAGACGACCTGCCCGCCGCCGGCGGTATTGAACCCAACTCTATGCGAGAAAATGAAGTCTGAAGGCGCGGACCGTGGTGCGCGAGCGCGACGGGAAACGCGAGAGAAGCAAAAGGAAAAAGAAGCAAAACTTTCAACAACGGATCTCTTGGCTCTGGCAACGATGAAGAACGCAGCGAAACGCGAAAGGTAATGCGAATTGCAGAATCAGTGAGTCATCGAATTTTTGAACGCACATTGCGCCTCTCGGTATTCCGGGAGGCACGCCTGTTCGAGCGCCATTACACCCTCCAAGCTGCGGCTTGATGTTGGGCGAGGGTCTTCCCGTTCCCGTGGGGTGGGGAGGACGCGCCTGGAACGCGTGGGCAGCGGAAGCCAGACCGCGAGCGTAGCGAGACACGAAGAGTCAAGTCGCTCGGGACGGACCTGGTGGACGTGT

SUB6288079 Seq39        MN450605

>Seq39 [organism=Thielaviopsis basicola] [Thiel1] 18S ribosomal RNA gene, partial sequence; internal transcribed spacer 1, 5.8S ribosomal RNA gene, and internal transcribed spacer 2, complete sequence; and 28S ribosomal RNA gene, partial sequence

AGGGATCATTATCGAGTTTTTAACTCTTAAACCATATGTGAACATACCTTTTCTAGCTGCTTTGGCAGGTGCCTCTCGGGGCTTCTGCCGGTAGCATTTATAAACTCTTTATATTTCTATAGAATTATTCATTGCTGAGTGGCATTAACTAAATAAGTTAAAACTTTCAACAACGGATCTCTTGGCTCTAGCATCGATGAAGAACGCAGCGAAATGCGATAAGTAATGTGAATTGCAGAATTCAGTGAATCATCGAATCTTTGAACGCACATTGCGCCTGGCAGTATTCTGCCAGGCATGCCTGTCCGAGCGTCATTTCACCACTCAAGCTCTGCTTGGTGTTGGAGGACCCGCGTTTAGTCGCGGGCCGCCGAAATGCATCGGCTGTTGTATATACAGCTTCCCTGTGTAGTAAATGCTTAGCTTTACACTTTGAAACTTTTATATAACATGCCGGAAACCCTCAACTTTTGAAAGGTTGACCTCGATCAGGTAGGAATACCCGCTGAACTTAA

SUB6288079 Seq40        MN450606

>Seq40 [organism=Cordana pauciseptata] [Cord1] 18S ribosomal RNA gene, partial sequence; internal transcribed spacer 1, 5.8S ribosomal RNA gene, and internal transcribed spacer 2, complete sequence; and 28S ribosomal RNA gene, partial sequence

AAGGATCATTACTTCCGTAGGTGAACCTGCGGAGGGATCATTACAGAGTTGCAAAACTCCCAACCTTGTGAACATACCCGTTACAGTTGCTTCGGCGGGCAGCCCCAGGGCGGGGCTGCAGCCTCTTCTCACGAGGCGCCCGCCGGAGGTATATAACTCTTTGTATTTGTTGGTCTCTCTGAGTAAATTTATAATAAGTCAAAACTTTTAACAACGGATCTCTTGGTTCTGGCATCGATGAAGAACGCAGCGAAATGCGATAAGTAATGTGAATTGCAGAATTCAGTGAATCATCGAATCTTTGAACGCACATTGCGCCCGCTAGTATTCTGGCGGGCATGCCTGTTCAGCGTCATTTCAACCCTCAAGCCCTGCTTGGTGTTGGGGTCCTACAGTAATGTAGGCCCTGAAAACTAGTGGCGGGCTCGCTATAACTCCGAGCGTAGTAGTAATATCTCGTTTAGGAAGTGTAGCGGGTTCTTGCCGTTAAACCCCCCATTTTATAAGGTTGACCTCGGATCAGGTAGGAATACCCGCTGAACTTAA

SUB6288079 Seq41        MN450607

>Seq41 [organism=Helotiaceae sp.] [Helot1] 18S ribosomal RNA gene, partial sequence; internal transcribed spacer 1, 5.8S ribosomal RNA gene, and internal transcribed spacer 2, complete sequence; and 28S ribosomal RNA gene, partial sequence

AAGGATCATTACAGAGTTCGTGCCCTTCGGGGTAGACCTCCCACCCTTTGTATACCTACCTTTGTTGCTTTGGCGGGCCGTCGCAAGACCGGCGGCTTCGGCTGTCGTGTGCCCGCCAGAGGACCCCAAACTCTGAATACAGTGTCGTCTGAGTACTATATAATAGTTAAAACTTTCAACAACGGATCTCTTGGTTCTGGCATCGATGAAGAACGCAGCGAAATGCGATAAGTAATGTGAATTGCAGAATTCAGTGAATCATCGAATCTTTGAACGCACATTGCGCCCCTTGGTATTCCGGGGGGCATGCCTCTTCCAGCGTCATTTCACCCCTCAAGCTCTGCTTGGTTTTGGGCCGCGCCGGCAACGGCGGGCCTCGAAAATAGTGGCGACGCCATCGTGCTCTCAGCGTAGTAATTCTTCTCGCTGTTGGGTCCCGGTGGTCGTCCGCCAGCAACCCCCAACTTTCTTAAGTTTGACCTGGGATGAGGTAGGGATACCCGCTGAACTTAA

SUB6288079 Seq42        MN450608

>Seq42 [organism=Cordyceps sp.] [Cordy1] 18S ribosomal RNA gene, partial sequence; internal transcribed spacer 1, 5.8S ribosomal RNA gene, and internal transcribed spacer 2, complete sequence; and 28S ribosomal RNA gene, partial sequence

TCCGTAGGTGAACCTGCGGAGGGATCATTATCGAGTGTAAAAACTCACCAAACCCTGTGAACATACCTTCTGTTGCTTCGGCGGGATCGCCCCGGGCGCCTTCGTGTGCCCCGGACTCAGGCGCCCGCCGGAGGACCTAAACTCTTGTTTTTATGAGATTTATCTGAGTGGCTTTTATAGCAAAAATGAATCAAAACTTTCAACAACGGATCTCTTGGTTCTGGCATCGATGAAGAACGCAGCGAAATGCGATAAGTAATGTGAATTGCAGAATCCAGTGAATCATCGAATCTTTGAACGCACATTGCGCCCGCCAGTATTCTGGCGGGCATGCCTGTCTGAGCGTCATTTCAACCCTCGCAACCAGCTTGCTGGATGCGGTGTTGGGGATCGGCCCCCCGTACTGGGAGGCCGTCCCTTAAATAGAGTGGCGGTTGCGCTGTGACCTCCTCTGCGTAGTAGCAATATCTCGCAGGCGGATACGGTGAACCACGCCGAAAACCCCCAACTCTTCTTT

SUB6288079 Seq43        MN450609

>Seq43 [organism=Mortierella alpina] [Mort1] 18S ribosomal RNA gene, partial sequence; internal transcribed spacer 1, 5.8S ribosomal RNA gene, and internal transcribed spacer 2, complete sequence; and 28S ribosomal RNA gene, partial sequence

ATCATTCATAATCAAGTGTTTTTATGGCACTTTCAAAAATCCATATCCACCTTGTGTGCAATGTCATCTCACTGGAGGTCGACGGCTGTAAAAATCCGTTTGGTCACCTTTGGGATTTATATCTACTCAGAACTTTAGTGATTTTGTCTGAAACATATTATGAATACTTAATTCAAAATACAACTTTCAACAACGGATCTCTTGGCTCTCGCATCGATGAAGAACGCAGCGAAATGCGATACGTAATGTGAATTGCAGAATTCAGTGAATCATCGAATCTTTGAACGCATATTGCGCTCTCTGGTATTCCGGAGAGCATGCTTGTTTGAGTATCAGTAAACACCTCAACTCCCCTTTCTTTTTTGAAATGGGAGCTGGACTTGAGTGATCCCAACGCTTTTCTCACCGAAAAGTGGCGGGTTACTTGAAATGCAGGTGCAGCTGGACTTTTCTCTGAGCTAAAAGCATATCTATTTAGTCTGCCTAAAAAACAGATTATTACCTTTGCTGCAGCTAACATAAAGGAGATTAGTTCTTGTGCTGACTGATGCAGGATTCACAGAGACAGCTTCGGCTGGCCTTTGTAAACTCGATCTCAATCAAAGTAAGACTACCCGCTGAACTTAA

SUB6288079 Seq44        MN450610

>Seq44 [organism=Mortierella sp.] [Mort2] 18S ribosomal RNA gene, partial sequence; internal transcribed spacer 1, 5.8S ribosomal RNA gene, and internal transcribed spacer 2, complete sequence; and 28S ribosomal RNA gene, partial sequence

GCCGACCCTTGTGAAATCCTGCATCCCTCAGCCAATACGGTCAAACTCCCTTTATGTTAGCTGCAGCAAAAGTAATAATCCGTTGACGGGACTAAATAAATATGCTTTTAGCTCAGGAGAATGTCCAGCTGCACCTGCATTTCAAGCAACCCTCCACCGATCGTAAAGACTGGTGTTGGGATTGCTCAAGTCCAAAGCCATTCAAAACAAATTGAAGTCTTTGAGGTGTTTACTGATACTCAAACAAGCATGCTCTTCGGAATACCAAAGAGCGCAATATGCGTTCAAAGATTCGATGATTCACTGAATTCTGCAATTCACATTACGTATCGCATTTCGCTGCGTTCTTCATCGATGCGAGAGCCAAGAGATCCGTTGTTGAAAGTTGTATTTTGAATTATTTTATTCATAATATTTTTCAGACAAAGAGTTAAAATTTAAGTTGATGTTTGGTCATTCTCAAAAAAGAGAACAACTGACATTGCACACAAGGTGGATATGGATTTAAAAAGTGCCATAAAACACTTATTATGAATGATCCTTCCGCAGGTTCACCTACGG

SUB6288079 Seq45        MN450611

>Seq45 [organism=Mortierella sp.] [Mort3] 18S ribosomal RNA gene, partial sequence; internal transcribed spacer 1, 5.8S ribosomal RNA gene, and internal transcribed spacer 2, complete sequence; and 28S ribosomal RNA gene, partial sequence

GGCACTTTTTAAATCCATATCCACCTTGTGTGCAATGTCAGTTGTTCTCTTTTTTGAGAATGACCAAACATCAACTTAAATTTTAACTCTTTGTCTGAAAAATATTATGAATAAAATAATTCAAAATACAACTTTCAACAACGGATCTCTTGGCTCTCGCATCGATGAAGAACGCAGCGAAATGCGATACGTAATGTGAATTGCAGAATTCAGTGAATCATCGAATCTTTGAACGCATATTGCGCTCTTTGGTATTCCGAAGAGCATGCTTGTTTGAGTATCAGTAAACACCTCAAAGACTTCAATTTGTTTTGAATGGCTTTGGACTTGAGCAATCCCAACACCAGTCTTTACGATCGGTGGAGGGTTGCTTGAAATGCAGGTGCAGCTGGACATTCTCCTGAGCTAAAAGCATATTTATTTAGTCCCGTCAAACGGATTATTACTTTTGCTGCAGCTAACATAAAGGGAGTTTGACCGTATTGGCTGACTGATGCAGGATTTCACAAGGGTCGGCAACGATTCTTGTAAAACTCGATCTCAAATCAAGTAAGACTACCCGCTGAAC

SUB6288079 Seq46        MN450612

>Seq46 [organism=Dactylonectria alcacerensis] [Dactyl1] 18S ribosomal RNA gene, partial sequence; internal transcribed spacer 1, 5.8S ribosomal RNA gene, and internal transcribed spacer 2, complete sequence; and 28S ribosomal RNA gene, partial sequence

AAAGTCGTAACAAGGTCTCCGTTGGTGAACCAGCGGAGGGATCATTACCGAGTTTACAACTCCCAAACCCCTGTGAACATACCTATTTGTTGCCTCGGCGGTGCCTGTTCCGACAGCCCGCCAGAGGACCCCAAACCCTGATTACATTTAAGAAGTCTTCTGAGTAAACCGATTAAATAAATCAAAACTTTCAACAACGGATCTCTTGGTTCTGGCATCGATGAAGAACGCAGCGAAATGCGATAAGTAATGTGAATTGCAGAATTCAGTGAATCATCGAATCTTTGAACGCACATTGCGCCCGCTAGTATTCTGGCGGGCATGCCTGTCCGAGCGTCATTTCAACCCTCAAGCCCCCGGGCTTGGTGTTGGGGATCGGCGAGCCTCCGCGCCCGCCGTCCCCTAAATCTAGTGGCGGTCTCGCTGTAGCTTCCTCTGCGTAGTAGCACACCTCGCACTGGGAAACAGCGCGGCCACGCCGTTAAACCCCCAACTTCTGAACGTTTGACCTCGGATCAGGTAGGAATACCCGCTGAACTTAAG

SUB6288079 Seq47        MN450613

>Seq47 [organism=Exophiala sp.] [Exo1] 18S ribosomal RNA gene, partial sequence; internal transcribed spacer 1, 5.8S ribosomal RNA gene, and internal transcribed spacer 2, complete sequence; and 28S ribosomal RNA gene, partial sequence

AGGGATCATTAACGAGTTAGGGTCTCACCGGCCCGACCTCCCAACCCTTTGTCTACTTGACCATCGTTGCTTCGGCGAGCCCGTCCTCATGGACCGCCGGAGGGACCTTGACCGGCCCTCTGGCCCGCGCTCGTCGATGGCCCCCAACTTTAAAATCTTAACTAAACGTGTCTTGAATCTAAGTACTATTGTTAAATAAAAACAAAACTTTCAACAACGGATCTCTTGGTTCTGGCATCGATGAAGAACGCAGCGAAATGCGATAAGTAATGCGAATTGCAGAATTCCAGTGAGTCATCGAATCTTTGAACGCACATTGCGCCCTTTGGTATTCCGAAGGGCATGCCTGTTCGAGCGTCATTATCACCCCTCAAGCCCCCGGCTTGGTGTTGGACGGCTTGGTGGAGACCCTTTTGTGGCCCACCCCTCCCAAAGACAATGACGGCGGCCTCGTTGGACCCCCGGTACACTGAGTTCTTCACGGGACACGTATCGGACACACGGGTTTACGGGCCACGGTCTGCCTCCCCTTAGGGAGAATTTTTCTCAAGGTTGACCTCGGATCAGGTAGGGATACCCGCTGAACTTAA

SUB6288079 Seq48        MN450614

>Seq48 [organism=Exopphiala equina] [Exo2] 18S ribosomal RNA gene, partial sequence; internal transcribed spacer 1, 5.8S ribosomal RNA gene, and internal transcribed spacer 2, complete sequence; and 28S ribosomal RNA gene, partial sequence

ACCTCCCAACCTTTTGTCTACTTGACCATCGTTGCTTCGGCGAGCCCGTCCTCACGGACCGCCGGAGGGATCTTTACTGGCCCTCTGGTCCGCGCTCGTCGGGTAGCCCAACCTTTAAAATCTTTAACTAAACGTGCCTTAATCTAAGTACAATTATTAAATAAAAGCAAAACTTTCAACAACGGATCTCTTGGTTCTGGCATCGATGAAGAACGCAGCGAAATGCGATAAGTAATGCGAATTGCAGAATTCCAGTGAGTCATCGAATCTTTGAACGCACATTGCGCCCTTTGGTATTCCGAAGGGCATGCCTGTTCGAGCGTCATTATCACCCCTCAAGCCCTCGGCTTGGTGTTGGACGGTTTGGTGGAGGCCCCCTCGGGGGCTCCTGCCCCTCCCAAAGACAATGACGGCGGCCTCGTTGGACCCCCGGTACACTGAGTTCTTCAACGGGACACCGTATCGGACACCAGGGAATACGGGAGACGGTCTGCCTCCCCTCATGGAGAATCTTTTAATTGACTCG

SUB6288079 Seq49        MN450615

>Seq49 [organism=Exophiala sp.] [Exo3] 18S ribosomal RNA gene, partial sequence; internal transcribed spacer 1, 5.8S ribosomal RNA gene, and internal transcribed spacer 2, complete sequence; and 28S ribosomal RNA gene, partial sequence

AAGGATCATTAACGAGTTAGGGTCTCTCTGGCCCGACCTCCCAACCCTTTGTCTACTTGACCATCGTTGCTTCGGCGAGCCCGTCCTCACGGACCGCCGGAGGGATCTTTACTGGCCCTCTGGTCCGCGCTCGTCGGTAGCCCAACCTTTAAAATCTTTAACTAAACGTGCCTTAATCTAAGTACAATTATTAAATAAAAGCAAAACTTTCAACAACGGATCTCTTGGTTCTGGCATCGATGAAGAACGCAGCGAAATGCGATAAGTAATGCGAATTGCAGAATTCCAGTGAGTCATCGAATCTTTGAACGCACATTGCGCCCTTTGGTATTCCGAAGGGCATGCCTGTTCGAGCGTCATTATCACCCCTCAAGCCCTCGGCTTGGTGTTGGACGGTTTGGTGGAGGCCCCCTCGGGGGCTCCTGCCCCTCCCAAAGACAATGACGGCGGCCTCGTTGGACCCCCGGTACAGTGAGTTCTTCACGGGACACGTATCGGACACATGGGTTTACGGGACACGGTCTGCCTCCCCTTCAGGGAGAATCTTCTAAGGTGACCTCGCAATAC

SUB6288079 Seq50        MN450616

>Seq50 [organism=Tetracladium sp.] [Tetra1] 18S ribosomal RNA gene, partial sequence; internal transcribed spacer 1, 5.8S ribosomal RNA gene, and internal transcribed spacer 2, complete sequence; and 28S ribosomal RNA gene, partial sequence

AGTATCCACCAGGACCCTATAGCGATGAATTTACTACGCTTAGAGCCTGACAGCACTGCCACTGATTTTAGAGGCTGCCTTTACAGCAGGCCCCAACACCAAGCTAGGCTTGAGGGGTGATAATGACGCTCGAATAGGCATGCCCCTCGGAATACCAAGGGGCGCAATGTGCGTTCAAAGATTCGATGATTCACTGAATTCTGCAATTCACATTACTTATCGCATTTCGCTGCGTTCTTCATCGATGCCAGAGCCAAGAGATCCGTTGTTGAAAGTTTTAAATATTAACTTTTTACTCAGACGACACTAATAATTCAGGGTTTTGGGTCCTCTGGCGGGCACTTACCAGCCGAAGCCAGTAGCTAGGCGGCCCGCCAAAGCAACAAAGGTATAGTATACAAAGGGTGGGAGATCTACCCGTGAGGGCATGAACTCGGTAATGATCCTTCCGCAGGTTCACCTACGGAAACCTTGTTACGACTTTTACTTCCTCT

SUB6288079 Seq51        MN450617

>Seq51 [organism=tetracladium sp.] [Tetra2] 18S ribosomal RNA gene, partial sequence; internal transcribed spacer 1, 5.8S ribosomal RNA gene, and internal transcribed spacer 2, complete sequence; and 28S ribosomal RNA gene, partial sequence

AAGGATCATTACCGAGTTCATGCCCTCACGGGTAGATCTCCCACCCTTTGTATACTATACCTTTGTTGCTTTGGCGGGCCGCCTAGCTACTGGCTTCGGCTGGTAAGTGCCCGCCAGAGGACCCAAAACCCTGAATTATTAGTGTCGTCTGAGTAAAAAGTTAATATTTAAAACTTTCAACAACGGATCTCTTGGCTCTGGCATCGATGAAGAACGCAGCGAAATGCGATAAGTAATGTGAATTGCAGAATTCAGTGAATCATCGAATCTTTGAACGCACATTGCGCCCCTTGGTATTCCGAGGGGCATGCCTATTCGAGCGTCATTATCACCCCTCAAGCCTAGCTTGGTGTTGGGGCCTGCTGTAAAGGCAGCCTCTAAAATCAGTGGCAGTGCTGTCAGGCTCTAAGCGTAGTAAATTCATCGCTATAGGGTCCTGGTGGATACTCGTCAAAACCCCCCATTTTTAATGATTGACCTCGGATTAGGTAGGGATACCCGCTGAACTTAA

SUB6288079 Seq52        MN450618

>Seq52 [organism=Tetracladium sp.] [Tetra3] 18S ribosomal RNA gene, partial sequence; internal transcribed spacer 1, 5.8S ribosomal RNA gene, and internal transcribed spacer 2, complete sequence; and 28S ribosomal RNA gene, partial sequence

CATTTAGAGGAAGTAAAAGTCGTAACAAGGTTTCCGTAGGTGAACCTGCGGAAGGATCATTACCGAGTTCATGCCCTTCGGGGTAGATCTCCCACCCTTTGTATACTATACCTTTGGTTGCTTTGGCGGGCCGCCTAGCTACTGGCTTCGGCTGGTAAGTGCCCGCCAGAGAACCCAAAACCCTGAATTATTAGTGTCGTCTGAGTAAAATATTTAATATTTAAAACTTTCAACAACGGATCTCTTGGCTCTGGCATCGATGAAGAACGCAGCGAAATGCGATAAGTAATGTGAATTGCAGAATTCAGTGAATCATCGAATCTTTGAACGCACATTGCGCCCCTTGGTATTCCGAGGGGCATGCCTATTCGAGCGTCATTATCACCCCTCAAGCCTAGCTTGGTGTTGAGACCTGCTGTCAAGGCAGTCTCTAAAATCAGTGGCAGTGCTGTCAGGCTCTAAGCGTAGTAAATTCATCGCTATAGACACCTGGTGGCCACTCGCCAGAACCACCCCATTTT

SUB6288079 Seq53        MN450619

>Seq53 [organism=Pleosporales sp.] [Pleo1] 18S ribosomal RNA gene, partial sequence; internal transcribed spacer 1, 5.8S ribosomal RNA gene, and internal transcribed spacer 2, complete sequence; and 28S ribosomal RNA gene, partial sequence

AAGGATCATTATCGTAGGGCTTCGGTCCTGTCGAGATAGCGTCCTTGCCTATTTTTTGGAGCACCATTTGTTTCCTCAGCAGGCCTGCCTGCTATTGGGGACCCTTTAAACTCTTTGTAAATACAGTAACTGTCTGAAAAACAAACAAAAAAATCAAAACTTTCAACAATGGATCTCTTGGTTCTGGCATCGATGAAGAACGCAGCAAAATGCGATAAGTAGTGTGAATTGCAGAATTCAGTGAATCATCGAATCTTTGAACGCACATTGCGCCCTTTGGTATTCCTTAGGGCATGCCTGTTCGAGCGTCATTTTCACCTTCAAGCACAGCTTGGTGTTGGGTGTCTGTCCTGCCGTCGGCATGGACTCGCCTTAAAATAATTGGCAGCCACTTCAGCGGCTTTGAGCGCAGTAGAATTGCGTCTCTGGCAGCATAAGGTGGCCTCCATAAGCTTTACTTTTTTATTTTGACCTCGGATCAGGTAGGGATACCCGCTGAACTTAA

SUB6288079 Seq54        MN450620

>Seq54 [organism=Pleosporales sp.] [Pleo2] 18S ribosomal RNA gene, partial sequence; internal transcribed spacer 1, 5.8S ribosomal RNA gene, and internal transcribed spacer 2, complete sequence; and 28S ribosomal RNA gene, partial sequence

AAGGATCATTATCAATTTAACAGTTCGTGGGATGGCGCAATCCATCTCACGCCTGTTTCAACCCTTTGTCTTTTGCGTACTGTATGTTTCCTCGGCGGGTCCGCCCGCCGATAGGACACTTCAAAACCTTTTGTAATTGCAATCAGCGTCAGAAAAACTATAATAGTTACAACTTTCAACAACGGATCTCTTGGTTCTGGCATCGATGAAGAACGCAGCGAAATGCGAAAAGTAGTGTGAATTGCAGAATTCAGTGAATCATCGAATCTTTGAACGCACATTGCGCCCCTTGGTATTCCATGGGGCATGCCTGTTCGAGCGTCATTTGTACCCTCAAGCTCTGCTTGGTGTTGGGTGTTTGTCCCGCCTTGCGCGTGGACTCGCCTTAAAGTCATTGGCAGCCGGAATAATTCTGGGGAACGCAGCACAACTGCAGCCTCCACTTTACGCCGAGCGTCCAGTAAGCCTTTTTTCAACGTTTGACCTCGGATCAGGTAGGGATACCCGCTGAACTTAA

SUB6288079 Seq55        MN450621

>Seq55 [organism=Pleosporales sp.] [Pleo3] 18S ribosomal RNA gene, partial sequence; internal transcribed spacer 1, 5.8S ribosomal RNA gene, and internal transcribed spacer 2, complete sequence; and 28S ribosomal RNA gene, partial sequence

AAGGATCATTACCTTTGGCTGCCGTGAGCGCTTCGGCGTGAGCGACAGTCATTCTATAGTGACGCCTCCGTGTCCGGGAAACCGGCGCGGGGCTGACCTAACCCTTCTCTACGAGTACCTATTATTCTCCTTCGGCGGGGTCAACCCGCCGCTGGAACTTAAGAACCAACTTGCATTTAGCATTACCTGTTCTGATAACAATTAATTATTACAACTTTCAACAATGGATCTCTTGGCTCTGGCATCGATGAAGAACGCAGCGAAATGCGATAAGTAGTGTGAATTGCAGAATTCAGTGAATCATCGAATCTTTGAACGCACATTGCGCCCCATGGTATTCCGTGGGGCATGCCTGTTCGAGCGTCATCTACACCCTCAAGCACTGCTTGGTGTTGGGCGTCTGTCCCGCCTTTGCGCGTGGACTCGCCCCAAAGTCATTGGCAGCGGTCTCTGGCACCTCAACGCGCAGTACAATGCGTTCATTGGGGTGCCGTGGGCGCGACCATAAAGC

SUB6288079 Seq56        MN450622

>Seq56 [organism=Melanconium hedericola] [Melan1] 18S ribosomal RNA gene, partial sequence; internal transcribed spacer 1, 5.8S ribosomal RNA gene, and internal transcribed spacer 2, complete sequence; and 28S ribosomal RNA gene, partial sequence

AGGGATCATTGCTGGAACGCGCTTCGGCGCACCCAGAAACCCTTTGTGAACTTATACCTTACTGTTGCCTCGGCGCAGGCCGGCCTCTCGAGGCCCCTTGGAAACAAGGAGCAGCCCGCCGGCGGCCAACCAAACTCTTGTTTCTTAGTGAATCTCTGAGTAAAAAAACAAAAATGAATCAAAACTTTCAACAACGGATCTCTTGGTTCTGGCATCGATGAAGAACGCAGCGAAATGCGATAAGTAATGTGAATTGCAGAATTCAGTGAATCATCGAATCTTTGAACGCACATTGCGCCCTCTGGTATTCCGGAGGGCATGCCTGTTCGAGCGTCATTTCAACCCTCAAGCAATGCTTGGTGATGGGGCACCGCCTGTAAAAGGGCGGGCCCTGAAATCTAGTGGCGAGCTCGCCAGGACCCCGAGCGTAGTAGTTATATCTCGCTTTGGAAGGCCCTGGCGGTGCCCTGCCGTTAAACCCCCAACTTCTGAAATTTTGACCTCGGATCAGGTAGGAATACCCGCTGAACTTAA

SUB6288079 Seq57        MN450623>Seq57 [organism=Paecilomyces carneus] [Pae1] 18S ribosomal RNA gene, partial sequence; internal transcribed spacer 1, 5.8S ribosomal RNA gene, and internal transcribed spacer 2, complete sequence; and 28S ribosomal RNA gene, partial sequence

AGGGATCATTACCGAGTTTACAACTCCCAAACCCCCTGTGAACTTATACCATTTACTGTTGCTTCGGCGGGTCATGGCCCCGGGGAAGGACAGCGGTCGCCGTCAGGCCTCAGCTGCCCGCCCCCGGAAACAGGCGCCCGCCGGGGAACTCAAACTCTTCTGTATTTCTTTATCTAATATATACTGTCTGAGTAAAAACTAAAATGAATCAAAACTTTCAACAACGGATCTCTTGGTTCTGGCATCGATGAAGAACGCAGCGAAATGCGATAAGTAATGTGAATTGCAGAATTCAGTGAATCATCGAATCTTTGAACGCACATTGCGCCCGCCAGTATTCTGGCGGGCATGCCTGTTCGAGCGTCATTTCAACCCTCAAGTCCCCTGTGGACTCGGTGTTGGGGACCGGCGAGACAGCCGCGGATCTTCTTCCGCAGCGAGTCGCCGCCCCCCAAATGACTTGGCGGCCTCGTCGCGGCCCTCCTCTGCGTAGTATAGCACACCTCGCAACAGGAGCCCGGCGAATGGCCACTGCCGTAAAACCCCCCAACTTTCAGAGTTGACCTCGAATCAGGTAGGAATACCCGCTGAACTTAA

SUB6288079 Seq58        MN450624

>Seq58 [organism=Paecilomyces sp.] [Pae2] 18S ribosomal RNA gene, partial sequence; internal transcribed spacer 1, 5.8S ribosomal RNA gene, and internal transcribed spacer 2, complete sequence; and 28S ribosomal RNA gene, partial sequence

AAGTCGTAACAAGGTCTCCGTTGGTGAACCAGCGGAGGGATCATTACCGAGTTTTCAACTCCCAAACCCACTGTGAACATATACCTTTGTTTTCGTTGCCTCGGCGGTTCGCGCCGCCGGGTGACACCTAAACCCTGATTTTAATTACAGAAGTCTTTCTGAGTAAAAACATTCTAAATGAATCAAAACTTTCAACAACGGATCTCTTGGTTCTGGCATCGATGAAGAACGCAGCGAAATGCGATAAGTAATGTGAATTGCAGAATTCAGTGAATCATCGAATCTTTGAACGCACATTGCGCCCGCCAGTATTCTGGCGGGCATGCCTGTTCGAGCGTCATTTCAACCCTCAAGACCCCTTCGGGGGACTTGGTGTTGGGGACCGGCACAGGGGCCTGCCTGCTTGTCTTGCAGCGCCCTCGCCGCCCCCGAAATGAATTGGCGGCCTCGTCGCGGCCTCCCCTGCGTAGTAGCACAACCTCGCAACGGGAACGTGACGGCGGCCACTGCCGTAAAACAACCCCAATTTTATTAGAGTTGACCTCGAATCAGGTAGGAATACCCGCTGAACTTAA

SUB6288079 Seq59        MN450625

>Seq59 [organism=Ascovirgaria occulta][Asc1] 18S ribosomal RNA gene, partial sequence; internal transcribed spacer 1, 5.8S ribosomal RNA gene, and internal transcribed spacer 2, complete sequence; and 28S ribosomal RNA gene, partial sequence

CATGTGAACATACCTCGTGTTGCCTCGGCGGCGAGCGTAGTGCTCGGGAGGTGCCTACCCGGTACCTACCCTAGAGCTACCCTGGAGCCTCCTACCCTGGAGCTGCGAGGTACCTACCCTGGAGTTACCCGGTAGTAACATACCCGGTAGCTACCCTGGAGGACATGCGCATCCGCCGGAGGACCACTCAAACTCTTGTTTCTGTGTGGCACATCTGAGTAATTATATTTAATAAGTCAAAACTTTCAACAACGGATCTCTTGGTTCTGGCATCGATGAAGAACGCAGCGAAATGCGATAAGTAATGTGAATTGCAGAATTCAGTGAATCATCGAATCTTTGAACGCACATTGCGCCCATTAGTATTCTAGTGGGCATGCCTGTTCGAGCGTCATTAAGTCCATTAAGCCTAGTTGCTTAGCGTTGGGAGCATTACCGTACGGTAACTCCTCAAAGTCAGTGGCAGGGTTACGGTACACTCTAAGCGTAGTAGATTTCTTTCTCGCTTTTGCTGTGGCCGCGGCCGTTGCCATAAACCTATACTTCTTTAGTTGGTGACCTCGGATCAGGT

SUB6288079 Seq60        MN450626

>Seq60 [organism=Pyrenochaeta sp] [Pyren1] 18S ribosomal RNA gene, partial sequence; internal transcribed spacer 1, 5.8S ribosomal RNA gene, and internal transcribed spacer 2, complete sequence; and 28S ribosomal RNA gene, partial sequence

AGGGATCATTACCGAGTTGCAAAACTCCAAACCCATGTGAACATACCTCGTGTTGCCTCGGCGGCGAGCGAAGTGCTCGGGAGGTGCCTACCCGGTACCTACCCTAGAGCTACCCTGTAGCTACCTACCCGGTAGTTGCGAGGTACCTACCCTGTACCTACCCGGTAGCTACCCTGTAGGACTGGCGCATCCGCCGAAGGACCACTTAAACTCTTGTTTCTATGTGGCACGTCTGAGTAATTATATTTAATAAGTCAAAACTTTCAACAACGGATCTCTTGGTTCTGGCATCGATGAAGAACGCAGCGAAATGCGATAAGTAATGTGAATTGCAGAATTCAGTGAATCATCGAATCTTTGAACGCACATTGCGCCCATTAGTATTCTAGTGGGCATGCCTGTTCGAGCGTCATTTAAGTCCATTAAGCCTAGTTGCTTAGCGTTGGGAGCATTACCGTACGGTAACTCCTCAAAGTCAGTGGCAGGGTTGCAGGTGCACTCTTAGCGTAGTAGATTTATTACCGCTTACGATTGTCCCGTAGCCGTCGCCATAAGCCTATATTTTTTATTGGTTGACCTCGGATCAGGTAGGAATACCCGCTGAACTTAA

SUB6288079 Seq61        MN450627

>Seq61 [organism=Cyphellophora sp.] [Cyph1] 18S ribosomal RNA gene, partial sequence; internal transcribed spacer 1, 5.8S ribosomal RNA gene, and internal transcribed spacer 2, complete sequence; and 28S ribosomal RNA gene, partial sequence

AAAGTCGTAACAAGGTTTCCGTAGGTGAACCTGCGGAAGGATCATTACCGAGTTAGGGTGCCTCGTCGCGCCCGACCTCCAACCCTTTGCTTACTTGACCTATTTTGTTGCTTCGGCAGGCCCGCCGCCCGGAAACGGGTGGCCGCCGGGGGCGTTTCACCGCCCCGGGCCCGCGCCTGTCGATGGCCCTATTAAAACTCTTGTCAAAACGTGTCGTCTGAGTTTATCTAAACAAATAAAAACCAAAACTTTCAACAACGGATCTCTTGGTTCTGGCATCGATGAAGAACGCAGCGAAATGCGATAAGTAATGCGAATTGCAGAATCCGTGAGTCATCGAATCTTTGAACGCACATTGCGCCCTCTGGTATTCCGGAGGGCATGCCTGTTCGAGCGTCATTATCACCCCTCAAGCCCGGCTTGTTGTTGGATGCAGCGCTTATCCCGCTCCTCCCAAAGATAATGACGGCGTCTGCGACGACTCCTGTACACTGAGCTTTCGGGCACGTACACGGCTAGAAGTCCAGACCCGGTCGCCGTCCCCCCCGCGGGGACACCCATACCA

SUB6288079 Seq62        MN450628

>Seq62 [organism=Cyphellophora sp.] [Cyph2] 18S ribosomal RNA gene, partial sequence; internal transcribed spacer 1, 5.8S ribosomal RNA gene, and internal transcribed spacer 2, complete sequence; and 28S ribosomal RNA gene, partial sequence

AGGATCATTACCGAGTTAGGGTGCCTAACCCGCGCCCGACCTCCAACCTTTTGTCTACCTGACCTTATGTTGCTTCGGCAGGCCCGCCTGCCCGGACCTCGCGTCTGGGCGGGCCGCCGGGGGTCTCGCGACCCCGGGCCTGTGCCTGTCGATGGCCCACACCAAAACTATTGTTAAAACGTGTCGTCTGAGTTATCTAAACAAATGAAATTAAAACTTTCAACAACGGATCTCTTGGTTCTGGCATCGATGAAGAACGCAGCGAAATGCGATAAGTAATGCGAATTGCAGAATCCGTGAGTCATCGAATCTTTGAACGCACATTGCGCCCTCTGGTATTCCGGAGGGCATGCCTGTTCGAGCGTCATTATCACCCCTCAAGCCCGGCTTGTTGTTGGTCGCAGCGGGAGGCTCGCCCCCCCGCTCTTCCCAAAGATAATGACGGCGTCTGTGAGGACTCCTGTACACTGAGCTTTCGGGCACGTACTAGGCTAGAAGTCCAGACCCGGTCACCGTCCCCTTACAGGGACACTTTTCTTTTTACAAGGTTGACCTCGGATCAGGTAGGGATACCCGCTGAACTTAA

SUB6288079 Seq63        MN450629

>Seq63 [organism=Dothideomycetes sp.] [Doth1] 18S ribosomal RNA gene, partial sequence; internal transcribed spacer 1, 5.8S ribosomal RNA gene, and internal transcribed spacer 2, complete sequence; and 28S ribosomal RNA gene, partial sequence

CGGACTATTTGCCACCGGCCTAGGCTGGATAGCGTCCGCCAGAGGATTTTTAAATTCCTTTGAAGGTGAATTCCGAGTCTTTGAAAATTGAATCAAAACTTTCAACAACGGATCTCTTGGTTCTGGCATCGATGAAGAACGCAGCGAAATGCGATAAGTAATGTGAATTGCAGAATTCAGTGAATCATCGAATCTTTGAACGCACATTGCGCCCTTTGGTATTCCGAGGGGCACGCCTGTTCGAGCGTCATTTAAACCATCAAGCCCAGCTTGGTCTTGGACGCGGTCGAGTGACCCGTCCGTAACCCGTTGGCAGTGCAGTCCGGCTTCAAGCGTAGCAAAAATTTCGCTTCAGGAGTCCGTGGCGGCGCCCGCCAGGTAATCACATCCTAAAGTTTGACCTCGGATCAGGTGAGGATACCCGCTGAACTTAA

**SUB6288079 Seq64        MN450630**

>**Seq64 [organism=Ceratobasidium albasitensis] [Cerbium1] 18S ribosomal RNA gene, partial sequence; internal transcribed spacer 1, 5.8S ribosomal RNA gene, and internal transcribed spacer 2, complete sequence; and 28S ribosomal RNA gene, partial sequence**

AAGGATCATTATTGAATGAATATAGAGTTGGTTGTCGCTGGCTCCTCCGGGAGCATGTGCACGCTTTCTCTTTCATCCACACACACCTGTGCACTTGTGAGACGGAGGACCGTAAAAAAGTCTTCCGTCTATTAAACCACACAAACCCCATTGTATTTAAATTGAATGTAATTGATGTAACGCATCATTAGAACTAAGTTTCAACAACGGATCTCTTGGCTCTCGCATCGATGAAGAACGCAGCGAAATGCGATAAGTAATGTGAATTGCAGAATTCAGTGAATCATCGAATCTTTGAACGCACCTTGCGCTCCTTGGTATTCCTTGGAGCATGCCTGTTTGAGTATCATGAAATTCTCAAAGTAAATCTTTTGTTAATTCAACTGGTTTGCTTTGGATTTGGAGGTTTTGCAGATTTCACAGTCTGCTCCTCTTAAATAAATTAGCTGGATCTCAGTATATGCTTGGTTCCACTCGGCGTGATAAGTATCACTCGCTGAGGACACTGTAAAAGGTGGCCAGGAAATGCAAATGAACC

**SUB6288079 Seq65        MN450631**

**>Seq65 [organism=Ceratobasidium sp.] [Cerbium2] 18S ribosomal RNA gene, partial sequence; internal transcribed spacer 1, 5.8S ribosomal RNA gene, and internal transcribed spacer 2, complete sequence; and 28S ribosomal RNA gene, partial sequence**

AAGTCGTAACAAGGTTTCCGTAGGTGACTGCGGAAGGATCATTATTGAATTTAATGAAGAGCTTGGTTGTAGCTGGCCCAATTGATTTTTTTCAATTAAAATTTCAATTTGGGCATGTGCACACCTTTCTCTTTCATCCACACACACCTGTGCACCTGTGAGGCAGATTAGGGGAACTTTCATTTCTTTGAGGGGGGGGGACCCTGCTGTCTACTTAATTTATATAAACTCAATTTATTTTAAAATGAATGTAAAGGATGTAACACATCTAATACTAAGTTTCAACAACGGATCTCTTGGCTCTTGCATCGATGAAGAACGCAGCGAAATGCGATAAGTAATGTGAATTGCAGAATTCAGTGAATCATCGAATCTTTGAACGCACCTTGCGCTCCTTGGTATTCCTTGGAGCATGCCTGTTTGAGTATCGTGAAATCTTCAAAGTAAATCTTTTGTTCACTCAATTGATTCTACTTTGGTAATGGAGGTTTACTGCAGCTTCACATCTGCTCCTCTTTGTGCATTAGCTGGATTCTCAGTGTTATGCTTGGTTCCACTCAGCGTGATAAGTTAATTAATTCGCTGAGGACACTCCATAACCAAAGGGGTGGCCAAGGTAAATACAGATGAACCCGCTTCTAATAGTCCATTGGTTGGACAAAATAAATAAATAAAT

SUB6288079 Seq66        MN450632

>Seq66 [organism=Hypocrea koningii] [Crea1] 18S ribosomal RNA gene, partial sequence; internal transcribed spacer 1, 5.8S ribosomal RNA gene, and internal transcribed spacer 2, complete sequence; and 28S ribosomal RNA gene, partial sequence

CGGAGGGATCATTACCGAGTTTACAACTCCCAAACCCAATGTGAACCATACCAAACTGTTGCCTCGGCGGGGTCACGCCCCGGGTGCGTCGCAGCCCCGGAACCAGGCGCCCGCCGGAGGGACCAACCAAACTCTTTCTGTAGTCCCCTCGCGGACGTTATTTCTTACAGCTCTGAGCAAAAATTCAAAATGAATCAAAACTTTCAACAACGGATCTCTTGGTTCTGGCATCGATGAAGAACGCAGCGAAATGCGATAAGTAATGTGAATTGCAGAATTCAGTGAATCATCGAATCTTTGAACGCACATTGCGCCCGCCAGTATTCTGGCGGGCATGCCTGTCCGAGCGTCATTTCAACCCTCGAACCCCTCCGGGGGTCCGGCGTTGGGGATCGGGAACCCCTAAGACGGGATCCCGGCCCCGAAATACAGTGGCGGTCTCGCCGCAGCCTCTCATGCGCAGTAGTTTGCACAACTCGCACCGGGAGCGCGGCGCGTCCACGTCCGTAAAACACCCAACTTCTGAAATGTTGACCTCGGATCAGGT

SUB6288079 Seq67        MN450633

>Seq67 [organism=Hypocrea lixii] [Crea2] 18S ribosomal RNA gene, partial sequence; internal transcribed spacer 1, 5.8S ribosomal RNA gene, and internal transcribed spacer 2, complete sequence; and 28S ribosomal RNA gene, partial sequence

AACAGCGGAGGGATCATTACCGAGTTTTACAACTCCCAAACCCAATGTGAACGCTACCAAACTGGTTGCCTCGGCGGGATCTCTGCCCCGGGTGCGTCGCAGCCCCGGACCAAGGCGCCCGCCGGAGGACCAACCAAAACTCTTATTGTATACCCCCTCGCGGGTTTTTTTTTATAATCTGAGCCTTCTCGGCGCCTCTCGTAGGCGTTTCGAAAATGAATCAAAACTTTCAACAACGGATCTCTTGGTTCTGGCATCGATGAAGAACGCAGCGAAATGCGATAAGTAATGTGAATTGCAGAATTCAGTGAATCATCGAATCTTTGAACGCACATTGCGCCCGCCAGTATTCTGGCGGGCATGCCTGTCCGAGCGTCATTTCAACCCTCGAACCCCTCCGGGGGGTCGGCGTTGGGGATCGGCCCTGCCTTGGCGGTGGCCGTCTCCGAAATACAGTGGCGGTCTCGCCGCAGCCTCTCCTGCGCAGTAGTTTGCACACTCGCATCGGGAGCGCGGCGCGTCCACAGCCGTTAAACACCCAACTTCTGAAATGTTGACCTCGGATCAGGTAGGAATACCCGCTGAACTTAA

SUB6288079 Seq68        MN450634

>Seq68 [organsim=Fusarium merismoides] [Fus1] 18S ribosomal RNA gene, partial sequence; internal transcribed spacer 1, 5.8S ribosomal RNA gene, and internal transcribed spacer 2, complete sequence; and 28S ribosomal RNA gene, partial sequence

AAGTCGTAACAAGGTCTCCGTTGGTGAACCAGCGGAGGGATCATTACCGAGTTTACAACTCCCAAACCCCTGTGAACATACCTATCGTTGCTTCGGCGGATCCGCCCCGGTGCCTTCGGGCCCGGACTCAGGCGCCCGCCGGGGGACCCAAACTCTTGTATTTCTTTAGTATCTTCTGAGTAAACAAGCAAATAAATTAAAACTTTCAACAACGGATCTCTTGGTTCTGGCATCGATGAAGAACGCAGCGAAATGCGATAAGTAATGTGAATTGCAGAATTCCGTGAATCATCGAATCTTTGAACGCACATTGCGCCCGCCAGTATTCTGGCGGGCATGCCTGTTCGAGCGTCATTTCAACCCTCAAGCCCCCGGGCTTGGTGTTGGGGCTCGGCCCGTCCCTAGCGGCGCGCCGTCCCCGAAATCTAGTGGCGGTCACGCTGTACTCCTCTGCGTAGTAATCTAACCTCGCAACGGGACAAAGCGCGGCCACGCCGTTAAACCCCCAACTTCTGAAGGTTGACCTCGGATCAGGTAGGAATACCCGCTGAACTTAA

SUB6288079 Seq69        MN450635

>Seq69 [organism=Clonostachys rosea] [Clono1] 18S ribosomal RNA gene, partial sequence; internal transcribed spacer 1, 5.8S ribosomal RNA gene, and internal transcribed spacer 2, complete sequence; and 28S ribosomal RNA gene, partial sequence

GGCAGGGGCTCATCGCTCTCCGATGCGGAATATCACTACTTCGCAGAGGAGGCCACGACGGGTCCGCCACTAGATTTAGGGGCCGGCCGTCCCTCGCGGGCTTTGGCCGATCCCCAACACCACACCCTAGGGGCATGAGGGTTGAAATGACGCTCAGACAGGCATGCCCGCCAGAATACTGGCGGGCGCAATGTGCGTTCAAAGATTCGATGATTCACTGAATTCTGCAATTCACATTACTTATCGCATTTCGCTGCGTTCTTCATCGATGCCAGAACCAAGAGATCCGTTGTTGAAAGTTTTTATTTATTTGTAAAAACTACTCAGAAGATTCCAAAATAAAACAAGAGTTGAGTTTCCTAGGCGGGCGCCTGATCCGGGGCACACGAGGCGCCCGGGGCAATCCCGCCGAAGCAACAATAGGTATGTTCACATGGGTTTGGGAGTTGTAAACTCGGTAATGATCCCTCCGCTGGTTCACCAACGGAGACCTTGTTACGACTTTTACTTCCTCTAAATGGACCAA

SUB6288079 Seq70        MN450636

>Seq70 [organism=Unidentified root fungus] [Unid1] 18S ribosomal RNA gene, partial sequence; internal transcribed spacer 1, 5.8S ribosomal RNA gene, and internal transcribed spacer 2, complete sequence; and 28S ribosomal RNA gene, partial sequence

AAAGTCGTAACAAGGTTTCCGTAGGTGAACCTGCGGAAGGATCATTATCGTAGGGCTTCGGTCCTGTCGAGATAGCGTCCTTGCCTATTTTTTGGAGCACCATTTGTTTCCTCAGCAGGCCTGCCTGCTATTGGGGACCCTTTAAACTCTTTGTAAATACAGTAACTGTCTGAAAAACAAACAAAAAAATCAAAACTTTCAACAATGGATCTCTTGGTTCTGGCATCGATGAAGAACGCAGCAAAATGCGATAAGTAGTGTGAATTGCAGAATTCAGTGAATCATCGAATCTTTGAACGCACATTGCGCCCTTTGGTATTCCTTAGGGCATGCCTGTTCGAGCGTCATTTTCACCTTCAAGCACAGCTTGGTGTTGGGTGTCTGTCCTGCCGGCGGCATGGACTCGCCTTAAAATAATTGGCAGCCACTTCAGCGGCTTTGAGCGCAGTAGAGTTGCGTCTCTGGCAGCATAAGGTGGCCTCCCATAAGCTTTACCTCTTTATTTTGACCTCGGATCAGGTAGGGATACCCGCTGAACTTAA

SUB6288079 Seq71        MN450637

>Seq71 [organism=Unidentified root fungus] [Unid2] 18S ribosomal RNA gene, partial sequence; internal transcribed spacer 1, 5.8S ribosomal RNA gene, and internal transcribed spacer 2, complete sequence; and 28S ribosomal RNA gene, partial sequence

AGGGATCATTACTGAGTGTAAAAACTCCCAAACCCCTGTGAACATACCATCTGTTGCTTCGGCGGGATCGCCCCGGGCGCCTTTGCGTGCCCCGGATCCAGGCGCCCGCCGGAGGACTCCAAACTCTTGTTTTATATGTGGCATTATCTGAGTGGCTTATAGCAAAATAAATCAAAACTTTCAACAATGGATCTCTTGGCTCTGGCATCGATGAAGAACGCAGCGAAATGCGATAAGTAATGCGAATTGCAGAATTCAGTGAATCATCGAATCTTTGAACGCACATTGCGCCCGCCAGTATTCTGGCGGGCATGCCTGTCTGAGCGTCAGTTCAACCCTCGCAACCGGCTTTGCTGGATGTGGTGTTGGGGATCGGCTGCCTTCTAGGTGGCCGGCCCCGAAATAGAGTGGCGGTCTCGTCGTGGCCTCCTCTGCGTAGTAGCAATATCTCGCAGGCGGAGAGCGGCGTGGCTATGCCGTAAAACACCCAACTCTTCTAAGGTTGACCTCAGATCAGGTAGGAATACCCGCTGAACTTAA

SUB6288079 Seq72        MN450638

>Seq72 [organism=Unidentified root fungus] [Unid3] 18S ribosomal RNA gene, partial sequence; internal transcribed spacer 1, 5.8S ribosomal RNA gene, and internal transcribed spacer 2, complete sequence; and 28S ribosomal RNA gene, partial sequence

AAGTCGTAACAAGGTCTCCGTTGGTGAACCAGCGGAGGGATCATTACCGAGTTTTCAACTCCCAAACCCACTGTGAACATATACCTTTGTTTTCGTTGCCTCGGCGGTTCGCGCCGCCGGGTGACACCTAAACCCTGATTTTAATTACAGAAGTCTTTCTGAGTAAAAACATTCTAAATGAATCAAAACTTTCAACAACGGATCTCTTGGTTCTGGCATCGATGAAGAACGCAGCGAAATGCGATAAGTAATGTGAATTGCAGAATTCAGTGAATCATCGAATCTTTGAACGCACATTGCGCCCGCCAGTATTCTGGCGGGCATGCCTGTTCGAGCGTCATTTCAACCCTCAAGACCCCTTCGGGGGACTTGGTGTTGGGGACCGGCACAGGGGCCTGCCTGCTTGTCTTGCAGCGCCCTCGCCGCCCCCGAAATGAATTGGCGGCCTCGTCGCGGCCTCCCCTGCGTAGTAGCACAACCTCGCAACGGAACGTGACGGCGGCCACTGCCGTAAACAACCCCAATTTATTAGAGTTGACCTCGAATCAGGTAGGAATACCCGCTGAACTTAA

SUB6288079 Seq73        MN450639

>Seq73 [organism=Unidentified root fungus] [Unid4] 18S ribosomal RNA gene, partial sequence; internal transcribed spacer 1, 5.8S ribosomal RNA gene, and internal transcribed spacer 2, complete sequence; and 28S ribosomal RNA gene, partial sequence

AGGGATCATTACCGAGTTTACAACTCCCAAACCCATGTGAACCTTACCTATCGTTGCTTCGGCGGATCGCCCCGGCGCCTCTGTGCCCGGAGTCAGGCGCCCGCCGGAGGCCCAAACTCTTGCTTTACCTATGGTATTATCTGAGTGGCGTAAGCAAATGAATCAAAACTTTCAACAACGGATCTCTTGGTTCTGGCATCGATGAAGAACGCAGCGAAATGCGATAAGTAATGTGAATTGCAGAATTCAGTGAATCATCGAATCTTTGAACGCACATTGCGCCCGCTAGTACTCTAGCGGGCATGCCTGTTCGAGCGTCATTTCAACCCTCAAGCCCCCCGGGCTTGGCGTTGGGGACCGGCAGCCCGCCCTCTGGCGGCAGCCGCCCCCGAAAACCAGTGGCGGTCTCGCTGTAGCTTCCCCTGCGTAGTAGCACACCTCGCACCGGATCGCGGCGCGGCCATGCCGTAAAACACCCACTTCTGAAGGTTGACCTCGGATCAGGTAGGAATACCCGCTGAACTTAA

SUB6288079 Seq74        MN450640

>Seq74 [organism=Phomopsis sp.] [Phom1] 18S ribosomal RNA gene, partial sequence; internal transcribed spacer 1, 5.8S ribosomal RNA gene, and internal transcribed spacer 2, complete sequence; and 28S ribosomal RNA gene, partial sequence

AAGTCGTAACAAGGTCTCCGTTGGTGAACCAGCGGAGGGATCATTGCTGGAACGCGCTTCGGCGCACCCAGAAACCCTTTGTGAACTTATACCTTACTGTTGCCTCGGCGCAGGCCGGCCTCACCGAGGCCCCTCGGAAACGAGGAGCAGCCCGCCGGCGGCCAACCAAACTCTTGTTTCTTAGTGAATCTCTGAGTAAAAAAAACATAAATGAATCAAAACTTTCAACAACGGATCTCTTGGTTCTGGCATCGATGAAGAACGCAGCGAAATGCGATAAGTAATGTGAATTGCAGAATTCAGTGAATCATCGAATCTTTGAACGCACATTGCGCCCTCTGGTATTCCGGAGGGCATGCCTGTTCGAGCGTCATTTCAACCCTCAAGCATTGCTTGGTGTTGGGGCACCGCCTGTAAAAGGGCGGGCCCTGAAATCTAGTGGCGAGCTCGCCAGGACCCCGAGCGTAGTAATTATATTTCGTTCTGGAAGGCCCTGGCGGTGCCCTGCCGTTAAACCCCCAACTTCTGAAAATTTGACCTCGGATCAGGTAGGAATACCCGCTGAACTTAA

SUB6288079 Seq75        MN450641

>Seq75 [organsim=Trichoderma sp.] [Trich1] 18S ribosomal RNA gene, partial sequence; internal transcribed spacer 1, 5.8S ribosomal RNA gene, and internal transcribed spacer 2, complete sequence; and 28S ribosomal RNA gene, partial sequence

AAGTCGTAACAAGGTCTCCGTTGGTGAACCAGCGGAGGGATCATTACCGAGTTTACAACTCCCAAACCCAATGTGAACGTTACCAAACTGTTGCCTCGGCGGGATCTCTGCCCCGGGTGCGTCGCAGCCCCGGACCAAGGCGCCCGCCGGAGGACCAACCAAAACTCTTATTGTATACCCCCTCGCGGGTTTTTTTTTATAATCTGAGCCTTCTCGGCGCCTCTCGTAGGCGTTTCGAAAATGAATCAAAACTTTCAACAACGGATCTCTTGGTTCTGGCATCGATGAAGAACGCAGCGAAATGCGATAAGTAATGTGAATTGCAGAATTCAGTGAATCATCGAATCTTTGAACGCACATTGCGCCCGCCAGTATTCTGGCGGGCATGCCTGTCCGAGCGTCATTTCAACCCTCGAACCCCTCCGGGGGGTCGGCGTTGGGGATCGGCCCTGCCTTGGCGGTGGCCGTCTCCGAAATACAGTGGCGGTCTCGCCGCAGCCTCTCCTGCGCAGTAGTTTGCACACTCGCATCGGGAGCGCGGCGCGTCCACAGCCGTTAAACACCCAACTTCTGAAATGTTGACCTCGGATCAGGTAGGAATACCCGCTGAAATTAA

SUB6288079 Seq76        MN450642

>Seq76 [organism=Trichoderma sp.] [Trich2] 18S ribosomal RNA gene, partial sequence; internal transcribed spacer 1, 5.8S ribosomal RNA gene, and internal transcribed spacer 2, complete sequence; and 28S ribosomal RNA gene, partial sequence

AAGTCGTAACAAGGTCTCCGTTGGTGAACCAGCGGAGGGATCATTACCGAGTTTACAACTCCCAAACCCCTATGTGAACGTTACCAAAATGTTGCCTCGGCGGGGAATTTATTCATGCCCCGGGCGCGTCGCAGCCCCGGACCAAGGCGCCCGCCGGAGGACCAACCAAAACTCTTTTGTATGTCCCCTCGCGGACTTTTATAATTCTGAACCATCTCGGCGCCCCTAGCGGGCGTTTCGAAAATGAATCAAAACTTTCAACAACGGATCTCTTGGTTCTGGCATCGATGAAGAACGCAGCGAAATGCGATAAGTAATGTGAATTGCAGAATTCAGTGAATCATCGAATCTTTGAACGCACATTGCGCCCGCCAGTATTCTGGCGGGCATGCCTGTCCGAGCGTCATTTCAACCCTCGAACCCCTCCGGGGGTACGGCGTTGGGGATCGGCCCTTTACGGGGCCGGCCCCGAAATACAGTGGCGGTCTCGCCGCAGCCTCTCCTGCGCAGTAGTTTGCACACTCGCATCGGGAGCGCGGCGCGTCCACGTCCGTAAAACACCCAACTTCTGAAATGTTGACCTCGGATCAGGTAGGAATACCCGCTGAACTTAA

SUB6288079 Seq77        MN450643
>Seq77 [organism=Mycochaetophora sp.] [Myco1] 18S ribosomal RNA gene, partial sequence; internal transcribed spacer 1, 5.8S ribosomal RNA gene, and internal transcribed spacer 2, complete sequence; and 28S ribosomal RNA gene, partial sequence

CTTAAAATTAGGGGTTGCTGGCAAGTAGACCTACCGGACTCAATCGCGAGGAGTATTACTACGCGTAGAGCCGACAGGCACCGCCACTGATTTTAGGGGCCGCGAAACCGCGAACCCCAATACCAAGCGAGAGCTTGAGTGGTTATAATGACGCTCGAACAGGCATGCCCCCCGGAATACCAGAGGGCGCAATGTGCGTTCAAAGATTCGATGATTCACTGAATTCTGCAATTCACATTACTTATCGCATTTCGCTGCGTTCTTCATCGATGCCAGAACCAAGAGATCCGTTGTTGAAAGTTTTAACTATTATATAGTACTCAGACATCACTAAAAACAAGAGTTGTGGTCCTCTGGCGGGCACTCAACAGCCGAAGCCGCTGGCACAAGGCGGCCCGCCAAAGCAACAAAGGTAGTTTATTCAAGGGTGGAGTTCAGGACCGAGCTTCTCCGAGAGGCCCGACGACTCTAAACCCTACCGGAGTAGGGTAGCCCCGGGAGCGAGCTCCGCGGGTGCTGTCTATCCTTTGCTCTAGTAATGATCCTTCCGCAGGTTCACCTACGGACCTTTACCCCTGGCCTTTCGATCAGGGCTCGACTATATCTTTAAGTAGC

SUB6288079 Seq78        MN450644
>Seq78 [organism=Collarina aurantiaca] [colla1] 18S ribosomal RNA gene, partial sequence; internal transcribed spacer 1, 5.8S ribosomal RNA gene, and internal transcribed spacer 2, complete sequence; and 28S ribosomal RNA gene, partial sequence

AAGTCGTAACAAGGTCTCCGTTGGTGAACCAGCGGAGGGATCATTACTGAGTGTTACAACTCCCAAACCCTTTGTGAACACTACCATTCGTTGCTTCGGCGGGACCGCCCCGGCGCCTCTGCGCGCCGGAACCAGGCGCCCGCCGGGGGCCCGAAACTCTTGTTTTGTCAGTGGTATTTTCTCTGAGTGGCATAAGCAAATAAATTAAAACTTTCAACAACGGATCTCTTGGTTCTGGCATCGATGAAGAACGCAGCGAAATGCGATAAGTAATGTGAATTGCAGAATTCAGTGAATCATCGAATCTTTGAACGCACATTGCGCCCGCCAGTACTCTGGCGGGCATGCCTGTTCGAGCGTCATTTCAACCCTCGGGCCCCGCATTCGTGCGGGCCCCGGCGTTGGGGACCGGCCTCCGCATCGGACGGCCGCCCCCGAAACCCAGTGGCGGTCCCGCCGCGGCCTTCTCTGCGTAGTAGCATACACCTCGCACTGGAGAGCGGCGCGGCCACGCCGTAAAAGAAACGAACTTTCTGAAGGTTGACCTCGGATCAGGTAGGAATACCCGCTGAACTTAA

SUB6288079 Seq79        MN450645
>Seq79 [organism=Ascomycota sp.] [Mycota1] 18S ribosomal RNA gene, partial sequence; internal transcribed spacer 1, 5.8S ribosomal RNA gene, and internal transcribed spacer 2, complete sequence; and 28S ribosomal RNA gene, partial sequence

AAGTCGTAACAAGGTTTCCGTAGGTGAACCTGCGGAAGGATCATTACCTAGAGTTTGCGGGCTTTGCCTGCTATCTCTTACCCATGTCTTTTGAGTACTTACGTTTCCTCGGTGGGTTCGCCCGCCGATTGGACAATTTAAACCCTTTGCAGTTGCAATCAGCGTCTGAAAAAAATTAATAATTACAACTTTCAACAACGGATCTCTTGGTTCTGGCATCGATGAAGAACGCAGCGAAATGCGATAAGTAGTGTGAATTGCAGAATTCAGTGAATCATCGAATCTTTGAACGCACATTGCGCCCCTTGGTATTCCATGGGGCATGCCTGTTCGAGCGTCATTTGTACCTTCAAGCTCTGCTTGGTGTTGGGTGTTTGTCTCGCCTTTGCGTGTAAACTCGCCTCAAAACAATTGGCAGCCGGCGTATTGATTTCGGAGCGCAGTACATCTCGCGCTTTGCACTCATAACGACGACGTCCAAAAGACATTTT

SUB6288079 Seq80        MN450646
>Seq80 [organism=Ascomycota sp.] [Mycota2] 18S ribosomal RNA gene, partial sequence; internal transcribed spacer 1, 5.8S ribosomal RNA gene, and internal transcribed spacer 2, complete sequence; and 28S ribosomal RNA gene, partial sequence

AGGGATCATTACAGAGTTGCAAAACTCCCAAACCATTGTGAACGTTACCTATCCCGTTGCTTCGGCGGGCGGCCCGGGCCCCGTGCCCGGCGCCCCCCGGCCCCTCGCGGGCGCCCGCCGGAGGTAAACCAAACTCTTGAATTGTATGGCCTCTCTGAGTCTTCTGTACTGAATAAGTCAAAACTTTCAACAACGGATCTCTTGGTTCTGGCATCGATGAAGAACGCAGCGAAATGCGATAAGTAATGTGAATTGCAGAATTCAGTGAATCATCGAATCTTTGAACGCACATTGCGCCCGCCAGTATTCTGGCGGGCATGCCTGTTCGAGCGTCATTTCAACCATCAAGCCCCCGGCTTGTGTTGGGGACCTGCGGCTGCCGCAGGCCCTGAAAACCAGTGGCGGGCTCGCTAGTCACTCCGAGCGTAGTAATACATCTCGCTCAGGGCGTGCTGCGGGTTCCGGCCGTTAAAACCCTTATCGAACCCAAGGTTGACCTCGGATCAGGTAGGAAGACCCGCTGAACTTAA

SUB6288079 Seq81        MN450647
>Seq81 [organism=Neonectria radicola] [Neonec1] 18S ribosomal RNA gene, partial sequence; internal transcribed spacer 1, 5.8S ribosomal RNA gene, and internal transcribed spacer 2, complete sequence; and 28S ribosomal RNA gene, partial sequence

GGGATTATTACTGAGTTTACAAATCCCAAAACCCGTGTGAACATACCATTTGTTGCCTAGGCGGTGCATGCTTAGGCAGCCCGCCAGAGGACCCAAACCCTTGATTTTATACAGTATCTTCTGAGTAAATGATTAAATAAATCAAAACTTTCAACAACGGATCTCTTGGTTCTGGCATCGATGAAGAACGCAGCGAAATGCGATAAGTAATGTGAATTGCAGAATTCAGTGAATCATCGAATCTTTGAACGCACATTGCGCCCGCCAGTATTCTGGCGGGCATGCCTGTTCGAGCGTCATTTCAACCCTCAAGCCCCCGGGCTTGGTGTTGGAGATCGGCGTGCCCCCCGGGGCGCGCCGGCTCCCAAATATAGTGGCGGTCTCGCTGTAGCTTCCTCTGCGTAGTAGCACACCTCGCACTGGAAAACAGCGTGGCCACGCCGTTAAACCCCCCACTTCTGAAAGGTTGACCTCGGATCAGGTAGGAATACCCGCTGAACTTAA

SUB6288079 Seq82        MN450648
>Seq82 [organism=Trichosporon sp.] [Sporon1] 18S ribosomal RNA gene, partial sequence; internal transcribed spacer 1, 5.8S ribosomal RNA gene, and internal transcribed spacer 2, complete sequence; and 28S ribosomal RNA gene, partial sequence

AAGTCGTAACAAGGTTTCCGTAGGTGAACCTGCGGAAGGATCATTAGTGATTGCCATCTTGGCTTAAACTATATCCATCTACACCTGTGAACCGTTTGATTGAATCTTCTGATTCAATTTTACAAACATTGTGTAATGAACGTCATTAGATCATAACAAAAAAAAACTTTTAACAACGGATCTCTTGGCTCTCGCATCGATGAAGAACGCAGCGAAATGCGATAAGTAATGTGAATTGCAGAATTCAGTGAATCATCGAATCTTTGAACGCAACTTGCGCTCTCTGGTATTCCGGAGAGCATGCCTGTTTGAGTGTCATGAAATCTCAACCATTAGGGTTTCTTAATGGCTTGGATTTGGAGGTTGCCATTCTAAATGGCTCCTCTTAAAGGAGTTAGCAAGTTTTACTATTGCTATCTGGCGTAATAAGTTTCGCTGGAATGGTATTGTGAAGCGTGCTTCTAATCGTCTTCGGACAATTACTTTGACTCTGGCCTCAAATCAGTGTAGGACTACCCGCTGAACTTAA

SUB6288079 Seq83        MN450649

>Seq83 [organism=Oidiodendron flavum] [Oi1] 18S ribosomal RNA gene, partial sequence; internal transcribed spacer 1, 5.8S ribosomal RNA gene, and internal transcribed spacer 2, complete sequence; and 28S ribosomal RNA gene, partial sequence

AAGTCGTAACAAGGTCTCCGTAGGTGAACCTGCGGAGGGATCATTACAGAGTTCTCGCCCTCGCGGGTAGATCTCCCACCCACTGTAATTGCTACCGTGTTGCTTTGGCGGGCCGCCGGGCCCAGCCCGCCGCCGGCCCCGGCCGGCGTGTGCCCGCCAGAAGCCCCGCAAACTCTGAATGTCAGCGTCGTCTGAGTACTATATAATAGTTAAAACTTTCAACAACGGATCTCTTGGTTCTGGCATCGATGAAGAACGCAGCGAAATGCGATAAGTAATGCGAATTGCAGAATTCAGTGAGTCATCGAATCTTTGAACGCACATTGCGCCCTGTGGTATTCCGCAGGGCATGCCTGTTCGAGCGTCATTTCAACCCTCAAGCTCTGCTTGGTGTTGGGCCCTGCCCGCTGCGGCCGGCCCTAAAGACAGTGGCGGTGCCGCCTGGCCCTGAGCGTAGTACATCTCTCGCTCCAGTGCCCGGTGGTAGCCTGCCAGAACCCCAACTCTCGTGGTTGACCTCGGATCAGGTAGGGATACCCGCTGAACTTAA

SUB6288079 Seq84        MN450650
>Seq84 [organism=Chrysosporium keratinophilum] [Chry] 18S ribosomal RNA gene, partial sequence; internal transcribed spacer 1, 5.8S ribosomal RNA gene, and internal transcribed spacer 2, complete sequence; and 28S ribosomal RNA gene, partial sequence

AAGTCGTAACAAGGTTTCCGTAGGTGAACCTGCGGAAGGATCATTAAAGTGTTTCGGAGCCTGGCTCAGGCACCTCAACTCGAGGTGTCGGTGCCAGCGCCCCCACACGTGTTTACTCAACTTGGTTGCCTTGGTGAGCCTGCCCTTGTGGCTGCCGGGGATGCCTCACGGCGTCCTGGGCTCGTGCTCACCAATGGAACATTTGAACTCTTATGTGAAAATAGTCAGTCTGAGCATTATGCAAATTAAATAAAACTTTCAACAACGGATCTCTTGGTTCCGGCATCGATGAAGAACGCAGCGAAATGCGATAAGTAATGTGAATTGCAGAATTCCGTGAATCATCGAATCTTTGAACGCACATTGCGCCCTCTGGTATTCCGGGGGGCATGCCTGTTCGAGCGTCATTGCAAACCCCTCAAGCACGGCTTGTGTGTTGGGCCATCGTCCCCTATGGACGGGCCTGAAATGCAGTGGCAGCACCGAGTCCTGGTGTCTGAGTGTATGGGAATCTCTATCGCTCAAAGACCCAATCGGCGCTGATGTCAGATTTTTATCCAGTTTGACCTCGGATCAGGTAGGAGTACCCGCTGAACTTAA

SUB6288079 Seq85        MN450651
>Seq85 [organism= Humicola sp.] [Humi1] 18S ribosomal RNA gene, partial sequence; internal transcribed spacer 1, 5.8S ribosomal RNA gene, and internal transcribed spacer 2, complete sequence; and 28S ribosomal RNA gene, partial sequence

AGGGATCATTACAGAGTTGCAAAACTCCCAAACCATTGTGAACGTTACCAAAACCGTTGCTTCGGCGGGCGGCCCGGGTCACTCCCGGCGCCCCCCGGCCCTCGCGGGCGCCCGCCGGAGGTAAACCAAACTATTGAATTGTATGGCCTCTCTGAGTCTTCTGTACTGAATAAGTCAAAACTTTCAACAACGGATCTCTTGGTTCTGGCATCGATGAAGAACGCAGCGAAATGCGATAAGTAATGTGAATTGCAGAATTCAGTGAATCATCGAATCTTTGAACGCACATTGCGCCCGCCAGTATTCTGGCGGGCATGCCTGTTCGAGCGTCATTTCAACCATCAAGCCCCGGGCTTGTGTTGGGGACCTGCGGCTGCCGCAGGCCCTGAAATGCAGTGGCGGGCTCGCTGTCACTCCGAGCGTAGTAATACATCTCGCTCTGGACGTGCTGCGGGTTCCGGCCGTTAAAAGCCTTATTTACCCAAGGTTGACCTCGGATCAGGTAGGAAGACCCGCTGAACTTAA

SUB6288079 Seq86        MN450652
>Seq86 [organism=Eladia saccula] [Elad1] 18S ribosomal RNA gene, partial sequence; internal transcribed spacer 1, 5.8S ribosomal RNA gene, and internal transcribed spacer 2, complete sequence; and 28S ribosomal RNA gene, partial sequence

AAGTCGTAACAAGGTTTCCGTAGGTGAACCTGCGGAAGGATCATTACCGAGTGAGGGCCCTCTGGGTCCAACCTCCCACCCGTGTTTATCGTACCTTGTTGCTTCGGCGGGCCCGCCTCACGGCCGCCGGGGGGCTTCTGCCCCCTGGCCCGCGCCCGCCGAAGACACCATTGAACGCTGTCTGAAGATTGCAGTCTGAGCAATTAGCTAAATAAGTTAAAACTTTCAACAACGGATCTCTTGGTTCCGGCATCGATGAAGAACGCAGCGAAATGCGATACGTAATGTGAATTGCAGAATTCAGTGAATCATCGAGTCTTTGAACGCACATTGCGCCCCCTGGTATTCCGGGGGGCATGCCTGTCCGAGCGTCATTGCCGCCCTCAAGCACGGCTTGTGTGTTGGGCCTCCGTCCTCCTCCCGGGGGACGGGCCCGAAAGGCAGCGGCGGCACCGCGTCCGGTCCTCGAGCGTATGGGGCTTCGTCACCCGCTCTTGTAGGCCCGGCCGGCGCTTGCCACACATCAATCTTTTTTCCAGTTGACCTTCGGATCAGGTAGGGATACCCGCTGAACTTAA

SUB6288079 Seq87        MN450653
>Seq87 [organism=apiotrichum dulcitum] [Apio1] 18S ribosomal RNA gene, partial sequence; internal transcribed spacer 1, 5.8S ribosomal RNA gene, and internal transcribed spacer 2, complete sequence; and 28S ribosomal RNA gene, partial sequence

AAGGATCATTAGTGATTGCCATCTTGGCTTAAACTATATCCATCTACACCTGTGAACCGTTTGATTGAATCTTCTGATTCAATTTTACAAACATTGTGTAATGAACGTCATTAGATCATAACAAAAAAAAACTTTTAACAACGGATCTCTTGGCTCTCGCATCGATGAAGAACGCAGCGAAATGCGATAAGTAATGTGAATTGCAGAATTCAGTGAATCATCGAATCTTTGAACGCAACTTGCGCTCTCTGGTATTCCGGAGAGCATGCCTGTTTGAGTGTCATGAAATCTCAACCATTAGGGTTTCTTAATGGCTTGGATTTGGAGGTTGCCATTCTAAATGGCTCCTCTTAAAGGAGTTAGCAAGTTTTACTATTGCTATCTGGCGTAATAAGTTTCGCTGGAATGGTATTGTGAAGCGTGCTTCTAATCGTCTTCGGACAATTACTTTGACTCTGGCCTCAAATCAGGTAGGACTACCCGCTGAACTTAA

SUB6288079 Seq88        MN450654
>Seq88 [organism=Wardomyces dimerus] [Ward1] 18S ribosomal RNA gene, partial sequence; internal transcribed spacer 1, 5.8S ribosomal RNA gene, and internal transcribed spacer 2, complete sequence; and 28S ribosomal RNA gene, partial sequence

AAGTCGTAACAAGGTCTCCGTTGGTGAACCAGCGGAGGGATCATTACCGAAGTTACCCTTCAAAACCCATTGTGAACCATACCTCTTGCCGCGTTGTCTCGGCGGGAGGCGCTGGGGCTTCGGCTCCGCGTCCCCGCCGGCAGCGCCAAACTCTAAACTGCAAAGCGTACTGCATGTTCTGATTTACAAAAAAAACAAGTTAAAACTTTCAACAACGGATCTCTTGGTTCTGGCATCGATGAAGAACGCAGCGAAATGCGATAAGTAATGTGAATTGCAGAATTCAGTGAACCATCGAATCTTTGAACGCACATTGCGCCCGGCAGTAATCTGCCGGGCATGCCTGTCCGAGCGTCATTTCTGCCCTCGAGCGCGGTTAGCTTGCTATCCGCTGCCCGGTGTTGGGGCGCTACGGTTCCAGGGCCTCGTGCCCTGTCCGTAGGCCCCGAAATGAAGTGGCGGTCCCGCCGCGGCGCCCCCTGCGTAGTAGTCTTACAACTCGCATCGGGACCCGGTGGAGGCCAGCCGTCAACCCATCTTTTTTATGGTTTGACCTCNGATCANGTAGGGTTACCCGCTGAACTTAA

SUB6288079 Seq89        MN450655
>Seq89 [organism=Trichomaceae sp.] [Trima1] 18S ribosomal RNA gene, partial sequence; internal transcribed spacer 1, 5.8S ribosomal RNA gene, and internal transcribed spacer 2, complete sequence; and 28S ribosomal RNA gene, partial sequence

AAGTCGTAACAAGGTTTCCGTAGGTGAACCTGCGGAAGGATCATTACCGAGTGCGGGTCCCCTCGCGGGGCCCAACCTCCCACCCGTGTCTACCGTCACCTGTTGCTTCGGCGGGCCCGCCTTCGTGCCGCCGGGGGGCCCTCTGCGCCCCCGGGCCCGCGCCCGCCGAGGACTCTAGGAACACTGAATGAAGGATGCCGTCTGAGTCAACGACACAATCGTTAAAACTTTCAACAACGGATCTCTTGGTTCCGGCATCGATGAAGAACGCAGCGAAATGCGATAAGTAATGTGAATTGCAGAATTCCGTGAATCATCGAATCTTTGAACGCACATTGCGCCCCCTGGTATTCCGGGGGGCATGCCTGTCCGAGCGTCATTACTGCCCATCAAGCCCGGCTTGTGTGTTGGGTCGACGTCCCCTCCCTCGCGGGGGGACGGACCCGAAAGGCAGTGGCGGTGCCGCGCCGGTGCTTCGAGCGTATGGGGCTTTGTCACCCGCTCCGGAGGCCTGGCCGGCGCCCGTCGTCCCCACACTATTTTCTCGTGACCTCGATCAGTAGGGATACCCCTGAACTTAA

SUB6288079 Seq90        MN450656
>Seq90 [organism=Doratomyces sp.] [Dora1] 18S ribosomal RNA gene, partial sequence; internal transcribed spacer 1, 5.8S ribosomal RNA gene, and internal transcribed spacer 2, complete sequence; and 28S ribosomal RNA gene, partial sequence

GCGGAGGAATCATTACCGAAGTTACTTCAAAACCCACTGTGAACCTTACCTCTTGCCGCGTTGTCTCGGCGGGAGGCGGTGGGCGTCGCGCGCCCTAGCGGGCCGGTGCCGCTCCCGTCCCCGCCGGCGGCGCCAAACTCTAAATTTACAGCGGACTGTACGTTCTGATTTACAAAAAAAACAAGTTAAAACTTTCAACAACGGATCTCTTGGTTCTGGCATCGATGAAGAACGCAGCGAAATGCGATAAGTAATGTGAATTGCAGAATTCAGTGAACCATCGAATCTTTGAACGCACATTGCGCCCGGCAGTAATCTGCCGGGCATGCCTGTCCGAGCGTCATTTCTACCCTCGAGCGAGTCCAGCATCTAGGTGCTGGGCCTCGCCCGGCGTTGGAGCACTACGGGAAGCCCTGTGCTGTCCCCCGTAGGCCCCGAAATGAAGTGGCGGTCCTGCCGCGGCGCCCCCTGCGTAGTATAACAGCTCGCTTCGGGACCCGGTGGAGGCCTGCCGTCAAACCT

SUB6288079 Seq91        MN450657
>Seq91 [organism=Cosmospora sp.] [Cosmo1] 18S ribosomal RNA gene, partial sequence; internal transcribed spacer 1, 5.8S ribosomal RNA gene, and internal transcribed spacer 2, complete sequence; and 28S ribosomal RNA gene, partial sequence

AAGTCGTAACAAGGTCCGTTGGTGAACAGCGGAGGGATCATTACTGAGTTACAAACTCCCAAACCCGTGTGAACCTACCACTGTTGCTTCGGCGGCCACGCCCCGGGAGCCACCTGCGCCCCCCGGATCCAGGCGTCCGCCGGAGGACCCAAACTCTTGATTTTACAGAGAATCTTCTGAGTATAATACACAATGAATCAAAACTTTCAACAACGGATCTCTTGGTTCTGGCATCGATGAAGAACGCAGCGAAATGCGATAAGTAATGTGAATTGCAGAATTCAGTGAATCATCGAATCTTTGAACGCACATTGCGCCCGCCAGTATTCTGGCGGGCATGCCTGTCCGAGCGTCATTTCAACCCTCGGGGGCCCCTTTCCAGGGGCCACCGGTGTTGGGGATCGCCCGCCGCTCCAGGCGGTCGTCCCCCAAAAGTATTGGCGGTCTCGCTGTAGCCTCCTCTGCGCAGTAGCTATTGTTCTCGCAGGCGGAGCGCGGCGCGGCCTCGCCGTTAAACCCCCAACTTTCACAAGGTTGACCTCGGATCAGGTAGGAATACCCGCTGAACTTAA

SUB6288079 Seq92        MN450658
>Seq92 [organism=Mucor hiemalis] [Mucor1] 18S ribosomal RNA gene, partial sequence; internal transcribed spacer 1, 5.8S ribosomal RNA gene, and internal transcribed spacer 2, complete sequence; and 28S ribosomal RNA gene, partial sequence

AAGTCGTAACAAGGTTTCCGTAGGTGAACCTGCGGAAGGATCATTAAATAATTTAGATGGCCTTTGCTAGTTTTCTAGCGAATGGTTCATTCTTTTTTACTGTGAACTGTTTTAATTTTTCAGCGTCTGAGGAATGTCTTTTAGCCATAGGGATAGGCTACTAGAATGTTAACCGAGCTGAAAGTCAGGCTTAGGCCTGGTATCCTATTAATTATTTACCAAAAGAATTCAGTATTATAATTGTAACATAAGCGTAAAAAACTTATAAAACAACTTTTAACAACGGATCTCTTGGTTCTCGCATCGATGAAGAACGTAGCAAAGTGCGATAACTAGTGTGAATTGCATATTCAGTGAATCATCGAGTCTTTGAACGCAACTTGCGCTCAATGGTATTCCATTGAGCACGCCTGTTTCAGTATCAAAAACACCCCACATTCATAATTTTGTTGTGAATGGAAATGAGAGTTTCGGCTTTATTGCTGAATTCTTTAAAATTATTAGGCCTGAACTATTGTTCTTTCTGCCTGAACATTTTTTTAATATAAAGGAATGCTCTAGTAAAAAGACTATCTCTGGGGCCTCCCAAATAAATCATTCTTAAATTTGATCTGAAATCAGGCGGGATTACCCGCTGAACTTAA

SUB6288079 Seq93        MN450659
>Seq93 [organism=Hypholoma fasciculare] [Hyph1] 18S ribosomal RNA gene, partial sequence; internal transcribed spacer 1, 5.8S ribosomal RNA gene, and internal transcribed spacer 2, complete sequence; and 28S ribosomal RNA gene, partial sequence

AACCCTGCGGAAGGATCATTATTGAATAAACCTGGGCTTGGTTGATGCTGGTCTTTTCGGATACATGTGCACACCTTGTCATCTTTATATCTCCACCTGTGCACCTTTTGTAGACCTGGATTCAACTTTCCGAGGAAACTCGGTTGTGAGGAATTGCTTAATAGGCTTTCCTTGTTAGTTTCCAGGGCTATGTTTTCATATACACCTTAAGAATGTAACAGAATGTCATTATTAGGCTTAATTGCCTTATAAACTATATACAACTTTCAGCAACGGATCTCTTGGCTCTCGCATCGATGAAGAACGCAGCGAAATGCGATAAGTAATGTGAATTGCAGAATTCAGTGAATCATCGAATCTTTGAACGCACCTTGCGCTCCTTGGTATTCCGAGGAGCATGCCTGTTTGAGTGTCATTAAATTCTCAACCTTTATTAACTTTTTGGTTAGTAAGTGGATTGGAAGTGGGGGCATGTTGGTTTCTTCATTGAAATAAACTCCCCTGAAATGCATTAGCTGGTTGCCTTGTGCGAACATGTCTATTGGTGTGATAATTATCTACGCCGTGGGCTATTTGCCGTTTATAGCACTGCTTATAATCGTCTGTTCATTCAGACAATATATGACAATTTGACCTCAAATCAGGGTAGGACTACCCGCTGAACTTAA

SUB6288079 Seq94        MN450660
>Seq94 [organism=Hypoxylonserpens] [Hyph2] 18S ribosomal RNA gene, partial sequence; internal transcribed spacer 1, 5.8S ribosomal RNA gene, and internal transcribed spacer 2, complete sequence; and 28S ribosomal RNA gene, partial sequence

AAGTCGTAACAAGGTCTCCGTTGGTGAACCAGCGGAGGGATCATTACAGAGTTATCAAACTCCCAAACCCATGTGAACATACCTCGCGTTGCCTCGGCAGGTGGCGTCTCGCCCCGTAAGAACCTACCCTGTAGGACCTTACCCGGTAGACGACCCTGCCGACGGCCCCCGAAACTCTGTTTTATAGCATTAAACTTCTGAAAATATAACTAAATAAGTTAAAACTTTCAACAACGGATCTCTTGGTTCTGGCATCGATGAAGAACGCAGCGAAATGCGATAAGTAATGTGAATTGCAGAATTCAGTGAATCATCGAATCTTTGAACGCACATTGCGCCCACTAGTATTCTGGTGGGCATGCCTGTTCGAGCGTCATTTCAACCCTTAAGCCCCTGTTGCTTAGCGTTAGGAGCCTACCGGAACTCTCTGGTAGCTCCCCAAAGTCAGTGGCGGAGCCGGTTCGCACTCCAGACGTAGTAGCTTTTACACGTCGCCTGTAGCGCGGGCCGGTCCCCTGCCGTAAAACACCCCAATTTTTATAGGTTGACCTCGGATCAGGTAGGAATACCCGCTGAACTTAA

**SUB6288079 Seq95        MN450661
>Seq95 [organism=Tomentella] [Tom1] 18S ribosomal RNA gene, partial sequence; internal transcribed spacer 1, 5.8S ribosomal RNA gene, and internal transcribed spacer 2, complete sequence; and 28S ribosomal RNA gene, partial sequence**

CACGCTCTGTTTACACATCCACTCACACCTGTGCACCCTCTGTAGTTCTATGGCCTGGGGGGCTCCGTCCTCCTGCTGTGGCTCTACATCTTTACACACACACACAGTGAAAAAGTCTCATGGAATGTATGTGGCGTTTAACGCAATGAAATACAACTTTCAGCAACGGATCTCTTGGCTCTCGCATCGATGAAGAACGCAGCGAAATGCGATAAGTAATGTGAATTGCAGAATTCAGTGAATCATCGAATCTTTGAACGCACCTTGCGCCCTTTGGCCATTCCGAAGGGCATGCCTGTTTGAGTATCATGAACACCTCAACTCTCATGGCTTGCCATGATGAGTTTGGACTTTGGGGGCCTTGCTGGCCTGTGAGTCAGCTCCTCTCAAATGAATTAGCTTGCCGGTGTTTGGTGGCATCACAGGTGTGATAACTATCTACGCTTGTGGTTTTCCACCCAGGTAACCTTCAGCAATGGAGGTTCACCGGGAGCTCATAAAGTGTCTCTCCTCTCCGAGGACGGATTTTT

SUB6288079 Seq96        MN450662
>Seq96 [organism=Sordariomycetes] [Sord1] 18S ribosomal RNA gene, partial sequence; internal transcribed spacer 1, 5.8S ribosomal RNA gene, and internal transcribed spacer 2, complete sequence; and 28S ribosomal RNA gene, partial sequence

CCATTGTGAACGTTACCTATCCCGTTGCTTCGGCGGGCGGCCCGGGCCCCGTGCCCGGCGCCCCCCGGCCCCTCGCGGGCGCCCGCCGGAGGTAAACCAAACTCTTGAATTGTATGGCCTCTCTGAGTCTTCTGTACTGAATAAGTCAAAACTTTCAACAACGGATCTCTTGGTTCTGGCATCGATGAAGAACGCAGCGAAATGCGATAAGTAATGTGAATTGCAGAATTCAGTGAATCATCGAATCTTTGAACGCACATTGCGCCCGCCAGTATTCTGGCGGGCATGCCTGTTCGAGCGTCATTTCAACCATCAAGCCCCCGGCTTGTGTTGGGGACCTGCGGCTGCCGCAGGCCCTGAAAACCAGTGGCGGGCTCGCTAGTCACTCCGAGCGTAGTAATACATCTCGCTCA
